# Beyond crying: Protocol for a multimodal assessment of suspected prolonged pain in infants using infrared thermal imaging, behavioral assessment and physiological biomarkers (NEOTHERM)

**DOI:** 10.1371/journal.pone.0361344

**Published:** 2026-11-12

**Authors:** Camille Perrolet, Sarah Bahmd-Alves, Isabelle Carchon, Juliette Berthier, Joëlle Provasi

**Affiliations:** 1 Laboratoire Cognitions Humaine et Artificielle (CHArt), École Pratique des Hautes Études (EPHE), Université Paris Sciences et Lettres (PSL), Aubervilliers, France; 2 Collège Européen d’Enseignement Supérieur de l’Ostéopathie (CEESO), Saint-Denis, France; PLOS: Public Library of Science, UNITED KINGDOM OF GREAT BRITAIN AND NORTHERN IRELAND

## Abstract

Prolonged pain during early infancy may affect neurodevelopment, yet no consensus definition or reference standard exists for its assessment. Current clinical evaluation relies primarily on behavioral tools such as the Neonatal Pain and Discomfort Scale (EDIN; Échelle de Douleur et d’Inconfort du Nouveau-né) which remain indirect and lack objectivity and sensitivity. Infrared thermal imaging (ITI) represents a promising, non-contact technique that captures skin-temperature patterns related to cutaneous perfusion and autonomic vasomotor regulation, potentially providing complementary and objective physiological information.

Materials and methods

NEOTHERM is a multicenter, prospective, longitudinal, observational pilot study conducted in France. Eighty infants will be classified into an excessive-crying cohort (n = 40) or a typical-crying cohort (n = 40) using a 7-day crying diary and assessed twice, approximately two months apart. EDIN, ITI, and hair cortisol concentration will characterize pain-related behavior, thermo-autonomic parameters, and cumulative cortisol exposure; General Movements Assessment and Bayley-4 will assess motor organization and early neurodevelopment. The primary analysis will estimate associations between prespecified ITI-derived parameters and EDIN scores at the first assessment. Secondary analyses will examine associations with cortisol, motor, and developmental outcomes and changes over time.

Discussion and conclusion

NEOTHERM will evaluate the feasibility, reproducibility, and convergent validity of ITI-derived thermal parameters within a multimodal framework. Because thermal responses are not pain-specific, ITI will be investigated as a complementary physiological measure rather than as a standalone diagnostic tool. The study will provide effect-size estimates and methodological data for future confirmatory research.

Trial registration

ClinicalTrials.gov (NCT07693179).

## 1. Introduction

Infants, including those born preterm, are capable of experiencing pain from birth [1,2]. Pain is a complex, multidimensional sensory and emotional experience [3]. In nonverbal populations, particularly neonates, pain assessment remains a major clinical challenge [2,4], and its presence must be inferred from behavioral and physiological manifestations, including autonomic changes [3,5]. Because these manifestations are nonspecific and may also reflect other forms of distress, pain may be underrecognized, misattributed, and consequently undertreated [2,6].

This uncertainty is particularly relevant during early infancy, when nociceptive pathways are functional but descending inhibitory systems remain immature, resulting in reduced pain modulation and widespread neural activation [7–9]. These developmental characteristics may heighten pain sensitivity and increase vulnerability to the effects of repeated or prolonged nociceptive input [7,9,10]. Exposure to repeated or prolonged pain-related stress during this period has been associated with altered brain development and adverse neurodevelopmental outcomes [11–13].

Most neonatal pain research has focused on acute procedural or postoperative pain [2,14], whereas prolonged pain remains underexplored and lacks a consensus definition in neonates and young infants [15–17]. The conventional three-month threshold for chronic pain is poorly suited to infants younger than three months [18,19]. In NEOTHERM, prolonged pain is therefore defined as a persistent or recurrent pain-related state during the first three months of life, rather than a diagnosis defined by a fixed duration threshold.

Prolonged pain may manifest through nonspecific changes in facial activity, sleep, muscle tone or motor behavior, social engagement, responsiveness, crying, and consolability, ranging from irritability to behavioral withdrawal [15,16]. Excessive crying is particularly relevant because it is a frequent reason for healthcare consultation during early infancy [20,21]. Although often regarded as a benign and transient developmental phenomenon [20,21], excessive crying may also signal underlying discomfort, sustained distress [22], or prolonged pain [23]. Its interpretation remains challenging, however, because crying can arise from a wide range of physiological, developmental, relational, and environmental factors [20,22]. The clinical challenge is therefore to identify, among infants with excessive crying, those with a broader behavioral and physiological profile consistent with suspected prolonged pain.

Pain assessment in neonates and young infants therefore relies on behavioral and physiological indicators, used separately or combined within multidimensional scales [4,6,16]. Among available behavioral instruments, the Neonatal Pain and Discomfort Scale (EDIN; *Échelle de Douleur et d’Inconfort du Nouveau-né*) was specifically developed to assess prolonged neonatal pain. It rates five domains: facial activity, body movements, sleep quality, quality of contact, and consolability over a sustained observation period [24,25]. Widely used in clinical practice [16], the EDIN has demonstrated satisfactory internal consistency, construct validity, and inter-rater reliability [24–26]. Although some psychometric properties require further investigation [4], its clinical feasibility, longitudinal behavioral focus, and available validation data support its selection as the behavioral comparator in NEOTHERM.

No single measure fully captures prolonged pain [2,14,27]. Behavioral scores depend on observer judgment and may be influenced by gestational age, neurological maturation, sleep–wake state, clinical condition, and environmental context [4,28,29]. Persistent pain may also attenuate the same behaviors on which these scores rely, with some infants showing hyporeactivity or withdrawal despite ongoing pain [15,17,30].

Physiological indicators, including blood pressure, oxygen saturation, heart rate variability, and skin conductance [6,27], provide instrument-derived data, less subject to human subjectivity but remain nonspecific, may be difficult to collect outside hospital settings, and may also become attenuated during prolonged pain [14,16,17]. Multidimensional scales integrate behavioral and physiological indicators to capture complementary components of the pain response [2,4,16,31].

These limitations support an integrative approach in which behavioral assessment is complemented by biological and autonomic measures reflecting distinct components of the pain-related response [2,15].

Within multimodal approaches, hair cortisol concentration provides a non-invasive, retrospective estimate of cumulative cortisol exposure and longer-term hypothalamic–pituitary–adrenal (HPA) axis activity over the period represented by the sampled hair [27,32–34]. Painful stimuli engage the HPA axis, and persistent or repeated pain may contribute to longer-term alterations in stress-system regulation [35,36]. Although not specific to pain, hair cortisol concentration may therefore capture a cumulative neuroendocrine component of prolonged pain-related states and complement behavioral and autonomic measures [32]. Unlike salivary or plasma cortisol, which reflect short-term concentration influenced by circadian variation and recent stress [35], hair sampling allows for a stable and cumulative assessment of hormonal exposure [32,33,37]. Hair collection is non-invasive and suitable for neonatal populations when sufficient hair is available; however, quantification by liquid chromatography–tandem mass spectrometry (LC–MS/MS) requires specialized laboratory processing and does not provide immediate clinical feedback [38,39].

Taken together, the limitations of current assessment approaches highlight the need for rapid, non-invasive, instrument-derived physiological measures that can complement behavioral assessment within a multimodal framework of prolonged pain in infants. Infrared thermal imaging (ITI) is a promising candidate in this context. It enables real-time, contact-free measurement of skin temperature distribution, providing indirect information on cutaneous perfusion and autonomic vasomotor regulation [40,41]. Associations between thermal imaging and laser Doppler perfusion measurements support the physiological relevance of thermal data [42]. Under standardized physiological acquisition conditions, homologous contralateral body regions generally display broadly symmetrical thermal patterns [43–45]. Right–left asymmetry has therefore been used as a potential marker of altered autonomic or vascular regulation [46,47]. In adults and children, pain-related conditions have been associated with regional temperature changes and bilateral thermal asymmetries. However, the magnitude and direction of these changes vary across neuropathic pain [46,48,49], inflammatory conditions [50], musculoskeletal pain [47,51], and complex regional pain syndrome [52,53]; accordingly, no pain-specific pattern or universal diagnostic threshold has been established. Associations with pain intensity and preliminary diagnostic performance of composite indices such as the Thermal-Associated Pain Index have been reported in proof-of-concept studies [54]. Depending on the clinical context, thermal analysis may focus on bilateral asymmetry [46,55], local or regional temperature differences [56], core-to-peripheral temperature gradients [57,58], or regional facial thermal changes [59–61].

Facial thermography appears particularly relevant because several facial regions are highly responsive to stress-related autonomic activation [59,62]. In adults and children, the nasal tip is one of the most frequently studied facial markers of acute sympathetic arousal and often shows a delayed decrease in temperature during active psychological stress [59,63,64]*,* whereas the periorbital, perioral, and frontal regions may exhibit distinct thermal responses depending on the type, intensity, and temporal phase of the stimulus [59–61]. Recent adult proof-of-concept evidence supports this complementary role [61]: thermal–red–green–blue (RGB) image fusion improved pain-intensity estimation compared with RGB imaging alone, particularly in individuals with limited facial expression, and thermal responses preceded visible behavioral manifestations. These findings support evaluation of ITI alongside behavioral measures.

In neonatal and infant research, ITI has been used to study temperature distribution [41,65] and thermoregulation [66], clinical adaptation [67,68], pathological conditions such as necrotizing enterocolitis [69] and autonomic responses to non-painful stressors [70,71]. These findings demonstrate the feasibility of ITI in early infancy and its sensitivity to autonomic activation, but not its specificity for pain. To our knowledge, ITI has not been prospectively evaluated against a recognized neonatal pain scale, and its application to prolonged pain-related states in young infants remains unexplored.

If its reproducibility, construct validity, and added value beyond existing behavioral and clinical measures are demonstrated, ITI could provide rapid, non-invasive, instrument-derived physiological information that is less dependent on observer judgment and could facilitate repeated measurements across neonatal, outpatient, and non-clinical settings. Such validation could support future efforts to identify prolonged pain earlier and monitor it over time. Until such evidence is available, thermal parameters should be considered complementary autonomic correlates rather than independent diagnostic markers of pain. NEOTHERM therefore evaluates EDIN together with hair cortisol concentration and ITI-derived parameters, representing behavioral expression, cumulative neuroendocrine, and autonomic responses. Examining their convergence and complementarity may characterize suspected prolonged pain more comprehensively than any individual measure, without assuming that they assess the same underlying construct.

Beyond improving its assessment, identifying prolonged pain in early infancy may also help elucidate its potential neurodevelopmental correlates [72,73]. Prolonged pain may act as a sustained sensory stressor capable of affecting early motor organization [9,74], especially during a period when sensory inputs and motor outputs are closely coupled [75,76]. Supporting this biological plausibility, repeated early-life pain exposure has been associated with atypical brain development [77], altered functional connectivity [75,76], and later neurodevelopmental impairment [13,74,78]. Because the quality of spontaneous general movements reflects the integrity of the developing nervous system, the General Movements Assessment provides a relevant tool for exploring these potential early correlates [79,80]. However, direct evidence linking prolonged pain-related states to alteration in spontaneous motor activity or early neurodevelopment remains limited [81,82].

NEOTHERM investigates whether ITI can identify candidate thermo-autonomic signatures associated with suspected prolonged pain in young infants. Thermal parameters are examined within a multimodal framework alongside pain-related behavior (EDIN), cumulative cortisol exposure (hair cortisol concentration), motor organization (GMA), and general development (Bayley-4) [83,84]. The conceptual model presented in Fig 1 considers excessive crying a nonspecific clinical signal potentially associated with prolonged pain, cumulative stress, autonomic dysregulation, and early neurodevelopmental differences. This model guided the study design and outcome selection, positioning thermal signatures as a complementary physiological component rather than an isolated proxy for pain.

**Fig 1:**
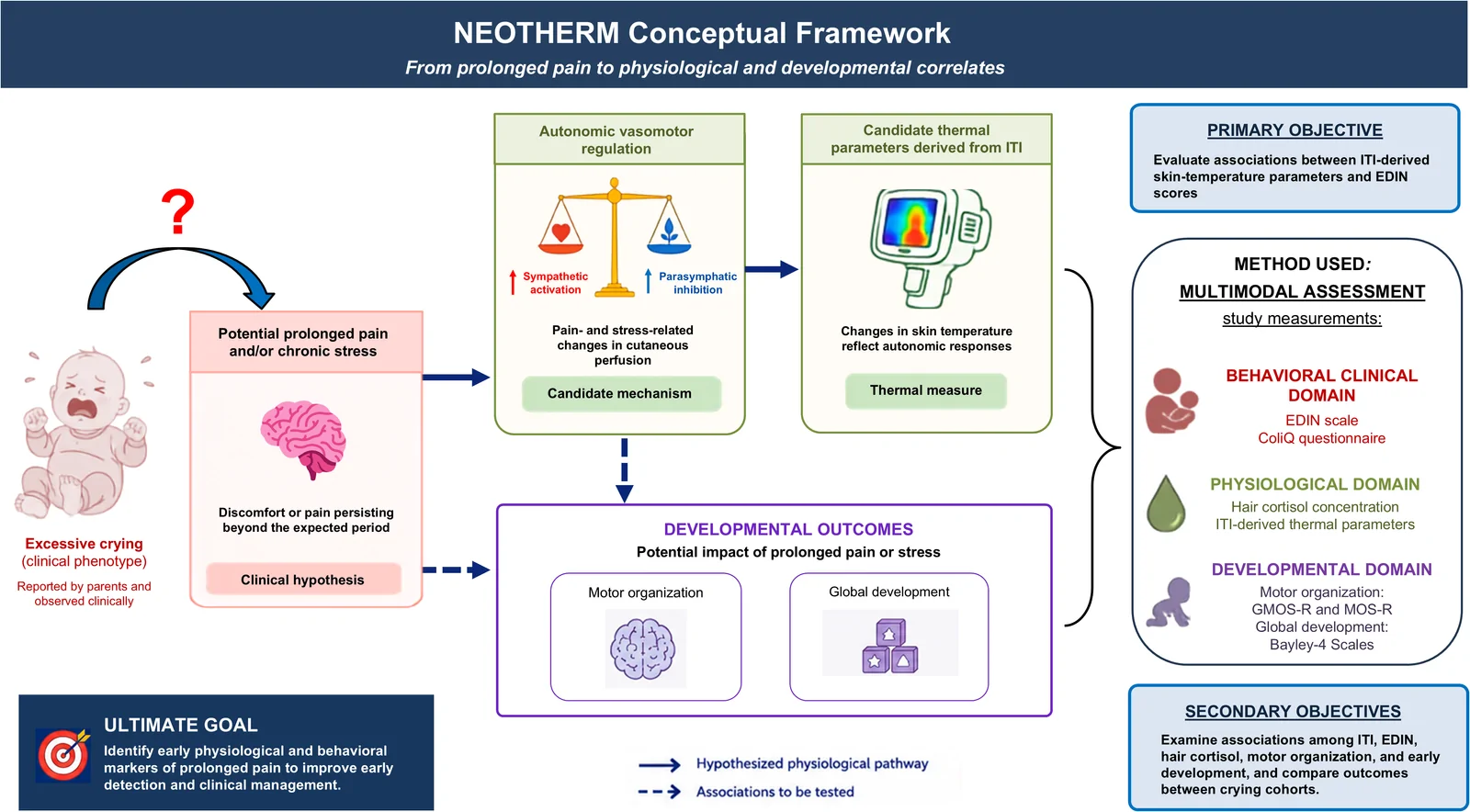
Conceptual framework of the NEOTHERM study. The model presents the hypothesized relationships among excessive crying, suspected prolonged pain, cumulative stress, autonomic vasomotor regulation, candidate ITI-derived thermal parameters, and early neurodevelopment. The study integrates behavioral and clinical measures (EDIN, ColiQ, and a 7-day crying diary), biological and physiological measures (hair cortisol concentration and thermal imaging), and developmental assessments (General Movements Assessment and Bayley-4). Solid arrows represent hypothesized physiological pathways; dashed arrows represent associations to be tested and data-integration links.

The primary objective is to estimate associations between ITI-derived thermal parameters and EDIN scores, thereby evaluating whether ITI provides physiological information complementary to behavioral assessment of suspected prolonged pain in infants. Secondary objectives are to (1) examine associations among thermal parameters, EDIN scores, and hair cortisol concentrations; (2) compare motor organization and early neurodevelopment between excessive- and typical-crying cohorts; (3) investigate associations between pain-related indicators and developmental outcomes; and (4) explore changes over time. We hypothesize that thermal parameters will covary with EDIN scores and cortisol exposure and that convergent pain-related indicators will be associated with less optimal early motor and developmental functioning.

## 2. Materials and Methods

### 2.1. Study design

NEOTHERM is a multicenter, prospective, longitudinal, observational pilot study with two comparison cohorts assessed between 2 and 14 weeks corrected age (CA). The manuscript follows items relevant to observational cohorts in STROBE [85]; the enrolment and assessment schedule uses an adapted SPIRIT-style format for clarity [86]. The study is a French Category 2 study involving human participants (RIPH2; ID-RCB 2025-A00767-42), sponsored by the Centre Hospitalier de Saint-Denis, approved by the Comité de protection des personnes Île-de-France II, registered at ClinicalTrials.gov (NCT07693179), and provided in full as S1 Appendix: Protocol.

Recruitment is planned from September 2026 to September 2028, with study completion and initial results expected in November 2028. Eighty infants will be included (40 per cohort) and assessed twice, approximately two months apart. Cohorts will be defined by crying duration and the Rome IV criteria for infant colic, defined as recurrent and prolonged episodes of crying, irritability, or agitation in infants younger than 5 months of age, occurring without an identifiable cause, resistant to parental soothing or prevention efforts, and without adverse effects on growth or general health status [87].

### 2.2. Participants and group classification

Infants will be recruited at three Île-de-France centers**.** Eligible participants will be infants scheduled for the T1 assessment at 2–6 weeks post-term CA, born at or after 32 weeks of gestation (GA), with no known neurological or gastrointestinal disease, whose legal guardians are covered by social security and have provided written informed consent. Exclusion criteria will include: major cerebral or chromosomal abnormalities, growth faltering, birth before 32 weeks of GA, absence of legal guardian consent, guardians who do not understand French, or lack of social security coverage.

Cohort classification will use a 7-day caregiver crying diary [88,89] (S2 Appendix). The excessive-crying cohort will comprise infants crying for more than 3 hours/day on at least 3 days per week, with at least two protocol-specified Rome IV-related features (no apparent cause, inconsolability, or associated agitation signs) [21,87]. The typical-crying cohort will comprise infants crying for less than 2 hours/day. This threshold corresponds to the typical developmental trajectories described between 3 and 12 weeks of age [20,90]. Infants with intermediate crying patterns (2–3 hours per day) will be excluded to reduce overlap and bias.

Classification criteria will be prespecified and the T1 classification retained for all longitudinal analyses, regardless of subsequent crying changes. Infants will be individually pair-matched on corrected age, sex, gestational age at birth, feeding modality, and maternal parity.

### 2.3. Study setting

#### 2.3.1. Research sites.

Recruitment and data collection will take place at three centers located in the Île-de-France region, including a neonatal unit (Department of Neonatology, Centre Hospitalier de Saint-Denis); a neonatal psychiatry unit (Department of Perinatal Psychiatry, Centre Hospitalier de Saint-Denis), and a medical-psychological center (Centre Médico-Psychologique Périnatalité et Petite Enfance Brantôme, Paris). Assessments will be conducted in dedicated rooms that meet the environmental conditions required for thermal imaging as described below.

#### 2.3.2. Recruitment procedure and consent.

Clinical teams will provide potentially eligible families with study information and the consent documents (S3 Appendix). A research-team telephone call will answer questions and, when appropriate, schedule the enrolment visit.

#### 2.3.3. Visit schedule.

Infants will participate in three study phases: an enrollment visit, followed by the T1 assessment conducted at 2–6 weeks post-term CA and the T2 assessment conducted at 10–14 weeks post-term CA.

At enrolment, guardians will provide written consent and complete sociodemographic, pregnancy, birth, and medical-history questions (S4 Appendix) followed by a questionnaire assessing the impact of infant crying on the quality of life of both the infant and their family (QB: ColiQ®).

The investigator will provide the guardians with the first crying diary, in which they will record the infant’s crying, sleeping, and feeding periods. Guardians will be instructed to complete the diary over the 7 days preceding the T1 visit.

Infants will be assessed at both the T1 and T2 visits. The procedures will be identical for both visits and for both groups, and will include: (1) a multimodal assessment of pain and stress comprising the EDIN scale, ITI, and hair cortisol sampling; and (2) a psychomotor assessment, including the GMA and administration of the Bayley-4 scales. After T1, families will complete a second 7-day crying diary before T2 and record three 3–5-minute General Movements videos on days 15, 30, and 45 using standardized GM Trust instructions.

#### 2.3.4. Timeline.

The NEOTHERM study follows a prospective longitudinal design, as presented in Fig 2.

**Fig 2:**
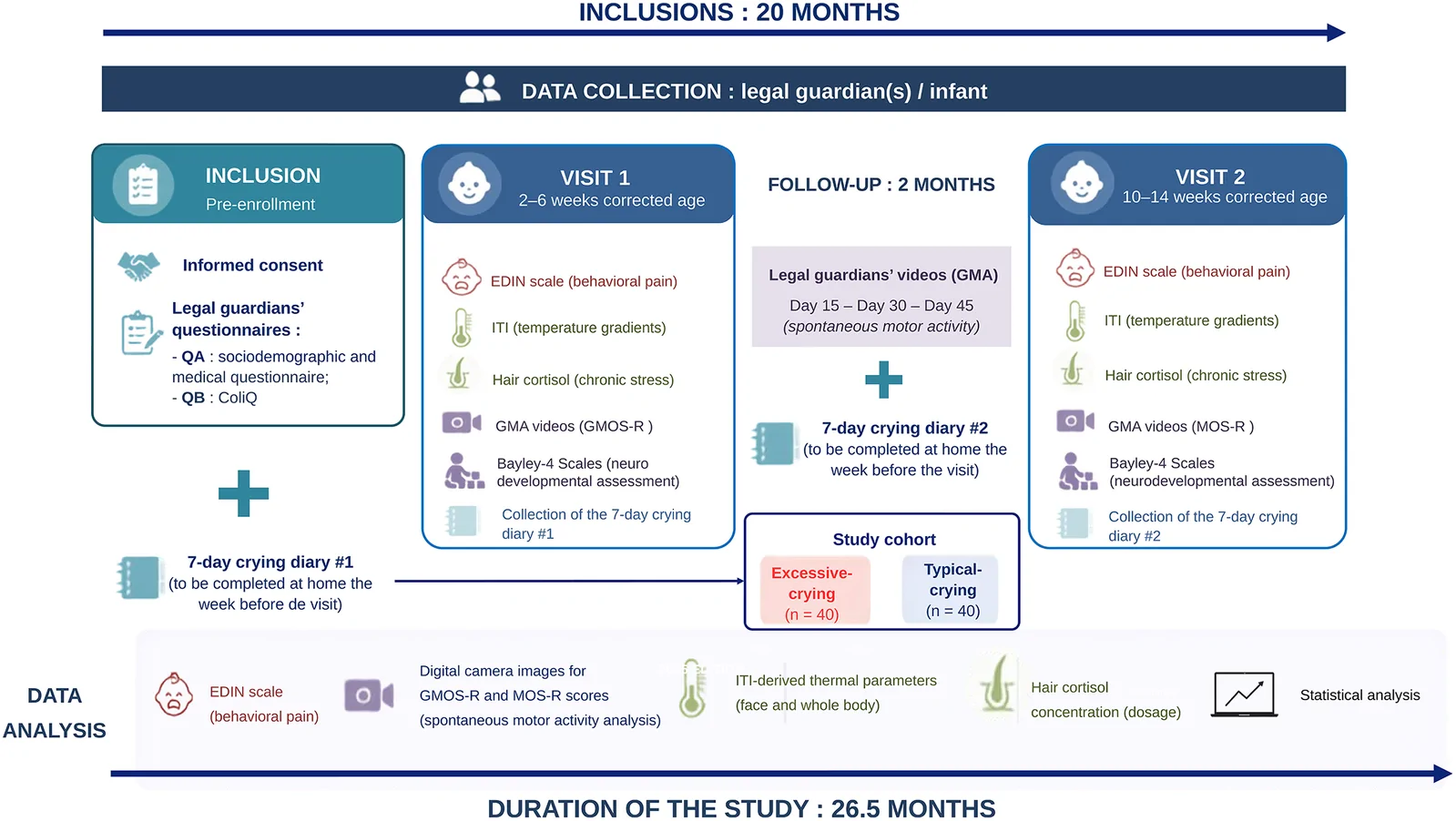
Overview of the NEOTHERM study design. After enrollment, infants undergo two assessments separated by an approximately two-month follow-up period. Group classification is performed at T1, after collection of the first 7-day crying diary. Outcome measures include EDIN scores, ITI-derived thermal parameters, hair cortisol concentrations, General Movements Assessment (GMOS-R/MOS-R), Bayley-4 scores, and guardian-recorded videos of spontaneous motor activity.

**Table 1:** Adapted timeline for enrolment, assessments, and procedures in the NEOTHERM study.

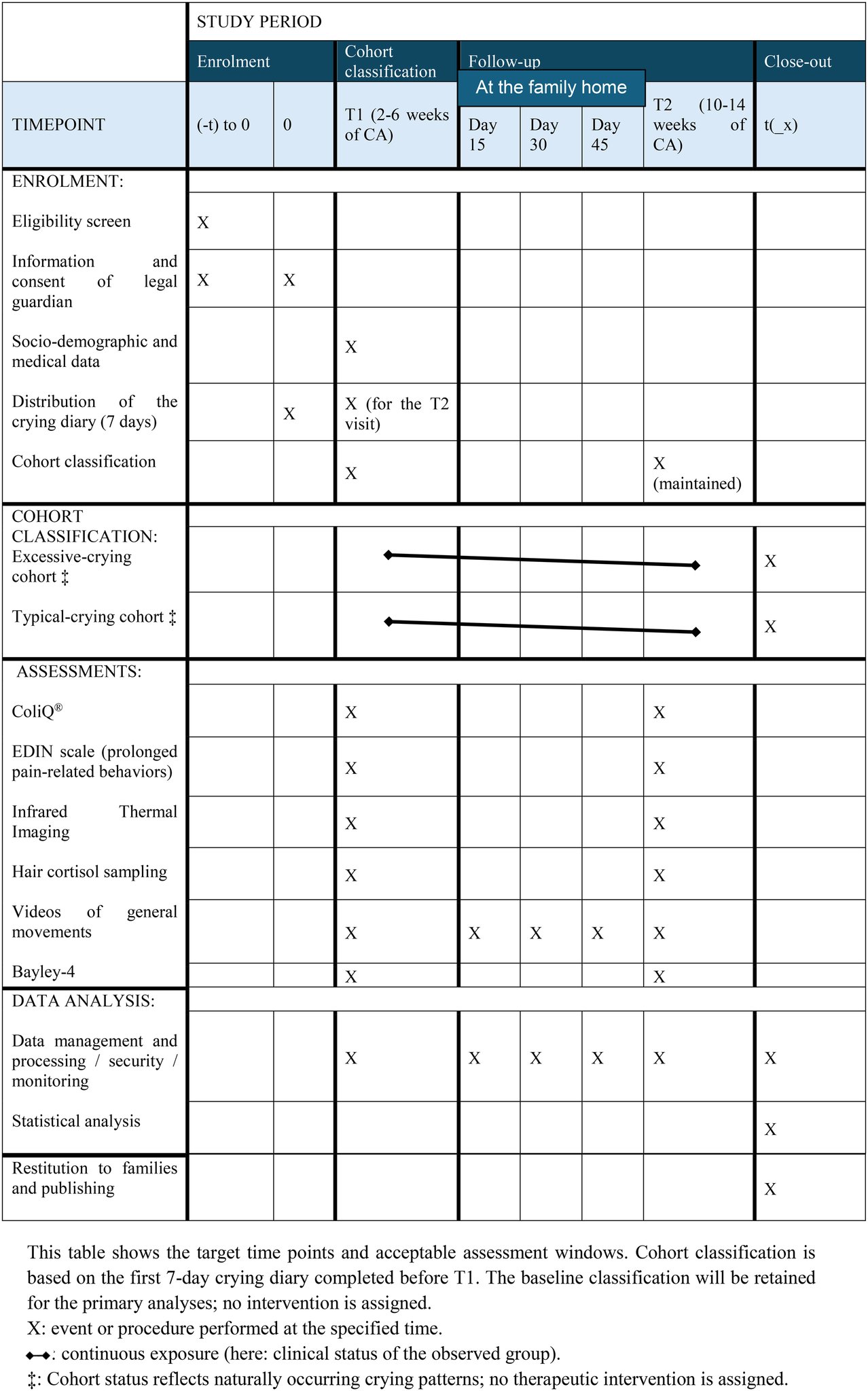

The schedule of enrolment, assessments, and study procedures is presented in the adapted timeline below (Table 1).

### 2.4. Pain and stress assessment

The infant will be undressed to the diaper to allow for an acclimatization period to the environmental conditions required for reliable thermal measurements [91,92]. Clinical assessments, including collection of the crying diary, completion of guardian-reported questionnaires, determination of group classification, and administration of the EDIN scale, will be performed by two trained clinical investigators of the research team (IC or CP). At T1, infants will be classified into the excessive-crying or typical-crying cohort on the basis of the preceding 7-day crying diary; this baseline classification will be retained at T2. To minimize assessment bias, separate blinded investigators will process thermal and motor recording (see Section 2.7).

The multimodal assessment integrates behavioral, physiological, biological, and developmental measures. The measures, their respective domains, and the investigators responsible for data collection or assessment are summarized in Table 2. Thermal image processing and motor assessments will be performed at a later stage using pseudonymized recordings and datasets.

**Table 2:** Multimodal assessment measures and investigator responsibilities in the NEOTHERM study.

Measure	Domain assessed	Data collection/ administration	Processing/ assessment
7-day crying diary	Crying duration and pattern; cohort classification	CP, IC	CP, IC
ColiQ®	Impact of infant crying on infant and family quality of life	CP, IC	CP, IC
EDIN scale	Prolonged pain-related behavioral expression	CP, IC	CP, IC
Infrared thermal imaging (ITI)	Cutaneous thermal patterns reflecting autonomic vasomotor regulation	CP, IC, JP, SBA, JB	JB, SBA
Hair cortisol concentration	Cumulative cortisol exposure/ longer-term HPA-axis activity	CP, IC, JP, SBA	Robert-Debré Hospital laboratory
General Movements Assessment (GMOS-R/MOS-R)	Spontaneous motor organization and early neurological functioning	Research team; guardian-recorded videos at D15, D30, and D45	JP, MDC
Bayley-4	Early cognitive, language, fine-motor, and gross-motor development	CP, IC, JP, SBA	Independent rescoring: JP, SBA, or JB

**Abbreviations:** CP, Camille Perrolet; IC, Isabelle Carchon; JB, Juliette Berthier; SBA, Sarah Bahmd-Alves; JP, Joëlle Provasi; MDC, Maeva Da Cunha; EDIN, Échelle de Douleur et d’Inconfort du Nouveau-né; ITI, infrared thermal imaging; HPA, hypothalamic–pituitary–adrenal; GMOS-R, General Movement Optimality Score–Revised; MOS-R, Motor Optimality Score–Revised.

#### 2.4.1. Neonatal pain and discomfort scale (EDIN).

The EDIN scale will be used to assess prolonged pain-related behaviors. The tool comprises five behavioral items: facial expression, body movements, sleep quality, quality of interaction, and the infant’s ability to be soothed. Each item is scored from 0 to 3, resulting in a total score ranging from 0 to 15 [24].

At each visit, a trained investigator (CP or IC) will integrate caregiver-reported behavior with direct observation during the visit. Sleep quality will rely primarily on caregiver report, while the other items will use both sources. If sources differ, the investigator will document the discrepancy and score the predominant sustained pattern over the reference period. A total score of 5 or higher indicates probable prolonged pain or discomfort [24].

#### 2.4.2. Infrared thermal imaging (ITI).

**Acclimatization phase:**

Infants wearing a diaper will undergo a standardized 15-minute acclimatization period [45,55,70]. EDIN and General Movements recording may occur during this period, but thermal frames will be retained only after skin-temperature stabilization and a prespecified artifact-free interval. The EDIN questionnaire will be administered while the infant is held, whereas General Movements will be recorded after the infant has been placed in the supine position on the mattress, under the standardized conditions required for General Movements Assessment [79], and may overlap with the final part of the acclimatization period and the supine thermal recording. These assessments were deliberately scheduled during the acclimatization phase to optimize study flow while allowing sufficient time for stabilization of skin temperature before thermal image acquisition.

Time since the last feeding, sleep–wake state, crying or agitation, pacifier use, recent physical contact or handling, fever, and medications likely to affect thermoregulation will be recorded, as these factors may influence facial and body temperatures independently of pain [40,91,92].

**Recordings:**

Infants will lie on a standardized mattress covered with clean black cotton material. This setup was selected to minimize environmental thermal reflections that could interfere with thermographic measurements and to optimize thermal contrast during image analysis. Contact, partial occlusion, and other image-quality limitations will be flagged. The setup, camera position, and acquisition sequence will be standardized across participants [92].

Thermal recordings will be acquired using a tripod-mounted FLIR® T630sc infrared thermographic camera (thermal sensitivity: 0.04°C; spatial resolution: 640 × 480 pixels; acquisition rate: 30 frames/s) positioned 1 m above the infant. Emissivity, reflected apparent temperature, focus, ambient inputs, and calibration checks will be prespecified and documented for each session. Recordings will be stored in the native radiometric format to preserve the complete temperature information for subsequent analyses. Two-minute recordings will be obtained in supine and prone positions, with posture changes and events time-stamped.

Recordings will target awake, calm periods with minimal stimulation. Necessary caregiver speech, handling, repositioning, crying, gross movement, sleep transitions, or pacifier use will be time-stamped and considered in quality assessment [60,71]. Infant handling will be minimized throughout the thermal imaging procedure to reduce potential thermal artifacts associated with skin contact. A brief stabilization interval will follow manipulation before analyzable recording resumes.

In accordance with established guidelines for infrared thermography research, recordings will be performed under stable and thermally comfortable environmental conditions (23 °C ± 1 °C; relative humidity: 50–60%) [41,93]. Ambient temperature and relative humidity will be monitored throughout each session using an OMEGA OM-HL-SP-TH thermometer-hygrometer (temperature accuracy: ± 0.5°C; relative humidity accuracy: ± 5%) and logged into the thermographic camera prior to each recording. The setup will be protected from direct sunlight, drafts, windows, and heating sources [41,93].

At the end of the recording session, infants will be returned to their guardians and dressed before proceeding to the subsequent assessment (i.e., hair sampling and Bayley-4).

**Data extraction:**

Two trained investigators (JB and SBA), blinded to cohort allocation and clinical outcomes, will independently process the same thermal recordings. Inter-rater reliability for continuous ROI temperatures will be estimated with a prespecified intraclass correlation coefficient and 95% confidence interval, detailed in the statistical analysis plan.

Thermographic images of the face and body will be segmented into 42 regions of interest (ROIs) based on vascular distribution patterns and previous literature [43,45,61]. These ROIs will include: 36 body ROIs (S5 Table) and 6 facial ROIs (forehead, nasal tip, perioral region, chin, and bilateral inner canthi) (S6 Table).

ROI definitions and thermal metrics will be fixed before extraction and unblinding, to limit post hoc selection and multiplicity-related bias; no ROI or temperature-change direction will be considered pain-specific a priori.

Frames will be selected in FLIR ResearchIR using prespecified criteria for posture, anatomical visibility, movement, occlusion, contact artifact, and temporal window. Homologous ROIs will be extracted from the same frame whenever possible; otherwise, additional eligible frames may be used. Thermographic data extraction will be performed using a customized Python script (developed by JB), enabling semi-automated processing of thermal images. ROIs will be manually delineated by the investigator and the mean, maximum, and minimum temperatures for each ROI will be automatically computed and exported to a single spreadsheet for subsequent statistical analyses and thermal mapping (Fig 3). Prespecified bilateral asymmetries, core-to–peripheral gradients, and intra-facial gradients will also be automatically calculated. Mean ROI temperature will be the principal summary measure; temporal change, slope, and variability will be exploratory when sufficient consecutive frames are available [40,61]. As no standardized nociceptive stimulus is administered in this study, all thermal variables will be interpreted as candidate resting thermo-autonomic correlates and never as standalone evidence of pain.

**Fig 3:**
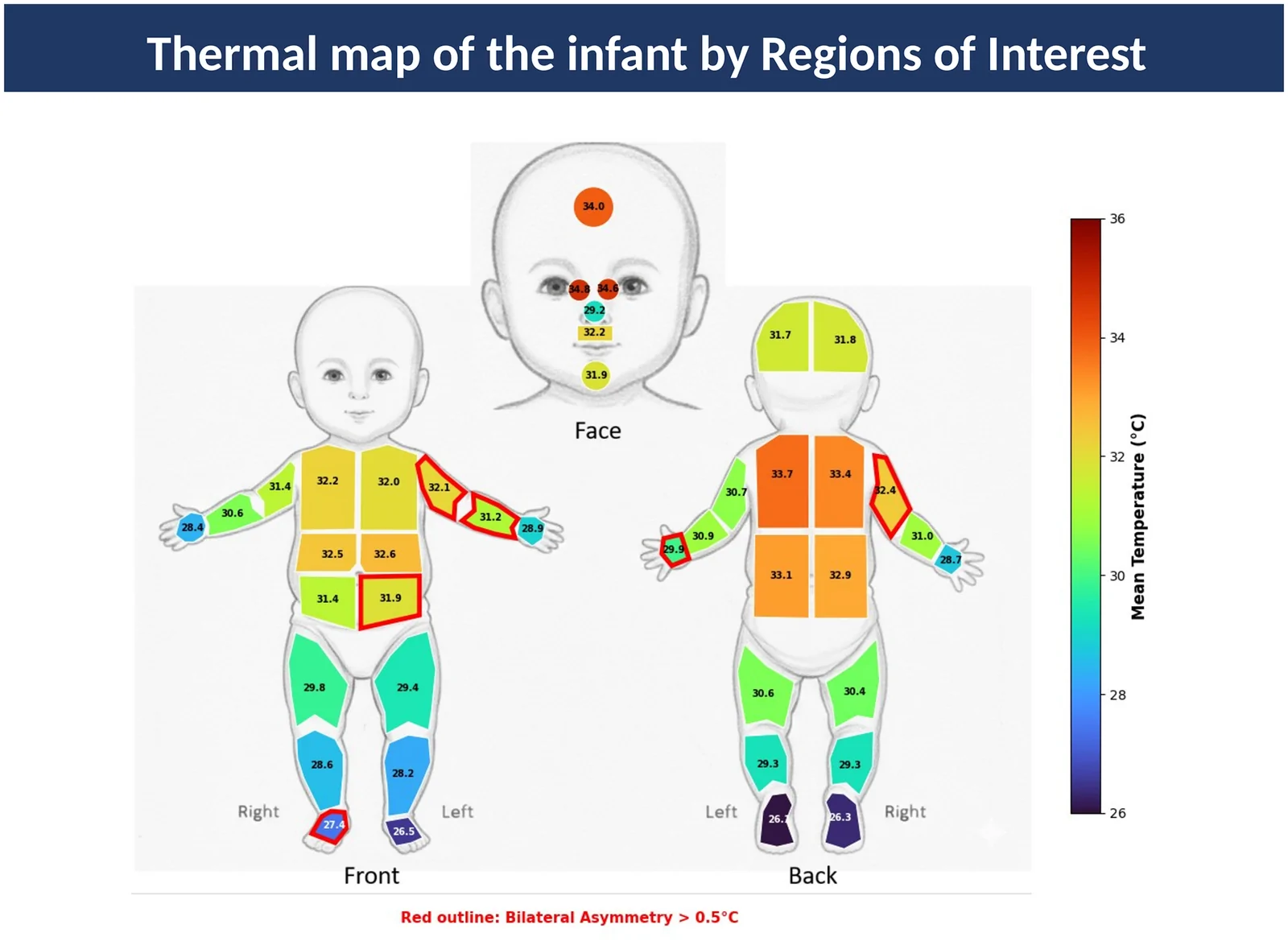
Thermal map of the infant by regions of interest (Theoretical data – figure presented for illustrative purposes only). Areas outlined in red indicate illustrative bilateral thermal asymmetries (> 0.5°C) between homologous left and right regions.

#### 2.4.3. Hair sampling and cortisol analysis.

Hair cortisol concentration is a biomarker of chronic stress reflecting prolonged activation of the hypothalamic-pituitary-adrenal axis. Hair sampling is a non-invasive and rapid method; samples are easy to store and stable over time [39]. Collection and analysis follow procedures described for infant populations [38,39].

Shortly after ITI, up to 50 mg of hair will be cut close to the scalp from the lower occipital region using round-tipped scissors and placed in a coded sterile tube. The target analytical mass is 20 mg [38]. Coded samples will be stored and transported at room temperature with shipment traceability to the Robert-Debré Hospital laboratory. The measurement of hair cortisol will be performed using high-performance liquid chromatography coupled with tandem mass spectrometry (HPLC-MS/MS). This technique combines chromatographic separation with specific detection via triple-quadrupole mass spectrometry, enabling sensitive and specific quantification of cortisol from small amounts of hair. This analytical method is the validated reference for the analysis of hair cortisol [38]. Samples will be destroyed after study completion in accordance with the laboratory procedures.

### 2.5. Motor assessment

The pain and stress assessment will be followed by a motor assessment combining the recording of general movements and the Bayley-4 test. This approach provides a functional indicator of early neurological development [79,80].

#### 2.5.1. Recording of general movements (GM Trust protocol).

The Prechtl General Movements Assessment (GMA) is a standardized, non-invasive method for evaluating spontaneous motor activity from birth to 5 months post-term age [79]. Based on the qualitative assessment of movement complexity, variability, and fluency, it is considered a very sensitive early indicator of neurological dysfunction [79,80,94]. Beyond its established predictive value for cerebral palsy, abnormal general movements have also been associated with minor neurodevelopmental impairments affecting motor, cognitive, behavioral, and executive functioning later in childhood [95–98].

Recordings will follow the GM Trust protocol [79]: infants will be supine, wearing a diaper, and filmed for 3–5 minutes during active wakefulness, without interaction, pacifier use, crying, or hiccups. At T1 and T2, conventional video and ITI will overlap for two minutes during the acclimatization period.

Eligible T1, T2, and guardian-recorded videos from days 15, 30, and 45 will be scored independently by two GM Trust-certified assessors (JP and MDC) blinded to cohort, EDIN, and ITI results. Agreement will be estimated separately for the age-appropriate score; disagreements will then be resolved by consensus, with adjudication if required.

Age-appropriate standardized scoring systems will be used. During the writhing movement period (i.e., for infants up to 8 weeks CA), motor performance will be assessed using the General Movement Optimality Score–Revised (GMOS-R), a standardized quantitative measure of movement quality ranging from 0 to 38 points, with higher scores indicating a more optimal motor repertoire [99]. During the fidgety movement period (i.e., for infants aged 9–20 weeks CA), motor performance will be assessed using the Motor Optimality Score–Revised (MOS-R), which evaluates fidgety movements, movement repertoire, posture, and movement character, yielding a total score ranging from 5 to 28 points [80]. Both instruments have demonstrated excellent inter-rater reliability and strong predictive validity for later neurodevelopmental outcomes [98]**.** For descriptive interpretation and according to published recommendations, a MOS-R score below 20, or a mildly reduced score accompanied by asymmetrical movement or postural patterns, will be considered indicative of increased neurodevelopmental risk and may warrant closer clinical follow-up [100]**.**

#### 2.5.2. Bayley-4 scales.

The Bayley Scales of Infant and Toddler Development, Fourth Edition (Bayley-4) [101], is a standardized assessment of cognitive, language, and motor development, widely used in children aged 16 days to 42 months [102]. Available evidence supports its validity and inter-rater reliability [83,84]. Five subtests (Cognitive, Receptive and Expressive Communication, Fine Motor, and Gross Motor) will be administered at T1 and T2 by a trained team member (IC, CP, JP or SBA). Sessions will be video-recorded and independently rescored by a second trained investigator (JP, SBA or JB), blinded to cohort and other outcomes; discrepancies will be resolved by consensus and, if necessary, adjudication. Items will be scored according to the Bayley-4 manual using a three-point system [101]: 0 = Not Present, 1 = Emerging, and 2 = Mastery. The final raw scores will be entered into Pearson’s Q-global scoring platform using only pseudonymized data. Age will be corrected for prematurity where applicable; raw, age-normed scaled, and Cognitive, Language, and Motor composite scores will be retained for analysis [101].

### 2.6. Statistical analyses

As no directly relevant effect-size estimate exists, 80 infants (40 per cohort) will be included to target at least 60 with evaluable paired T1 ITI–EDIN data. Assuming a two-sided α = 0.05, 60 analyzable infants provide approximately 80% power to detect |ρ| = 0.35 for one thermal parameter before multiplicity adjustment. This calculation describes sensitivity for an exploratory pilot study and is not confirmatory power for the full family of analyses.

Participant characteristics and data completeness will be summarized using appropriate descriptive statistics. Changes in crying status after baseline classification will be described and explored longitudinally.

The primary analysis will estimate T1 associations between a limited, prespecified family of ITI parameters and EDIN total scores using Spearman correlations with 95% bootstrap confidence intervals. The final set of thermal parameters will be specified in a statistical analysis plan finalized before database lock and outcome analysis and will define the hierarchy of mean ROI temperatures, bilateral asymmetries, core-to–peripheral gradients, and intra-facial gradients. Benjamini–Hochberg false-discovery-rate correction will be applied within each prespecified family; analyses remain exploratory because no validated primary thermal marker exists.

Longitudinal ITI and EDIN outcomes will be analyzed with mixed-effects models including baseline cohort, time, and cohort-by-time interaction, with random intercepts for participant and matched pair. Transformations or generalized mixed models will be considered if assumptions are not met.

Secondary analyses will investigate: (1) associations between thermal parameters, EDIN, and hair cortisol; (2) cohort differences in age-appropriate GMOS-R/MOS-R and Bayley-4 outcomes; and (3) associations between pain-related indicators and developmental outcomes. Depending on data distribution and study design, analyses will include Spearman correlations, Wilcoxon signed-rank tests for matched comparisons, regression models adjusted for clinically relevant covariates prespecified in the statistical analysis plan, and mixed-effects models.

Interpretation will emphasize effect sizes and 95% confidence intervals. Nominal two-sided *p* < 0.05 will be reported, with false-discovery-rate-adjusted *p* < 0.05 applied only within prespecified families of related comparisons.

The primary analysis will include evaluable paired T1 ITI–EDIN data, whereas longitudinal mixed-effects models will use all available repeated observations. The statistical analysis plan will define ITI evaluability, thresholds for sensitivity analyses, and plausible missing-data assumptions.

### 2.7. Blinding procedures

Several blinding procedures will be implemented to minimize measurement and interpretation bias. Clinical investigators who collect diaries, classify cohorts, and score EDIN (CP and IC) will not process ITI or rate General Movements. Investigators responsible for offline thermal image processing and General Movements ratings (JB, SBA, and JP, as applicable) will analyze coded files while blinded to cohort, crying measures, EDIN, cortisol, and other outcomes. Thermal image extraction will be performed independently by two trained investigators (JB and SBA). General Movements assessments will be rated independently by two certified assessors according to the General Movements Trust methodology (JP and MDC). Bayley-4 video recordings will be independently rescored by a trained investigator (JB, JP or SBA) who did not administer the corresponding session and who will be blinded to group allocation. Initial agreement will be quantified before consensus, with adjudication when required. The analysis dataset will be pseudonymized and analyzed under a statistical analysis plan finalized before database lock and examination of ITI–clinical associations.

### 2.8. Data monitoring and confidentiality

Data will be entered in a secure e-CRF under a unique study code with role-based access and an audit trail (S7 Appendix). Signed consent forms will be stored separately in locked cabinets at each study center. Digital data will be held in an HDS-certified environment in accordance with the GDPR and French requirements.

Sponsor monitoring will be risk-proportionate and will include a site initiation visit, early on-site monitoring after the first inclusions, and subsequent on-site or remote monitoring, including checks of informed consent, eligibility, and source-to-e-CRF consistency.

Legal guardian’s videos of GM and ITI recordings will be encrypted, coded, and stored separately from identifiers. Because participants may remain recognizable, recordings will be treated as identifiable personal data and restricted to authorized staff. Analytical datasets will exclude direct identifiers; essential documents and source data will be archived for 15 years under sponsor and RIPH2 requirements.

### 2.9. Ethics and dissemination

Legal guardians will receive clear written and oral information before enrollment and will provide written informed consent. They may withdraw their child from the study at any time, without providing a reason and without consequences for the child’s care. Families will receive a lay summary of aggregate results on the NEOTHERM website and non-diagnostic qualitative feedback when clinically relevant.

The study will follow the Declaration of Helsinki, applicable International Council for Harmonisation Guideline for Good Clinical Practice (ICH E6) principles, and French requirements. The protocol and data procedures were approved by the Centre Hospitalier de Saint-Denis scientific and ethics committee and received a favorable opinion from the Comité de protection des personnes Île-de-France II (ID-RCB 2025-A00767-42).

The protocol and subsequent study findings will be disseminated through peer-reviewed scientific publications, conference presentations, and lay materials intended for families and the general public.

## 3. Discussion

To our knowledge, NEOTHERM is among the first prospective studies to incorporate ITI into a multimodal investigation of suspected prolonged pain in infants with excessive crying. Growing evidence indicates that ITI can capture cutaneous thermal changes associated with autonomic responses to pain, stress, and affective arousal in other populations [40,59,61,70]. By evaluating a non-invasive, instrument-derived measure alongside behavioral assessment in early infancy [2,27,31], NEOTHERM may help characterize pain-related physiological responses during a critical period of neurodevelopment.

The main contribution of the study is methodological. It will examine whether standardized thermal parameters provide information complementary to behavioral signs assessed with EDIN, cumulative cortisol exposure, and early motor and developmental measures.

This multimodal approach acknowledges that prolonged pain is a complex phenomenon that cannot be adequately characterized by a single measure in non-verbal infants. Examining convergence and complementarity across these domains may clarify the relationships between pain-related behavior, autonomic regulation, cumulative cortisol exposure, and early development, without assuming that all measures reflect the same underlying construct.

Several methodological features strengthen the study. Thermal acquisition is standardized with respect to room conditions, infant positioning, acclimatization, and recording procedures [91,92]. Semi-automated processing, independent image analysis, and blinding to clinical data are intended to reduce observer-dependent variability and interpretation bias. These procedures should improve the consistency of thermal analyses and provide a framework that can be evaluated and refined in subsequent studies involving infants.

The prospective design, with two assessments spanning the expected peak and early decline of crying, will enable both between-cohort comparisons and examination of short-term within-infant changes. Although causal relationships cannot be inferred, this repeated-measures approach may provide insight into the evolving relationships among crying behavior, suspected prolonged pain, autonomic regulation, cortisol exposure, and early motor development.

As a pilot study, NEOTHERM will also provide estimates of effect sizes and within- and between-infant variability, together with information on data completeness, feasibility, and measurement reproducibility. These findings will help refine candidate thermal parameters and inform the design and sample-size calculations of subsequent confirmatory studies. The study will also document the practical implementation of repeated ITI acquisition and processing across neonatal and pediatric research settings.

Several limitations must nevertheless be considered. The sample size will limit statistical power and the precision of effect estimates. Because no validated thermal marker of prolonged pain is currently available, several predefined thermal parameters will be examined. This creates multiplicity and interpretive challenges despite false discovery rate correction. Moreover, the power calculation relates to an individual correlation and does not account for multiple testing. The analyses should therefore be regarded as exploratory and hypothesis-generating rather than as definitive validation of a specific thermal marker.

Excessive crying is itself a heterogeneous and age-dependent presentation that may reflect interacting biological, developmental, relational, and environmental factors [23,90]. Crying patterns may also change naturally between assessments, reducing between-cohort contrasts. NEOTHERM does not assume that excessive crying necessarily indicates prolonged pain; instead, it examines whether some infants display convergent behavioral, physiological, and developmental indicators consistent with suspected prolonged pain. Similarly, although EDIN was developed for prolonged pain assessment in preterm infants, its measurement properties are less well established in otherwise healthy young infants outside neonatal intensive care. EDIN scores may also be influenced by neurological maturation, clinical status, environmental context, and individual regulatory capacities [26,29]. In the absence of a gold-standard measure, associations between EDIN and ITI will indicate convergence between measures rather than pain specificity or diagnostic validity.

ITI is also sensitive to factors unrelated to pain, including ambient conditions, positioning, motor activity, feeding status, affective arousal, and individual differences in thermoregulation [91,92]. Standardized acquisition and blinded, semi-automated processing should reduce but cannot eliminate residual confounding and observer-dependent variability. Thermal patterns are therefore expected to reflect broader autonomic processes rather than pain alone, and ITI is being investigated as an adjunctive physiological measure rather than a standalone diagnostic test. Hair cortisol concentration is likewise non-specific and should be interpreted as an indicator of cumulative cortisol exposure, not as a direct measure of chronic stress. In early infancy, it may integrate prenatal, perinatal, and postnatal influences that cannot be attributed specifically to the period of excessive crying [32,37,38]. Limited hair availability may also result in missing or variable measurements [38].

Finally, the two assessment points restrict the study to short-term observations. General Movements and Bayley-4 findings should therefore be considered early indicators of developmental functioning rather than evidence of persistent neurodevelopmental outcomes. Longer follow-up and independent replication will be required to determine whether candidate thermal patterns are reproducible and prospectively associated with later development.

Overall, NEOTHERM may provide an important methodological contribution to infant pain research. If predefined thermal parameters show consistent relationships with behavioral and physiological indicators, the findings would support further evaluation of ITI as an adjunct to the assessment of suspected prolonged pain in non-verbal populations. Its non-contact nature, compatibility with repeated measurements, and potential for standardized, partially automated processing make ITI particularly suitable for pediatric research. More broadly, the study may help refine the multidimensional investigation of prolonged pain and inform future strategies for identifying infants who might benefit from early targeted support.

## 4. Conclusion

NEOTHERM will evaluate whether ITI-derived thermal markers complement behavioral assessment, cumulative cortisol exposure, and early motor and developmental measures in infants with excessive crying and suspected prolonged pain. By helping to render pain-related physiological responses more visible in infants, the study may support non-invasive, multimodal assessment and inform confirmatory research, early targeted support, and future evidence-informed pediatric recommendations.

## Supporting information

S1 Protocol Full NEOTHERM study protocol (Version 2, approved by the Ethics Committee on 18 March 2026). (PDF)

S2 Appendix Infant crying diary. (PDF)

S3 Appendix Information sheet and consent form. (PDF)

S4 Appendix Socio-demographic and medical questionnaire. (PDF)

S5 Table Whole-body regions of interest (ROIs) used for infrared thermal imaging analysis. ROI numbers correspond to paired right and left homologous anatomical regions. Expected physiological ΔT values represent the maximum expected temperature difference between homologous contralateral regions reported in the pediatric thermography literature. N/A indicates that no reference threshold was identified in the available literature.
(PDF)

S6 Table Facial regions of interest (ROIs) used for infrared thermal imaging analysis and expected physiological temperature variations. Facial ROIs were selected based on previous infant thermography studies and include regions in which thermal variations have been associated with autonomic and emotional responses. The central forehead (ROIv1) serves as the reference region for calculating intra-facial thermal gradients, allowing comparison of thermal patterns potentially associated with stress, discomfort, or prolonged pain.
(PDF)

S7 Appendix Case report forms. (PDF)

S8 Appendix SPIRIT checklist for NEOTHERM. (PDF)
