# Supplementary material for: Beyond crying: Protocol for a multimodal assessment of suspected prolonged pain in infants using infrared thermal imaging, behavioral assessment and physiological biomarkers (NEOTHERM)
Source: PLoS One. 2026 Nov 12;21(11):e0361344. doi: 10.1371/journal.pone.0361344 (PMC13759114; doi:10.1371/journal.pone.0361344)
Supplement: Supplementary file 1 [file pone.0361344.s001.pdf]

Assessment of Prolonged Pain in Infants with Excessive Crying: The Role of Thermal Imaging – NEOTHERM Pilot Study

Research Protocol Involving Human Subjects (RIPH2)

Version No. 2 dated

February 24, 2026 Project

Code: 0092_EPHE IDRCB

No.: 2025-A00767-42

Sponsor:<br>**Saint Denis Hospital** 2 rue du docteur Delafontaine 93200 Saint Denis<br>Tel: 01.42.35.61.40<br><br>***Principal Investigator:***<br>Dr. Alizée LORI<br>Head of the Neonatology Department Alizee.lori@ch-stdenis.fr	***Clinical Research Unit:* URC**<br>**GHT Plaine de France** Saint Denis Hospital 2 rue du Dr. Delafontaine 93200 Saint-Denis<br>Phone: 01.42.35.61.40 ext. 6962<br>Coordination.rechercheclinique@ghtpdfr.fr<br><br>**Project Coordinator:**<br>Coordination: **Ms. Stéphanie Cossec**<br>stephanie.cossec@ghtpdfr.fr
Partner:<br>EPHE – PSL University<br>Condorcet Campus, 14 Cours des Humanités, 93322 Aubervilliers<br>Phone: 01.88.12.02.99<br><br>***Principal Investigator:***<br>**Mr. François JOUEN,** Professor Emeritus at EPHE, HDR<br>francois.jouen@ephe.psl.eu	***Scientific Coordinator:***<br>**Dr. Joëlle PROVASI**<br>Associate Professor, HDR, Deputy Director of the Human and Artificial Cognition Laboratory (CHArt), joelle.provasi@ephe.psl.eu<br><br>***Project Leader:***<br>**Ms. Camille PERROLET**<br>Osteopath (DO), Ph.D. candidate in neuroscience (EPHE-PSL, CHArt Laboratory)<br>camille.perrolet@etu.ephe.psl.eu<br><br>***Other contributor:***<br>**Isabelle CARCHON**<br>Research Engineer, Ph.D. in Cognitive Psychology (CHArt) isabelle.carchon@ephe.psl.eu

Groupement Hospitalier de Territoire Saint-Denis Gonesse Plaine de France | École Pratique des Hautes Études | PSL | CHArt Cognitions Humaine et Artificielle

PROTOCOL SIGNATURE PAGE

<u>Study Title</u>: Assessment of Prolonged Pain in Infants with Excessive Crying: The Contribution of Thermal Imaging – NEOTHERM Pilot Study

<u>Protocol version and date</u>: Version 2, February 24, 2026

<u>Internal No.</u>: 0092_EPHE

This protocol was reviewed and approved on the date noted below

The parties agree to conduct the research in accordance with the protocol and the provisions applicable laws and regulations.

**<u>Sponsor's representative</u>: Mr. Jean PINSON**<br>General Manager<br>**Saint-Denis Hospital 2, rue du Dr. Delafontaine**<br>**93200 SAINT-DENIS**	**Date: 02/24/2026 Signature**<br>Directrice déléguéeà la Recherche<br>N. Albert<br>On behalf of the Chief Executive Officer and by delegation,<br>**Ms. Nathalie ALBERT**<br>Deputy Director of Research
**<u>Scientific Coordinator</u>: Dr. Joëlle Provasi**<br>CHArt Laboratory, EPHE-PSL<br>Condorcet Campus<br>14, Cours des Humanités<br>93 322 AUBERVILLIERS	**Date: 02/24/2026 Signature**
**<u>Project Leader</u>:**<br>**Ms. Camille PERROLET**<br>Osteopath, DO,	**Date: 02/24/2026**<br>**Signature**

Ph.D. candidate in neuroscience
(EPHE-PSL, CHArt Laboratory)

Study Abstract

Research Title	**Assessment of Prolonged Pain in Infants with Excessive Crying: The Contribution of Thermal Imaging - NEOTHERM**
**Advisor**	**Saint-Denis Hospital**
**Investigators**	**Principal Investigator**: Dr. Alizée LORI<br><br>**Scientific Coordinator**: Ms. Joëlle PROVASI<br><br>**Other Staff Members**: Ms. Isabelle CARCHON and Ms. Camille PERROLET
**Study Design**	A multicenter, prospective, observational, descriptive, and comparative pilot study with a longitudinal design.
**Study Population**	Infants aged 2 to 15 weeks (corrected age)
**Objectives**	**The primary objective of this study is to validate the use of near-infrared thermal imaging** (NIRTI) **for the assessment of prolonged pain in infants exhibiting excessive crying**, by comparing NIRTI results with those of a validated behavioral scale: the EDIN (Neonatal Pain and Discomfort Scale).<br><br>**The secondary objectives** will be:<br><br>**To analyze the agreement between measurements obtained from thermal imaging** (ITI), **the EDIN scale,** and **capillary cortisol levels**, used as a biological marker of prolonged stress.<br><br>**To describe and characterize spontaneous motor activity and**

	**psychomotor development** of infants exhibiting excessive crying through the analysis of gross motor movements and the Bayley Scales of Infant and Toddler Development (Bayley-4).<br><br>To explore **the associations between motor pattern characteristics and indicators of prolonged pain** (thermal imaging, EDIN, cortisol).
**Number of patients**	**80 infants**
**Eligibility criteria**	**Inclusion criteria:** post-term (PT) infants aged 2 to 6 weeks, born at ≥ 32 weeks' gestational age. No known neurological or gastrointestinal pathology. Written consent from legal guardians.<br><br>Division into 2 groups based on average daily crying duration (7-day parental diary):<br><br>"**Excessive** crying" **group**: > 3 h/day for ≥ 3 consecutive days (modified Wessel criteria) / meeting the Rome IV criteria (at least 2 out of 3 criteria).<br><br>"**Physiological** crying" **group**: < 2 h/day.<br><br>**Exclusion criteria for both groups**: confirmed brain lesions (ultrasound/MRI), major congenital or chromosomal abnormalities. Stunted growth and weight gain. Born before 32 weeks of gestation. Lack of consent from legal guardians. Subjects specifically protected by law.
**Outcome Measures**	**Primary outcome measures**: thermal images and behavioral assessment scale: Neonatal Pain and Discomfort Scale (EDIN).<br><br>**Secondary outcome measures**: capillary cortisol levels, analysis of general movements, Bayley Scales of Infant Development (Bayley-4)
**Study Protocol the study**	Participating infants from both groups will be recruited from 3 centers:<br><br>- Neonatology Department, *Dr. Alizée Lori, Saint-Denis General Hospital*

	- Perinatal Psychiatry Unit (consisting of Maison du Bébé, the Saint-Denis Parent-Child Care Unit, and the Perinatal Psychiatry Unit)*, Dr. Laure Quantin, Saint-Denis Hospital*<br>- Brantôme Perinatal and Early Childhood Medical-Psychological Center*, Dr. Anne-Sylvie Pelloux, 3rd arrondissement of Paris*<br>Interested parents will meet with the investigators **during an enrollment visit**: parental briefing, receipt and signing of the consent form, completion of two questionnaires (sociodemographic/medical data and impact of crying), and receipt of a crying diary. **Two data collection visits** will then be conducted two months apart, each organized in two parts:<br><br>**Pain assessment**: thermal imaging, EDIN scale, and capillary blood sample for cortisol level analysis.<br><br>**Motor assessment**: digital videos of general movements, Bayley-4 scales.<br><br>The thermal images and recordings of general movements will be analyzed by trained external observers who are blinded to the infants' assignment to treatment groups.
**Study duration**	**Duration of the enrollment period**: 20 months<br><br>**Duration of participation for each participant**: 2.5 months (inclusion visit + 2 follow-up visits conducted 2 months apart)<br>**Total study duration**: 26.5 months
**Research Sites**	Saint-Denis Hospital, Brantôme Mental Health Center in Paris,

TABLE OF CONTENTS

1. RESEARCH CONTEXT AND JUSTIFICATION 11

1.1 CONTEXT 11

1.1.1 CHRONIC PAIN IN INFANTS 11

Neurological Changes: Peripheral and Central Sensitization 11

Hormonal Changes—The Special Case of the First 100 Days 12

Potential consequences of prolonged pain in infants 12

1.1.2 EXCESSIVE CRYING IN INFANTS 13

Reflections on Excessive Crying in Infants: Definitions, Risk Factors, and Consequences 13

Explanatory Theories 13

The Role of Pain 14

1.1.3. ASSESSMENT OF CHRONIC PAIN IN INFANTS 14

Diagnostic Criteria 14

Assessment Scales 14

1.1.4. INFRARED THERMAL IMAGING (ITI) 15

1.2. RESEARCH QUESTIONS 16

1.3 : JUSTIFICATION FOR THE STUDY 17

2. OBJECTIVES 17

2.1. MAIN OBJECTIVE 17

2.2. SECONDARY OBJECTIVES 17

3. HYPOTHESES AND PROSPECTS 18

3.1. HYPOTHESES 18

3.2. OUTLOOK 18

4. RESEARCH DESIGN 19

4.1. EVALUATION CRITERIA 19

4.1.1. KEY EVALUATION CRITERIA 19

Infrared Thermal Imaging (ITI) 19

EDIN Behavioral Scale (Appendix 1) 19

4.1.2. SECONDARY EVALUATION CRITERIA 20

Capillary cortisol levels.....20
Analysis of general movements.....20
Bayley-4 Scales of Infant and Toddler Development (Appendix 3).....21
4.2. METHODOLOGY.....21
4.2.1 TYPE OF STUDY.....21
4.2.2 STUDY TIMELINE.....22
4.2.3. STUDY PLAN AND PROCEDURE.....22
Participating Centers.....23
Recruitment Procedure and Inclusion.....23
Appointment 1 – Initial visit (T1). Infant between 2 and 6 weeks post-term (corrected age).....26
Appointment 2 – Final visit (T2): Infants between 10 and 14 weeks post-term (corrected age).....28
4.2. STUDY TERMINATION CRITERIA.....29
4.3. SUMMARY OF THE STUDY PROCEDURE.....29
5. SELECTION AND EXCLUSION OF PARTICIPANTS FROM THE RESEARCH.....30
5.1. STUDY POPULATION.....30
5.2. INCLUSION CRITERIA.....30
5.3. EXCLUSION CRITERIA.....31
5.4. MATCHING CRITERIA.....31
5.5. SAMPLE SIZE.....32
6. DESCRIPTION OF DATA TO BE COLLECTED.....32
6.2. PRINCIPAL EVALUATION CRITERIA.....32
6.3. SECONDARY EVALUATION CRITERIA.....38
6.4. OTHER DATA COLLECTED.....39
7. STATISTICAL ANALYSES.....40
7.1. MAIN OBJECTIVE: ITI–EDIN ALIGNMENT.....41
7.3. SECONDARY OBJECTIVES.....42
7.3.1. ASSOCIATION BETWEEN ITI, EDIN, AND HAIR CORTISOL.....42
7.3.2. COMPARISON OF PSYCHOMOTOR DEVELOPMENT BETWEEN GROUPS 42
7.3.3. ASSOCIATIONS BETWEEN CHRONIC PAIN AND MOTOR PATTERNS ...42
7.3.4. SIGNIFICANCE THRESHOLDS AND INTERPRETATION.....43

8. DATA PROCESSING AND STORAGE OF DOCUMENTS AND RESEARCH DATA 43
8.1. ACCESS TO DATA 43
8.2. SOURCE DOCUMENTS 44
8.3. DATA CONFIDENTIALITY 44
8.4. DATA MANAGEMENT 45
8.5. DATA CIRCUIT AND SECURITY 45
8.6. PATIENT INFORMATION AND CONSENT 48
8.7. FINAL RESEARCH REPORT 48
8.8. CNIL 48
8.9. ARCHIVING 49
8.10. INSURANCE 49

9. ETHICAL CONSIDERATIONS 50

10. PUBLICATION GUIDELINES 50

11. BIBLIOGRAPHY 51

12. APPENDICES 58

APPENDIX 1: NEWBORN PAIN AND DISCOMFORT SCALE (EDIN) 58
APPENDIX 2: EVALUATION OF GENERAL MOVEMENTS FROM 0 TO 2 MONTHS 59
APPENDIX 2: EVALUATION OF GENERAL MOVEMENTS AT 3–5 MONTHS 60
APPENDIX 3: PSYCHOMOTOR DEVELOPMENT ASSESSMENT SCALE BAYLEY-4 61
APPENDIX 4: CRYING LOG – 7 DAYS 90
APPENDIX 5 – QUESTIONNAIRE A: SOCIO-DEMOGRAPHIC AND MEDICAL DATA 99

- CHILD 99
- MOTHER 99
- FATHER 99

APPENDIX 6: COLIQ® QUESTIONNAIRE 102
CRYING AND DISCOMFORT IN INFANTS 102

13. GENERAL INFORMATION 102

QUESTIONNAIRE STRUCTURE 102
OVERVIEW 102
SECTION 1: PATIENT INFORMATION 102
MODULE A: INFANT SYMPTOMS (BABY SCORE) 103
DIMENSION 1: QUANTITATIVE DESCRIPTION OF CRYING 103
DIMENSION 2: QUALITATIVE DESCRIPTION OF CRYING 103
DIMENSION 3: ASSOCIATED SYMPTOMS 104
DIMENSION 4: PAIN AND GASTROINTESTINAL DISCOMFORT 104
DIMENSION 5: INFANT BEHAVIOR 104
MODULE B: IMPACT ON PARENTS (PARENT SCORE) 105
DIMENSION 1: ACTIONS AND SOLUTIONS 105
DIMENSION 2: PSYCHOLOGICAL IMPACT 105
DIMENSION 3: IMPACT ON MARRIAGE 106
DIMENSION 4: IMPACT ON GENERAL WELL-BEING AND DAILY LIFE 106
DIMENSION 6: OVERALL IMPACT 107
SECTION 2: SCORE CALCULATION (TO BE COMPLETED BY THE INVESTIGATOR) 107
CALCULATED SCORES 107
SECTION 3: INVESTIGATOR'S OBSERVATIONS 107
SECTION 4: VALIDATION 108
APPENDIX 108
SCIENTIFIC JUSTIFICATION 108
ETHICAL CONSIDERATIONS 108
USE OF DATA 108
INTERPRETATION OF RESULTS 109
MILD SEVERITY GROUP: COLIQ SCORE > 70 109
MODERATE SEVERITY GROUP: COLIC SCORE BETWEEN 50 AND 70 109
HIGH SEVERITY GROUP: COLIQ SCORE < 50 109

APPENDIX 7: INSTRUCTIONS FOR PARENTS ON FILMING THE MOVEMENTS GENERAL 110

APPENDIX 8 – INFRARED THERMAL IMAGING - ITI 112

155

1. CONTEXT AND JUSTIFICATION OF THE RESEARCH

1.1 BACKGROUND

It is now well established that newborns, including preterm infants, possess the neurobiological capabilities necessary for pain perception. However, due to the immaturity of the nociceptive system, the mechanisms of this perception differ significantly from those observed in older children or adults (1,2). Although frequently underdiagnosed, chronic pain is present as early as the neonatal period and can have deleterious consequences on the child's somatic, neurological, and psychological development (3,4). Prolonged or repeated exposure to untreated pain is likely to induce lasting neurobiological changes, altering subsequent pain processing mechanisms (5).

While acute pain—particularly that associated with medical care—has been extensively documented, chronic neonatal pain remains under-researched, largely due to the lack of consensus diagnostic criteria and validated, objective, and reliable assessment tools (4,5). Among the suggestive clinical manifestations, excessive crying—which is often dismissed as trivial—could serve as a behavioral marker of chronic pain or prolonged stress (6). This type of symptomatology has significant repercussions on the quality of life of the child and those around him or her, as well as on the child's long-term neurocognitive development (7,8).

1.1.1 CHRONIC PAIN IN INFANTS

Neurophysiological data suggest that newborns experience pain consciously, in a more intense and diffuse manner than adults or older children, while being unable to modulate it effectively until the age of three months, due to the immaturity of their inhibitory system (1–4)**.**

Exploring chronic pain in infants presents various challenges, the first of which concerns its definition. In adults or older children, chronic pain is characterized by its duration—lasting longer than three months—and by alterations in pain processing that cause hyperalgesia (= increased sensitivity to pain) and allodynia (= non-painful stimulation perceived as pain). This definition requires adjustments to apply to infants. In 2017 (5), Anand proposed that pain persisting for more than 7 days be considered chronic in newborns.

Neurological changes: peripheral and central sensitization

The nociceptive system in infants exhibits a high degree of neuroplasticity, making them particularly sensitive to early environmental influences. In the event of repeated or prolonged painful stimuli, structural and functional changes in the nervous system can occur at both the peripheral and central levels. These reorganizations, known as **peripheral sensitization** and **central sensitization**, permanently alter the mechanisms of pain modulation and processing. They can lead to impaired pain processing or even chronic pain, reflecting

a persistent disruption of sensory circuits and neuroendocrine regulation. (3,6,7).

Hormonal Changes—The Special Case of the First 100 Days

In response to stress, two neurohormonal systems interact in a coordinated manner: the autonomic nervous system (ANS), via its sympathetic branch, and the hypothalamic-pituitary-adrenal (HPA) axis. Activation of the ANS induces a hemodynamic redistribution that favors vital organs (brain, muscles) at the expense of digestive and excretory functions. During prolonged stress, the HPA axis stimulates the secretion of cortisol, a glucocorticoid with major metabolic effects: mobilization of energy reserves (carbohydrates, lipids, proteins) and modulation of immune and inflammatory responses (8).

However, chronic stimulation of this axis, particularly in infants, can lead to persistent hypercortisolism with multiple consequences: digestive disorders, slowed growth, and neurodevelopmental impairments (attention, emotional, and social disorders) (8,9), particularly during the first 100 days of life—a period of heightened vulnerability due to high neuroplasticity (4,10). In preterm infants, this sensitive period is prolonged, exposing them to a greater risk of the deleterious effects of early stress, particularly through lasting alterations in HHS axis regulation and increased susceptibility to atypical developmental trajectories.

Chronic pain in infants can develop within a few days and be accompanied by changes in the nervous system (peripheral and central sensitization), leading to alterations in pain processing (hyperalgesia and allodynia) and stress. The pain signal may persist in the absence of injury or pathology (5,10)**.**

Potential Consequences of Prolonged Pain in Infants

In infants, exposure to pain induces physiological and endocrine responses characteristic of stress system activation: tachypnea, tachycardia, elevated blood pressure, as well as increased secretion of adrenaline and cortisol (4). At the same time, behavioral responses such as crying, agitation, grimacing, muscle tension, or, conversely, psychomotor atonia may be observed. These manifestations constitute the primary criteria for assessing pain in infants. Prolonged or repeated pain at an early age is likely to lead to behavioral disorders, including anxiety or withdrawal syndrome, as well as regulatory disorders (sleep disturbances, feeding difficulties, excessive crying) (3,11).

In the long term, persistent pain during the neonatal period is correlated with lasting alterations in stress and pain regulation systems, with major repercussions on physical and mental health. It is associated with increased vulnerability to neurocognitive disorders (delays in language and intellectual development), attention and behavioral disorders (hypervigilance, catastrophizing), sensory abnormalities (tactile hypo- or hyper-reactivity), and a high risk of chronic pain in adulthood (3,12). Furthermore, long-term somatic effects have been documented, including a

an increased prevalence of cardiovascular diseases (high blood pressure, diabetes, obesity) and impaired immune function ((13).

Current research on chronic pain highlights the complex interactions between biological, psychological, and social factors, leading to challenges in pain management, including in infants (13)**. Despite its negative impact on development, chronic pain in infants remains largely underestimated and inadequately managed due to the difficulties associated with its assessment.**

1.1.2 EXCESSIVE CRYING IN INFANTS

Reflections on Excessive Crying in Infants: Definitions, Risk Factors, and Consequences

Excessive crying in infants (also known as infant "colic") is defined, according to the Rome IV criteria, as prolonged episodes of inconsolable and unpredictable crying with no identifiable medical cause in an otherwise healthy infant (14,15). It is frequently associated with other regulatory disorders, particularly those related to sleep and feeding (16). Although crying is not a specific indicator of pain, it reflects a need or a state of discomfort. Data from international multicenter studies indicate a typical course: an increase in crying time up to six weeks of age, followed by a gradual decrease by 10 to 12 weeks, despite significant interindividual and cultural variations (20,21). Crying is classified as excessive when it exceeds 3 hours per day for 3 consecutive days during the week preceding the evaluation (modified Weissel criteria). This condition affects approximately 25% of infants between 3 and 6 weeks of age and is the most common reason for pediatric consultations during the first few months (17%) (17). A medical cause is identified in only 5% of cases (17).

The consequences can be significant, particularly from a psychosocial perspective: increased risk of parental depression, child maltreatment, premature cessation of breastfeeding, and cognitive and behavioral developmental disorders, especially when this crying persists or is associated with other regulatory disorders (15,17). Excessive crying has also been linked to delays in motor development and fine motor skills at 18 months and 5 years of age (18,19). Despite its prevalence, only three countries (the United Kingdom, Ireland, and the United States) have issued official recommendations, which are contradictory and poorly aligned with the available scientific evidence (17). The lack of consensus exacerbates parental anxiety and leads to inappropriate treatments (13).

The identified risk factors are multifactorial: genetic, neurodevelopmental, and environmental, including prenatal stress and obstetric complications (14,16).

Explanatory Theories

Several theories have been proposed to explain this crying. Some authors consider it to be a functional digestive disorder (hence the inappropriate term "infant colic") (17), while others view it as an extreme manifestation of immaturity

physiological neurological changes in infants during the first weeks of life, not directly related to pain (20,21).

The most plausible hypothesis at present views excessive crying as a behavioral manifestation of chronic stress, which activates the hypothalamic-pituitary-adrenal axis in the context of hypersensitivity to stress typical of the first three months of life
(8). In this context, excessive crying would serve as a biomarker of chronic stress, involving a persistent elevation in cortisol levels with systemic effects: neurological (hypervigilance, sleep disturbances), digestive disturbances (altered microbiota, slowed motility and absorption), musculoskeletal and dermatological repercussions, and so on. In vulnerable infants, this prolonged activation of the corticotropin axis could lead to a lasting alteration in future stress responses, due to the particularly marked neuroplasticity during the first 100 days of life (8,10). Early intervention is therefore crucial to limit the long-term deleterious effects of this hyperexcitability.

The Role of Pain

The exact causes of excessive crying remain to be determined, and the role of pain is difficult to assess. Some authors argue that this crying should not be considered a manifestation of pain, given the absence of an identifiable pathology in the majority of cases. However, this point is crucial, as infants are particularly vulnerable to the modulatory effects of painful stimuli (11).

1.1.3. ASSESSMENT OF CHRONIC PAIN IN INFANTS

Diagnostic Criteria

In infants, particularly in preterm newborns, chronic pain is difficult to identify due to the inability to verbalize pain and the subtlety of clinical signs. The most telling behavioral indicators include specific facial expressions, crying, agitation, or, conversely, psychomotor atonia, which may reflect a state of behavioral stupor (22). These manifestations are all the more difficult to interpret because they can be confused with other states of stress or discomfort.

Assessment Scales

Assessing pain in infants remains a challenge and relies primarily on the observation of behavioral signs—the most significant of which are facial expressions (23)—as well as on the analysis of physiological biomarkers related to the autonomic manifestations of stress (24,25). Capillary cortisol, a recognized biomarker of chronic stress, has proven particularly useful in this context (26).

Despite the development of numerous pain scales, there is no consensus on any single one, and very few have been validated for assessing chronic pain in infants (5,27). Multimodal scales (CONFORT NEO, N PASS), which combine physiological and behavioral criteria, are the most accurate. However, they require specialized equipment and are not suitable for use in outpatient pediatric settings.

Behavioral scales, such as the Neonatal Pain and Discomfort Scale (EDIN), are easier for clinicians to use. The EDIN allows for the qualitative and quantitative analysis of the main behavioral indicators of pain: crying, facial expressions, movements, sleep, and social interaction (5,28).

Furthermore, the rise of digital health offers promising prospects. The use of artificial intelligence, applied to automated facial expression recognition, and infrared thermal imaging (ITI), enables objective, continuous, and noninvasive pain assessment (29–31). These innovative tools could significantly improve care in neonatology and pediatrics, particularly in the management of vulnerable infants.

Chronic pain in infants remains underdiagnosed, largely due to the lack of validated specific tools and the complexity of its clinical presentation. An integrated approach, combining rigorous clinical observation, biomarkers, and technological tools, appears to be the most effective strategy for early detection and appropriate management. It is essential for clinicians and researchers to be able to identify and assess prolonged pain in infants early on in order to prevent its potential consequences.

1.1.4. INFRARED THERMAL IMAGING (ITI)

Infrared thermal imaging (ITI) is a noninvasive, nonionizing, and contactless method for measuring skin temperature in real time based on the body's natural infrared emissions. It thus accurately reflects the activity of the autonomic nervous system (ANS), which is involved in regulating peripheral circulation and in the response to stress and pain (23,32,33).

Cutaneous blood flow, which determines the skin's surface temperature, is controlled by the ANS. ITI allows for the dynamic recording of variations in this flow. Studies have confirmed a linear correlation between data from ITI and those obtained via laser Doppler imaging, reinforcing the validity of this method in the assessment of microcirculation (34). Under normal physiological conditions, the body's thermal distribution is symmetrical. Any significant asymmetry may indicate a metabolic, hemodynamic, or neurological impairment of thermoregulation (35,36). Thus, ITI is now recognized as a tool for analyzing cutaneous vasomotor autonomic activity (37).

Clinically, ITI is used in various medical fields: cardiology, endocrinology, neurology, oncology, orthopedics, and pediatrics (32). In neonatology, its integration into smart incubators has been explored for the automated monitoring of vital signs (33,38), and its application has been extended to the analysis of stress in infants based on facial thermal variations (39–42). It has also proven useful in diagnosing conditions such as necrotizing enterocolitis (43,44).

ITIs and Pain

Body Regions of Interest (Body)

ITI makes it possible to objectively identify functional abnormalities not visible on conventional anatomical imaging. It has thus been used to detect nociplastic and neuropathic pain in adults (complex pain syndromes, myofascial pain syndrome, sciatica, etc.) (45–48), children (49), and animals (50). By analyzing the thermal symmetry of regions of interest (ROIs), particularly under conditions of pain, it can detect significant temperature differences—often greater than 0.5 °C—that indicate autonomic dysregulation (35,36,51,52).

ROIs are defined based on anatomical vascular territories and/or the patient's clinical history (35,38). ITI has proven to be more sensitive than other techniques, such as MRI or bone scintigraphy, for the diagnosis of complex regional pain syndromes (CRPS) (52), and more effective than galvanic skin response tests for detecting neuropathic pain (48,53). Skin temperature has also been correlated with pain intensity in certain conditions such as lumbar disc herniation and myofascial pain syndrome (37,54).

Recent technological advances have significantly improved the spatial and thermal resolution of ITI (up to 0.08 °C and 1 mm²), enabling detailed, real-time image analysis (55). This method is therefore promising for diagnosis, treatment monitoring, and understanding the mechanisms of pain (56).

Facial Regions of Interest (ROI)

The face is a key region for assessing autonomic responses. Here, ITI allows for the measurement of thermal variations induced by activation of the ANS, which regulates functions such as heart rate, respiration, peripheral perfusion, and sweating (32,57). The areas most sensitive to emotional and nociceptive changes are the tip of the nose, the perioral region, the periorbital regions, and the chin (41,42,57).

Thermal changes in these regions are associated with emotional states, pain, or threatening situations. They may result from peripheral vasoconstriction, muscle activation, or changes in heart rate (39,40). ITI therefore allows for a noninvasive analysis of these indicators without disrupting the environment (40).

A study conducted by Czaplik et al. (2017) (56) established a thermal pain score (TAPI—*Thermal Associated Pain Intensity*) correlated with the visual analog scale (VAS) in adults who had undergone surgery. This score demonstrated a sensitivity greater than 0.75 for pain detection. The ITI thus emerges as a potential tool for the objective assessment of pain, applicable in infants.

1.2. RESEARCH QUESTION

Despite advances in the understanding of neonatal pain, the underlying causes and mechanisms of excessive crying in infants, as well as their potential link to chronic pain, remain insufficiently elucidated. The lack of criteria

and the low sensitivity of current tools—whether behavioral or physiological—compromise the reliable assessment of chronic pain in infants.

Furthermore, the neurodevelopmental impacts—particularly motor impacts—of excessive crying remain largely unexplored, although some studies suggest an association with subsequent motor disorders (18,19,58). These gaps hinder the implementation of appropriate care and underscore the need to develop more objective and sensitive assessment tools, as well as to better document the functional consequences of these early-onset disorders.

1.3 RATIONALE FOR THE STUDY

This study aims to improve our understanding of chronic pain in infants with excessive crying by proposing a multimodal assessment. It combines the use of a behavioral scale (the EDIN), a biomarker of chronic stress (capillary cortisol levels), and infrared thermal imaging (ITI) for an objective and accurate assessment of pain. In addition, the study will examine the development of motor patterns in infants with excessive crying by analyzing their general movements and psychomotor development.

This innovative approach aims to better understand the underlying mechanisms of excessive crying and its potential links to motor development. This research will have direct clinical applications for the care of infants.

2. OBJECTIVES

2.1. PRIMARY OBJECTIVE

The main objective of this study is to validate the use of near-infrared thermal imaging (NIRTI) for the assessment of prolonged pain in infants exhibiting excessive crying, by comparing NIRTI results with those of a validated behavioral scale: the EDIN (Infant Pain and Discomfort Scale).

2.2. SECONDARY OBJECTIVES

The secondary objectives will be:

- **To analyze the concordance** between measurements obtained from **thermal imaging (ITI), the EDIN scale, and capillary cortisol levels**, used as a biological marker of prolonged stress.
- **To describe and characterize spontaneous motor activity and psychomotor development** in infants exhibiting excessive crying through the analysis of gross motor movements and the Bayley Scales of Infant and Toddler Development (Bayley-4).
- **To explore the associations between motor pattern characteristics and indicators of prolonged pain** (thermal imaging, EDIN, cortisol).

3. HYPOTHESES AND OUTLOOK

3.1. HYPOTHESES

Main Hypothesis

Infrared thermal imaging allows for the objective assessment of significant skin temperature changes associated with prolonged pain in infants with excessive crying, and the results obtained by ITI are consistent with those of the EDIN behavioral scale.

Secondary Hypotheses

Hypothesis 1: Variations in skin temperature measured by ITI are positively correlated with high EDIN scale scores and capillary cortisol levels, indicating a state of prolonged pain or stress.

Hypothesis 2: Infants with excessive crying exhibit specific motor patterns that may reflect a state of pain or chronic stress.

Hypothesis 3: Alterations in motor patterns are associated with higher levels of pain or stress, as assessed by ITI, EDIN, and capillary cortisol levels.

3.2. OUTLOOK

Contribution to basic research: data collection and analysis (thermal maps of infants, capillary cortisol levels, motor assessments) on a topic that has yet to be fully explored

Diagnostic aid:

- Proposing a multimodal assessment for evaluating chronic pain in infants that combines behavioral analysis, physiological analysis, and state-of-the-art imaging techniques.

- Validate an innovative, noninvasive tool for assessing pain in infants with direct clinical applications in outpatient and inpatient pediatric settings.

Clinical Implications:

- Identify risk factors for developmental delays to enable early intervention

- Improving pediatric care: early and informed screening for pain in infants, and the development of appropriate interventions.

4. RESEARCH DESIGN

4.1. EVALUATION CRITERIA

4.1.1. PRIMARY EVALUATION CRITERIA

Infrared Thermal Imaging (ITI)

Tool used: infrared thermal camera

Parameters measured: systematic skin temperature gradients of regions of interest on the body and face.

Method: Acquisition of thermal images under standardized conditions (controlled ambient temperature and humidity; child calm, without external stimulation). Image analysis by an external examiner blinded to EDIN scores and assignment group.

Thermographic images of the entire body and face will be divided into regions of interest based on their vascular distribution and data from the literature (35,36,59). Asymmetries in body and facial temperatures will be systematically assessed. The child will be filmed in the supine position and then in the prone position, lying on their back, for 2 minutes on each side. Measurements will be taken using a thermal camera (FLIR T5XX, T6XX, or OPTRIS 450i model). Skin temperature regulation is central to thermoregulation and is symmetrical and instantaneous under normal physiological conditions (36). Asymmetrical qualitative and quantitative changes in temperature indicate a metabolic, hemodynamic, or neurological alteration in thermoregulation in the affected region (49,52).

EDIN Behavioral Scale (Appendix 1)

Tool used: EDIN scale (Newborn Pain and Discomfort Scale).

Parameters measured: score based on 5 behavioral criteria: movements, facial expressions, sleep, comfort measures, and social interaction; rated on a scale of 0 to 5, with a total score ranging from 0 to 15.

Method: Scoring by a trained evaluator, blinded to the ITI.

The EDIN is a validated behavioral scale for the heteroassessment of pain in newborns and infants up to 9 months of age (5,28). The EDIN scale comprises 5 assessment criteria: facial expression, movements, sleep, interactive behavior, and comfort measures. Each item is scored on a 3-point scale based on symptom intensity; a score of 5 or higher indicates probable pain, and analgesia is recommended. It is easy to use, quick to administer (about 3 minutes), reliable (0.69), has good internal validity (0.92), and offers the advantage of assessing prolonged pain in infants (5).

4.1.2. SECONDARY EVALUATION CRITERIA

Hair Cortisol Levels

Method used: cortisol measurement in a hair sample (standardized biological analysis in a certified laboratory). The measurements will be performed by the Biochemistry and Immunology Laboratory at Robert Debré Hospital.

Measured parameters: hair cortisol level expressed in pg/mg of hair.

Method: Collection of a small strand of hair from the occipital region. Analysis by chromatography coupled with mass spectrometry. Hair cortisol analysis will be performed using high-performance liquid chromatography coupled with tandem mass spectrometry (HPLC-MS/MS), using an XEVO TQS spectrometer (Waters, triple quadrupole).

Cortisol is a steroid hormone involved in the stress response. Hair cortisol levels have been studied in newborns as a biomarker of chronic stress (26) and are associated with chronic pain in adults (60).

Capillary hair sampling is a noninvasive and rapid method; the samples are easy to store and remain stable over time (61,62). Protocols have been validated for the collection and analysis of capillary cortisol levels in preterm infants using hair samples weighing less than 2.5 mg (62).

Analysis of General Movements

Tool used: video recording of spontaneous movements.

General Movements Assessment (GMA) is a standardized, non-invasive method for the early evaluation of spontaneous motor development in infants. It is currently the best clinically available method for assessing the neurological status of infants under 6 months of age (63,64). It is based on a short video recording of an infant who is comfortably positioned, free to move, and not interacting with their environment, allowing for qualitative and quantitative analysis of general movements, posture, and spontaneous movements. In this study, this analysis is conducted for exploratory purposes only and is not diagnostic. Its objective is to examine whether infants exhibiting prolonged crying display specific characteristics in their early motor organization and to explore potential relationships between indicators of stress or prolonged pain and early motor development. The analysis will be conducted in accordance with the international methodological recommendations of the General Movements Trust, an organization responsible for standardizing and certifying evaluators trained in this method.

Parameters measured: frequency, amplitude, and quality of general movements; other spontaneous movements; posture. Analyses will be performed using standardized observation scales based on the child's age and movement characteristics: General Movement Optimal Score—Revised (GMOS-R) for preterm and term infants

up to 2 months, and the Motor Optimal Score (MOS) (Appendix 2), for movements between 3 and 5 months.

Method: The analysis will be conducted by two trained observers (GM Trust Basic and Advanced), who will be blinded to the assignment group, the EDIN score, and the ITI results.

Each infant's gross motor movements will be filmed and recorded according to the protocol described by Einspieler and his team (63). The infant will be filmed in the supine position, wearing only a diaper or onesie, during 2 to 5 minutes of active wakefulness (without crying, interaction, a pacifier, or hiccups). The assessment will be conducted by two external observers, trained in the analysis of gross motor skills and blinded to the assignment group, who will assign a GMOS-R or MOS score depending on the child's age.

<u>Bayley-4 Infant and Toddler Development Scales</u> (Appendix 3)

Tool used: The infant's overall development will be assessed using the Bayley Scales of Infant and Toddler Development – Fourth Edition (Bayley-IV), a validated reference tool for children aged 1 to 42 months (65).

Measured parameters: The assessment includes five subtests:

- COG: Cognitive
- CR: Receptive Communication
- CE: Expressive Communication
- FM: Fine Motor Skills
- GM: gross motor skills

Method: The assessment is conducted by one of the EPHE staff members (Ms. Camille Perrolet, Ms. Joëlle Provasi, or Ms. Isabelle Carchon)

Each item is scored according to the manual's rubric:

- 0 = failure,
- 1 = partial success or hesitant response,
- 2 = complete success (according to the specific criteria in the Bayley-IV manual).

Composite scores are calculated on the secure **Pearson Clinical** platform (GDPR-compliant hosting). Data transmitted to Pearson is pseudonymized (study ID only, without any personally identifiable information). Raw and aggregated (standardized) scores are incorporated into the study database.

4.2. METHODOLOGY

4.2.1 STUDY DESIGN

A multicenter, prospective, observational, descriptive, and comparative pilot study with a longitudinal design.

4.2.2. STUDY TIMELINE

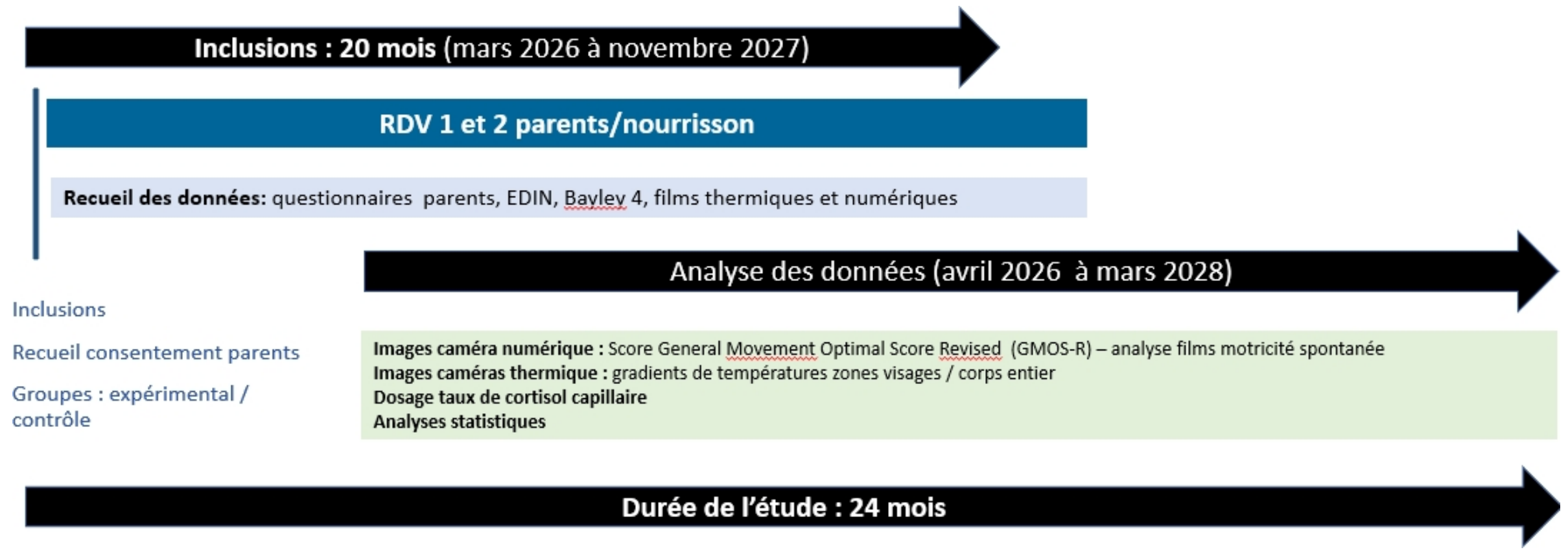

The study will last a total of 26.5 months, beginning in March or April 2026. The estimated enrollment period is 20 months.

This estimate is based on:

- The number of participating hospitals,
- The usual volume of patient visits in the relevant departments,
- The prevalence of excessive crying in the age group under study.

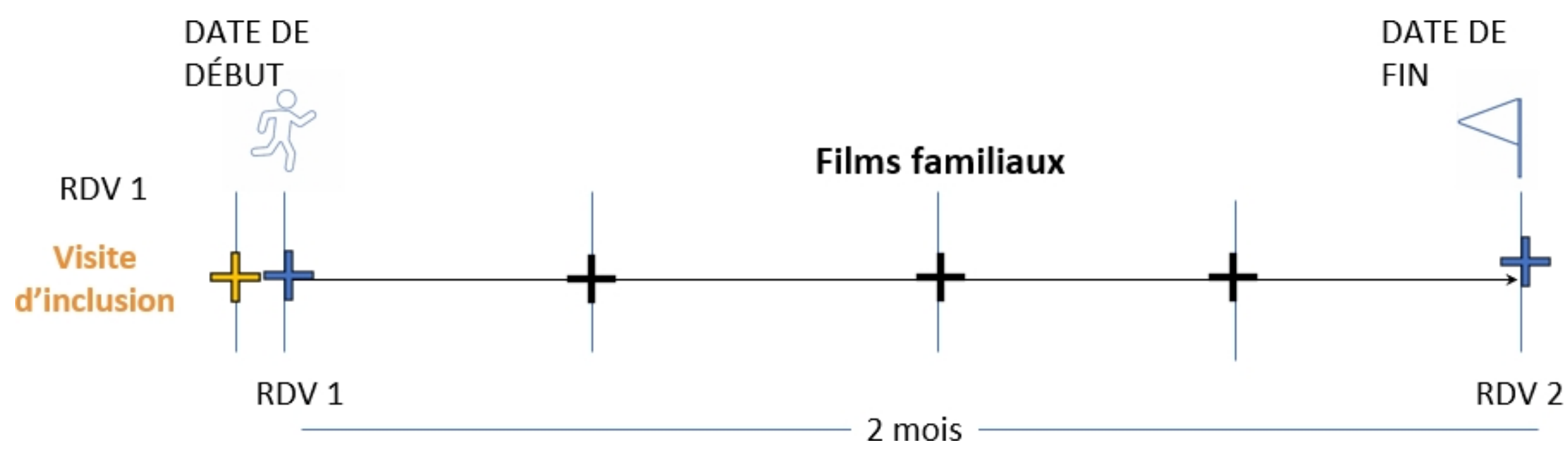

The duration of participation for each included infant is 2 and a half months.

4.2.3. STUDY DESIGN AND PROCEDURE

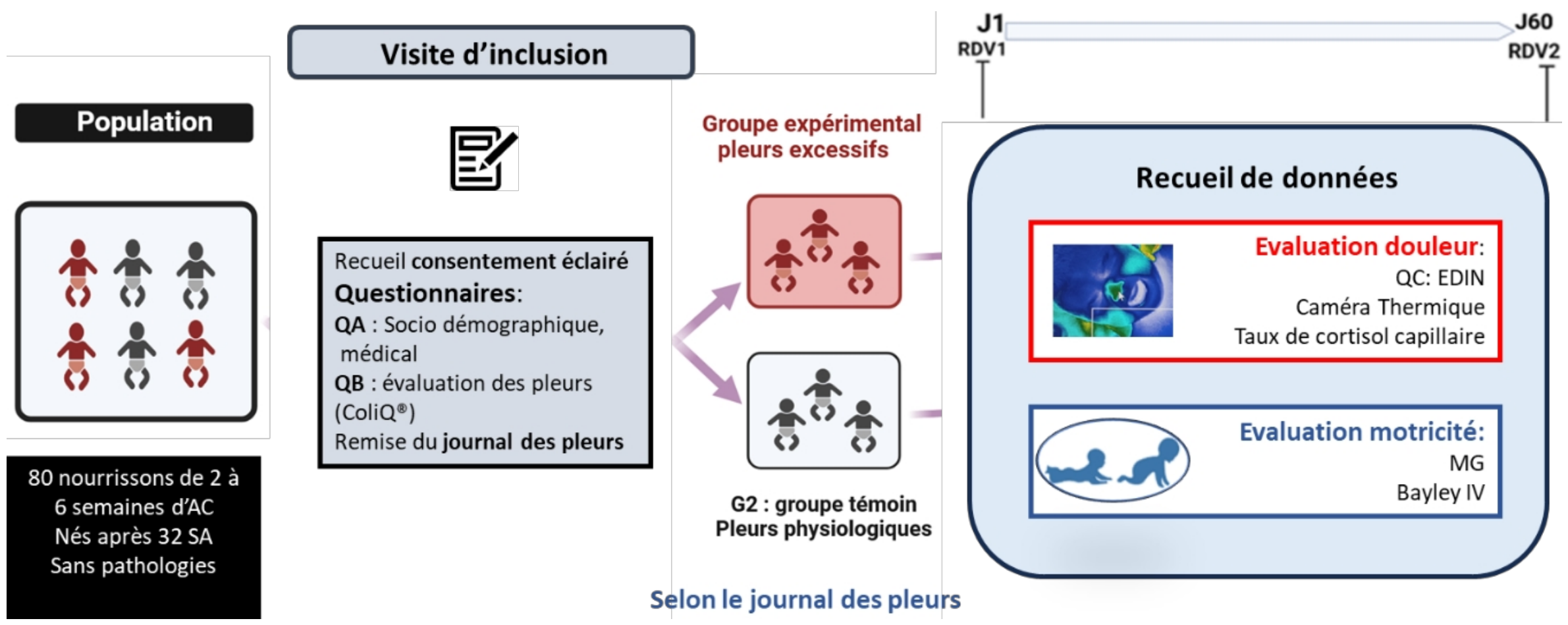

NEOTHERM is a prospective comparative observational study involving two parallel groups of infants defined a priori according to an exposure criterion, namely the daily duration of crying.

The infants are divided into:

- a group exposed to prolonged crying (> 3 hours per day according to the modified Wessel criteria and the Rome IV criteria),
- a group exhibiting physiological crying (< 2 hours per day).

Clinical, behavioral, and physiological data are collected prospectively at two standardized assessment time points

<u>Participating Centers</u>

- Neonatology Department, *Dr. Alizée Lori, CH Saint-Denis (Center No. 01)*
- Perinatal Psychiatry Unit (consisting of Maison du Bébé, the Saint-Denis Parent-Child Care Unit, and the Perinatal Psychiatry Unit), *Dr. Laure Quantin, Saint-Denis Hospital (Center No. 02)*
- Brantôme Perinatal and Early Childhood Medical-Psychological Center, *Dr. Anne-Sylvie Pelloux, 75003 Paris (Center No. 03)*

<u>Recruitment Procedure and Inclusion Criteria</u>

Children will be recruited in one of two ways:

a) Through the investigators

Recruitment will take place at participating hospitals. Potentially eligible infants will be identified by the medical teams in the relevant departments: an investigator will present the study and provide parents with the Information Sheet along with the Consent Form. The investigator will answer the

answer any questions parents may have and explain that they will be contacted by phone by one of the three EPHE representatives, whose names are listed at the top of the Information Sheet, to schedule a meeting—if they wish—to proceed with enrollment in the study.
During the phone call, Camille Perrolet, Isabelle Carchon, or Joëlle Provasi will review the details of the study and answer any questions the parents may have; if they still agree, an appointment will be scheduled for them.
During this first meeting, parents will be asked to sign a consent form: if they agree, this meeting will be classified as an inclusion visit.

Camille Perrolet, Isabelle Carchon, and Joëlle Provasi are bound by confidentiality regarding the identities and contact information of the families.

b) Through Posters

Information about the study will be disseminated via posters (Appendix 8), displayed in the waiting rooms of participating hospitals, as well as in community healthcare facilities—particularly maternal and child health services (PMI)—in coordination with the study sites.
This document briefly describes the study and includes a QR code linking to the study's website: Neotherm.fr (https://sites.google.com/view/neotherm-fr/accueil).

Interested parents may contact the research team (Ms. Camille Perrolet, Ms. Isabelle Carchon, or Ms. Joëlle Provasi) using the contact information provided to obtain additional information. They may also download the Information Sheet and the Consent Form, which are available online.

The study's website (Neotherm.fr) is designed to provide families with clear, consistent, and accessible information about the research. Access to the site will be strictly limited to individuals who have the link or QR code provided by the research team; it will not be indexed by search engines. It outlines the study's objectives, its procedures, the practical details of participation, and the research team's contact information. A contact form will allow parents who wish to do so to submit their contact information so that the research team can reach out to them. The data collected through this platform will be limited to what is strictly necessary, used solely to organize contact with interested families, and processed in accordance with current regulations regarding the protection of personal data (GDPR). The sole purpose of this platform is to improve the information provided to families and to facilitate communication with the research team.

During an initial phone call, parents will be able to ask questions and, if they wish, schedule an intake visit. This visit does not obligate participation. Camille Perrolet, Joëlle Provasi, and Isabelle Carchon will ensure that parents have received the information sheet before ending the call.

Parents will be asked to review this document at home so that they have the necessary time to consider their decision—**at least three days**—before deciding whether to participate.

During the inclusion visit at the study site, an authorized investigator will verify the inclusion criteria, review all information related to the study with the parents, and answer any questions they may have. The informed consent form will be signed only during this visit, after the parents confirm their understanding of the study.

The reflection period may be extended if the parents wish.

Regardless of the method of initial contact (a or b), providing information about the study, verifying eligibility criteria, and obtaining informed consent will be carried out by an authorized person listed on the delegation of duties form.

Enrollment and all visits specified in the protocol will be conducted exclusively at participating hospitals by Ms. Camille Perrolet, Ms. Isabelle Carchon, or Ms. Joëlle Provasi, under the supervision of the center's principal investigator.

The decision to participate or not to participate in the study will have no impact on the quality, nature, or continuity of the child's usual medical care.

<u>INCLUSION VISIT</u>

Prior to the enrollment visit, parents will have received initial information about the study from the department's healthcare teams or from the research team following an initial phone call or through the informational materials provided. The information sheet and consent form will have been given to them so that they can review them at home and **have the minimum reflection period of 3 days.**

The enrollment visit, conducted at the study hospital, aims to review all information related to the research with the parents or legal guardians, answer any questions they may have, and verify the infant's eligibility criteria.

After confirming that the parents fully understand the study and that the minimum reflection period has been observed, informed consent will be obtained by an authorized person listed on the delegation of duties form. If the parents wish to have more time to consider their decision, another enrollment visit will be scheduled.

Finally, once the signed consent form has been obtained, the researcher will ask the parents the questions from the following questionnaires orally:

Questionnaire A = sociodemographic data, pregnancy, birth (gestational age, mode of delivery, complications, measurements), maternal smoking, medication use during pregnancy, dietary habits, medical history, treatments received (Appendix 5). (5–10 min)

Questionnaire B = ColiQ (21): qualitative and quantitative assessment of crying and its impact on quality of life (Appendix 6) (10–15 min).

The date of the first appointment (Appointment 1) for measurements will be set by mutual agreement between the investigator and the parents.

At the end of the enrollment visit, **a crying diary** (Appendix 4) will be provided to the parents. The crying diary is to be completed daily by the parents for 7 consecutive days during the week preceding Appointment 1. It is used to record the infant's main behaviors (crying, restlessness, sleep, feeding, calm wakefulness) by time interval (63–65).

Data collection will take place during two appointments, each lasting one hour, spaced approximately two months apart. These visits will be conducted exclusively at participating hospitals by an authorized investigator listed on the delegation of duties.

<u>Appointment 1 – Initial visit (T1). Infant between 2 and 6 weeks post-term (corrected age)</u>

After collecting the crying diary and based on information provided by the parents, two groups of infants will be formed:

➢ **An experimental group**, **excessive crying**: consisting of infants aged 2 to 6
post-term (corrected age), with excessive crying (modified Wessel criteria: more than 3 hours of crying per day, for more than 3 consecutive days during the week preceding the evaluation), meeting at least 2 of the following 3 criteria: the infant exhibits prolonged crying episodes 1) with no identified cause, 2) that are inconsolable, 3) accompanied by associated signs: agitation and/or a flushed face and/or clenched fists and/or flexed legs (ROME IV criteria).

➢ **A control group, physiological crying**: consisting of infants aged 2 to 6 SPT (corrected age), with no history of excessive crying (< 2 hours of crying per day).

The distinction between the "excessive crying" and "physiological crying" groups is based on thresholds established in the literature (ROME IV and modified Wessel criteria)(16).

Infants with an intermediate crying duration (between 2 and 3 hours per day) will not be included in these groups in order to limit data overlap and preserve the internal validity of the statistical comparisons: in this case, the RDV1 visit will serve as the end-of-participation visit. No other data will be collected or used for the purposes of this study (data collected during the enrollment visit will not be used).

For infants assigned to the "experimental" and "control" groups, the RDV1 visit will then consist of two parts: a **pain assessment** and a **motor skills assessment**.

➢ **<u>1 Pain assessment</u>:**

The investigator will ask the parents whether the child was ill between the two visits and whether there have been any changes to any ongoing treatments (see e-CRF).

Questionnaire B: ColiQ

Questionnaire C: Neonatal Pain and Discomfort Scale (EDIN) (Appendix 1) The investigator will ask the parents the questions from the EDIN scale.

Before the experimental recordings, the infants will have a 5-minute acclimatization period during which the experimenter will explain the session procedure and measurement process to the accompanying adult and start the thermal camera recording.

Thermal camera recording (2 minutes): the child, undressed (wearing only a diaper), is positioned in the supine position and then in the prone position on the examination table.

In accordance with standard guidelines for infrared thermography research, the environment will be thermoneutral; procedures will be conducted under conditions of stable temperature (23° ± 1°) and humidity (50–60%), as measured by a thermostat.

Capillary hair sample collection: Capillary hair samples will be collected according to the protocol described by Nist et al., 2020 (62). A small amount of hair (≤50 mg, with 20 mg required for analysis) will be cut as close to the root as possible from the lower occipital region of the child's scalp. Each sample will be placed in a sterile Eppendorf tube, labeled with a unique code and the date of collection, then wrapped in a light-protective aluminum pouch and placed in a rigid container with absorbent padding. The samples will be stored at Delafontaine Hospital in Saint-Denis until they are transferred to Robert Debré Hospital.

➢ **2. Motor assessment**:

Digital video recording (5 minutes): According to the GM Trust protocol, the child will be filmed unclothed (wearing only a diaper) in a supine position, allowed to move freely, without interaction or a pacifier. The camera will be positioned above the child using a tripod.

Bayley Scales 4: The assessment will be conducted by the researchers (Ms. Camille Perrolet, Ms. Joëlle Provasi, and Ms. Isabelle Carchon), who are trained in the Bayley scales. The assessments will be filmed (duration: approximately 15 to 20 minutes) to be scored by the coordinating investigator and an external observer—a doctoral student or researcher from the CHARt laboratory—who will be listed on the delegation of duties form before the study begins.

If the infant cries or shows signs of discomfort, efforts will be made to bring the infant into a calm, alert state (by holding the infant close or feeding the infant).

At the end of Visit 1, parents will be asked to record home videos of their infant's general movements on Day 15, Day 30, and Day 45, and they will be given an instruction sheet for

the filming process and how the files will be delivered (via a USB drive), and a second crying log to be completed the week before the second visit.

Family Videos: Parents will be asked to record family videos of their child's general movements 15, 30, and 45 days after Appointment 1. Reminders via text message will be sent to them before each deadline from the study coordinators' work phones. Parents must film according to specific guidelines that will be explained to them verbally by the investigator at the end of Visit 1 and described in the document: "Instructions for Parents on Filming General Movements," which will be provided to them (Appendix 7): film their infant while the infant is in a calm, awake state, lying on their back on a firm surface, without interaction or a pacifier, while standing over the infant, for 3 minutes (66). The videos will then be transferred to a dedicated USB drive. The USB drives will be stored in a secure cabinet.

Crying Log: Parents must complete a second crying log, identical to the first, one week before the date of Appointment 2. A reminder via text message will be sent to them 10 days and 7 days before the scheduled date of Appointment 2.

<u>Appointment 2 – Final Visit (T2): Infants between 10 and 14 weeks post-term (corrected age)</u>

The investigator will collect the USB drive containing the video files from Days 15, 30, and 45 after Visit 1, the completed second crying diary, and will ask the parents whether the child has been ill between the two visits and whether there have been any changes to any ongoing treatments (see e-CRF).

The investigator will then administer the QB: ColiQ questionnaire.

The steps for Visit 2 will then be the same as for Visit 1.

➢ **<u>Pain assessment</u>**: EDIN, temperature recordings, and capillary blood sample.

➢ **<u>Motor assessment</u>**: Digital videos and the Bayley IV scale recorded on video.

Participant follow-up will be conducted at the hospital where enrollment took place, under the responsibility of the principal investigator at that center. No follow-up will be conducted outside of the participating hospitals.

The travel required for the study consists of one enrollment visit and two scheduled visits over a two-month period.

To the extent possible, the three scheduled visits will be coordinated with the routine follow-up of infants in the relevant departments.

In the event that participation requires a specific trip, the sponsor will cover the expenses incurred upon presentation of supporting documentation.

The practical details will be explained to families during the preliminary information session.

Site visits and data collection will be conducted by the investigators identified in the protocol and listed on the delegation of responsibilities form: Ms. Camille Perrolet, Ms. Joëlle Provasi, and Ms. Isabelle Carchon.

If additional investigators are involved during the study, they will be formally listed on the delegation of duties by the relevant principal investigator, and their updated CVs and BPC certificates will be provided in accordance with regulatory requirements.

<u>END OF PARTICIPATION (intermediate crying duration, between 2 and 3 hours per day)</u>

At the start of the first appointment (Appointment 1), if analysis of the crying log indicates that the child's daily crying duration does not correspond to either of the two study groups (i.e., neither less than 2 hours per day nor more than 3 hours per day), the child's participation in the study will end. In this case, no further data will be collected or used for research purposes, in accordance with the ethical requirements of the protocol.

To ensure that the parents' visit is still worthwhile, a personalized verbal summary of the observations from the crying diary will be provided to help shed light on the child's rhythm and behaviors. Advice tailored to the child's specific situation, rhythm, and behavior may be offered, and, if necessary, a referral to professional resources or appropriate follow-up care may be suggested.

No therapeutic interventions will be performed as part of the study.

4.2. RULES FOR WITHDRAWAL FROM THE STUDY

Parents may withdraw their child from the study at any time without having to provide a reason.

4.3. SUMMARY OF THE STUDY PROCEDURE

**Procedure Added for the Study**	**Inclusion Visit**	**Visit 1 (Appointment 1)**	**Visit 2 (Appointment 2)*[2]**
Information/Consent Signature	X		
Questionnaire A	X		
Questionnaire A (update)		X*[2]	X
Questionnaire B: ColiQ	X	X*[2]	X
Questionnaire C: EDIN		X*[2]	X

Compilation of the Crying Journal		X	X
Randomization		X*1	
Thermal Camera Recording		X*2	X
Capillary blood sample		X*2 *3	X*3
Digital camera recording		X*2	X
Bayley Scale IV		X*2	X
Collection of the USB drive containing the 3 home movies			X

*1: or end of study
*2: if randomized
*3: Samples will be sent to the Biochemistry-Hormonology Laboratory & INSERM NeuroDiderot Biological Resources Platform at Robert Debré University Hospital, 48 Boulevard Sérurier, 75935 Paris Cedex 19, France

5. SELECTION AND EXCLUSION OF PARTICIPANTS FROM THE RESEARCH

5.1. STUDY POPULATION

Infants will be included between **2 and 6 weeks post-term (PT) in corrected age** for the first evaluation (Visit 1) and re-evaluated **between 10 and 14 PT for the second (Visit 2)**. This decision is based on two main considerations:

The physiological progression of crying: crying duration gradually increases from birth to 6 weeks**,** which corresponds to the peak of infant crying**, as** classically described in the literature (16,67,68). Crying then decreases until 10 to 12 weeks of age. This period also corresponds to the gradual establishment of the endogenous pain inhibitory system, resulting in increased sensitivity to pain during the first 3 months of life

Spontaneous motor development: this same time window coincides with key periods for observing general movements (General Movements)**.** Writhing movements (WM)**,** typical of the first few weeks after term, are observable between birth and 8–9 weeks, while fidgety movements (FM) emerge between 10 and 14 weeks. (63,64,69)

The scheduled visits at 1 month and 3 months thus allow for a consistent longitudinal analysis of spontaneous motor development.

This schedule therefore optimizes the physiological relevance of the observations while minimizing the burden on infants and their parents.

5.2. INCLUSION CRITERIA

- Infants aged **2 to 6 weeks post-term (PT)** in corrected age

- born at ≥ 32 weeks' gestational age
- with no known neurological or gastrointestinal pathology
- whose legal guardians have provided written consent to participate.

The included infants will be divided into **two groups** based on the average daily duration of crying reported by parents during the week preceding the first assessment:

"Excessive crying" experimental group: crying time > 3 hours/day for ≥ 3 consecutive days (modified Wessel criteria), meeting at least 2 of the following 3 criteria: the infant exhibits prolonged crying episodes 1) for no apparent reason, 2) that are inconsolable, 3) accompanied by associated signs: agitation and/or a red face and/or clenched fists and/or flexed legs (Rome IV criteria for infant colic).

"Physiological crying" control group: crying duration < 2 hours/day.

Infants with crying time between 2 and 3 hours per day during the week preceding the evaluation will not be included in either group, in order to avoid classification bias and ensure a clear distinction between the two comparison groups.

5.3. EXCLUSION CRITERIA

Confirmed brain lesions (ultrasound/MRI). Major

congenital or chromosomal abnormalities. Stunted growth

and weight gain.

Born before 32 weeks of gestation.

Non-French-speaking holders of parental authority - Lack of consent from holders of parental authority.

Not enrolled in a social security program. Individuals

specifically protected by law.

5.4. MATCHING CRITERIA

As part of this observational study, participants in the control group will be matched with participants in the experimental group to minimize confounding factors related to variables that may influence the infant's crying behavior.

Matching will be performed according to the following criteria:

Corrected age at the time of assessment (± 1 week),

Child's sex (1:1 girl/boy matching when possible),

Gestational age (± 1 week of amenorrhea),

Feeding method (exclusive breastfeeding, mixed feeding, or formula feeding),

Mother's parity (primiparous/multiparous).

This strategy aims to create two comparable groups, limiting the potential influence of perinatal or environmental factors on the observed crying duration.

5.5. SAMPLE SIZE

This study is a **descriptive**, **observational pilot study**, the primary objective of which is to explore the clinical and behavioral characteristics of infants with excessive crying duration compared to infants with physiological crying duration.

In accordance with methodological recommendations for pilot studies, a **sample size of 35 to 45 subjects per group** is generally considered appropriate for testing the feasibility of the protocol, the relevance of the outcome measures, and the variability of the measurements (70,71).

We therefore selected a sample size of **40 infants per group**, for **a total of 80 participants**, allowing for an initial assessment of data variability and the preparation of a larger-scale study with a dedicated sample size calculation.

6. DESCRIPTION OF DATA TO BE COLLECTED

6.2. PRIMARY OUTCOME MEASURES

➢ **EDIN: Neonatal Pain and Discomfort Scale**

The EDIN scale consists of **5 items** (facial expression, movements, sleep, interaction with the environment, and comfort measures), each with 4 possible ratings ranging from 0 to 3. A detailed description is provided to assist with scoring.

The total score ranges from 0 to 15. The generally accepted treatment threshold is 5/15(28).

➢ **Procedure for Analyzing Thermographic Images**

Thermographic images will be captured using a **FLIR® XXYY series infrared camera** (resolution ≥ 320×240 pixels, thermal sensitivity < 0.05 °C, accuracy ±2 %). The camera will be positioned at a distance of 70 to 100 cm from the infant, perpendicular to the imaging plane, in passive mode (without an external infrared source).

Imaging conditions: stable ambient temperature: 23 ± 1 °C, relative humidity: 50–60 %, acclimatization time for the infant prior to recording: ≥ 10 minutes**.** Images will be captured of the infant in a calm, awake state, in the supine position (2 min) and then in the prone position (2 min), without crying during the main capture.

Calibration and settings: Before each session, the **camera is calibrated** using a blackbody reference (emissivity = 0.98). Skin emissivity is set to 0.98 for all measurements, in accordance with ISO 18434-1 recommendations (72).Settings for distance, humidity, and ambient temperature are entered into the data acquisition software.

Data extraction and preprocessing: Thermographic images will be exported in radiometric format (.jpg or .seq).
Analysis will be performed using validated infrared analysis software (e.g., FLIR Tools, IRT Analyzer, or ThermoHuman®, depending on compatibility).

Processing will include: filtering out artifacts (motion, reflections, saturated areas); manual segmentation of regions of interest (ROIs) based on predefined anatomical maps (body and face); automatic extraction of the average, minimum, and maximum temperatures for each ROI; inter-rater verification of ROI positions on a 10% sample of the images (expected intraclass correlation coefficient > 0.90).

Quality control and reproducibility: Each acquisition session will include a calibration image and an ambient temperature measurement.

The acquisition and analysis protocol follows the recommendations in the literature on thermal imaging research (35,59,72).

The baby’s body and face will be divided into different regions of interest (ROIs), which correspond to vascular territories as shown in the diagrams below.

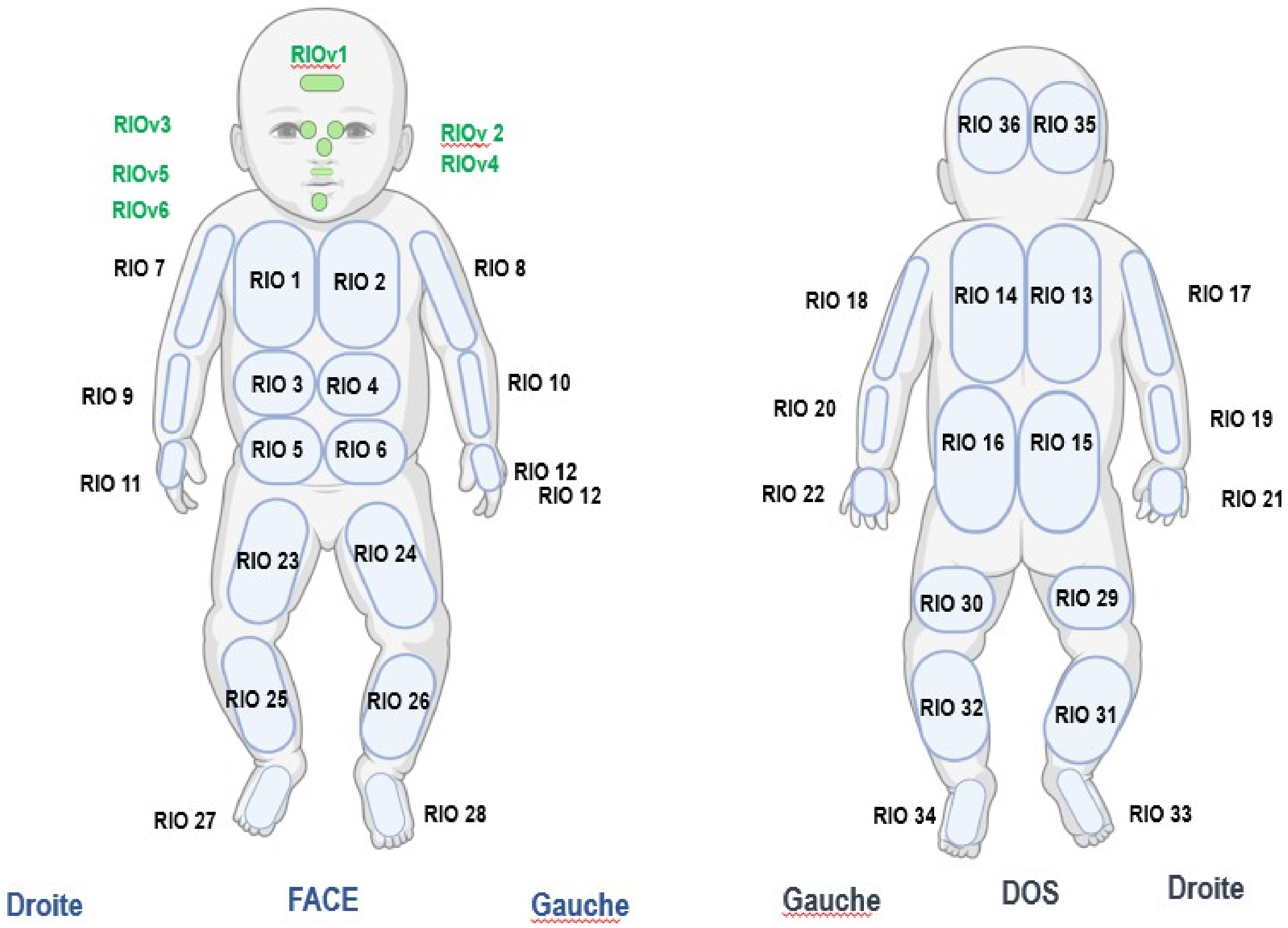

Figures 1. Body and face regions of interest for thermal imaging mapping

1) <u>Analysis of whole-body thermal images</u>:

The regions of interest are adapted from the adult model (35), adjusted to pediatric morphology based on the thermal distribution described by Kolosovas-Machuca and González (2011) (59). Infants' bodies will be divided into 36 symmetrical regions of interest on the front and back, numbered 1 through 36 (Table 1) (35). Average temperature variations per region will be calculated over a two-minute period. Right/left temperature asymmetries will be systematically assessed.

The analysis aims to identify **variations in skin temperature between central regions** (chest, abdomen) and peripheral **regions** (hands/feet) and between **the right and left sides,** in order to assess physiological thermal symmetry and pathological deviations (> 0.5 °C) potentially associated with pain or autonomic dysregulation.

Body temperature differences **(ΔT)** will be calculated using two approaches:

- **ΔT cp (center/periphery)**: difference between central and peripheral areas (e.g., chest vs. hand, abdomen vs. foot)
- **ΔT bilateral**: difference between homologous right and left regions

$$\Delta T_{bilateral} = | T_{right} - T_{left} |$$

$$\Delta T_{central\text{-}peripheral} = T_{central} - T_{peripheral}$$

ROI	Region	Plane	Side	Expected physiological ΔT
1	Upper chest	Anterior	Right	< 0.4 °C
2	Upper chest	Anterior	Left	< 0.4 °C
3	**Upper** abdomen	Anterior	Right	< 0.5 °C
4	**Upper** abdomen	Anterior	Left	< 0.5 °C
5	**Lower** abdomen	Anterior	Right	< 0.5 °C
6	**Lower** abdomen	Anterior	Left	< 0.5 °C
7	Proximal **arm**	Anterior	Right	< 0.4 °C
8	Proximal **arm**	Anterior	Left	< 0.4 °C
9	**Forearm**	Anterior	Right	< 0.5 °C
10	**Forearm**	Anterior	Left	< 0.5 °C

**11**	**Hand**	Anterior (palm)	Right	< 0.5 °C
**12**	**Hand**	Anterior	Left	< 0.5 °C
**13**	**Dorsal region**	Posterior	Right	
**14**	**Dorsal region**	Posterior	Left	
**15**	**Lumbar region**	Posterior	Right	
**16**	**Lumbar region**	Posterior	Left	
**17**	Proximal **arm**	Posterior	Right	< 0.4 °C
**18**	Proximal **arm**	Posterior	Left	< 0.4 °C
**19**	**Forearm**	Posterior	Right	< 0.5 °C
**20**	**Forearm**	Posterior	Left	< 0.5 °C
**21**	**Hand**	Posterior (back)	Right	< 0.5 °C
**22**	**Hand**	Back	Left	< 0.5 °C
**23**	**Thigh**	Front	Right	< 0.4 °C
**24**	**Thigh**	Anterior	Left	< 0.4 °C
**25**	**Lower leg**	Anterior	Right	< 0.5 °C
**26**	**Leg**	Anterior	Left	< 0.5 °C
**27**	**Foot**	Dorsal (anterior)	Right	< 0.6 °C
**28**	**Foot**	Dorsal side (anterior)	Left	< 0.6 °C
**29**	**Thigh**	Posterior	Right	< 0.4 °C
**30**	**Thigh**	Rear	Left	< 0.4 °C
**31**	**Leg**	Posterior	Right	< 0.5 °C
**32**	**Leg**	Rear	Left	< 0.5 °C
**33**	**Foot**	Plantar surface (posterior)	Right	< 0.6 °C
**34**	**Foot**	Plantar surface (posterior)	Left	< 0.6 °C

**35**	**Occiput**	posterior	Right	<0.4°C
**36**	**Occiput**	posterior	Left	<0.4°C

Table 1 – Main regions of interest in thermographic analysis (35.59)

2) Facial Thermographic Analysis – Interregional Comparison (Intrafacial ΔT)

Facial thermal variation analysis will be performed based on temperature differences **between six regions of interest on the face (ROIv)**, using an approach known as interregional analysis (intrafacial ΔT).
This method, described as the most robust in recent literature (39,59,73), is based on calculating temperature differences between areas whose skin reactivity varies depending on their autonomic innervation.

The six regions of interest **analyzed** are detailed in Table 2 and illustrated in Figure 2. They include: the nose (rapid vasoconstriction linked to sympathetic activation), the right and left periocular regions (highly sensitive to stress-related changes in blood flow), the chin, the perioral area, and the forehead, used as a stable thermal reference area.

The **intrafacial ΔT** will be calculated for the following pairs: Nose – Forehead; Periocular – Forehead; Chin – Forehead; Perioral – Forehead

A decrease of more than 0.5 °C in the central regions (nose, periocular) relative to the forehead is interpreted as a marker of sympathetic nervous system activation, indicating a response of stress or pain pain. This variation has been identified as significant in several studies using infrared thermography to assess emotional stress and pain in children (39,59,73).

The intrafacial approach has the advantage of being independent of variations in core body temperature or the environment, as the comparison is made between regions of the same face exposed to the same environmental conditions. It thus constitutes a noninvasive, stable, and reproducible indicator of physiological stress responses in infants.

The images will be analyzed by a specialized engineer listed on the delegation form prior to the start of the study.

ROIv Code ΔT	Anatomical Region	Side	Tolerated physiological
**ROIv1**	Central forehead (reference area)	Median	±0.2°C
**ROIv2**	Nose (nasal dorsum)	Median	±0.3 °C
**ROIv3**	Periocular region	Right	±0.3 °C

**ROIv4**	Periocular region	Left	±0.3 °C
**ROIv5**	Perioral region	Median	±0.3 °C
**ROIv6**	Chin region	Median	±0.3 °C

Table 2: Regions of Interest on the Face (ROIv)

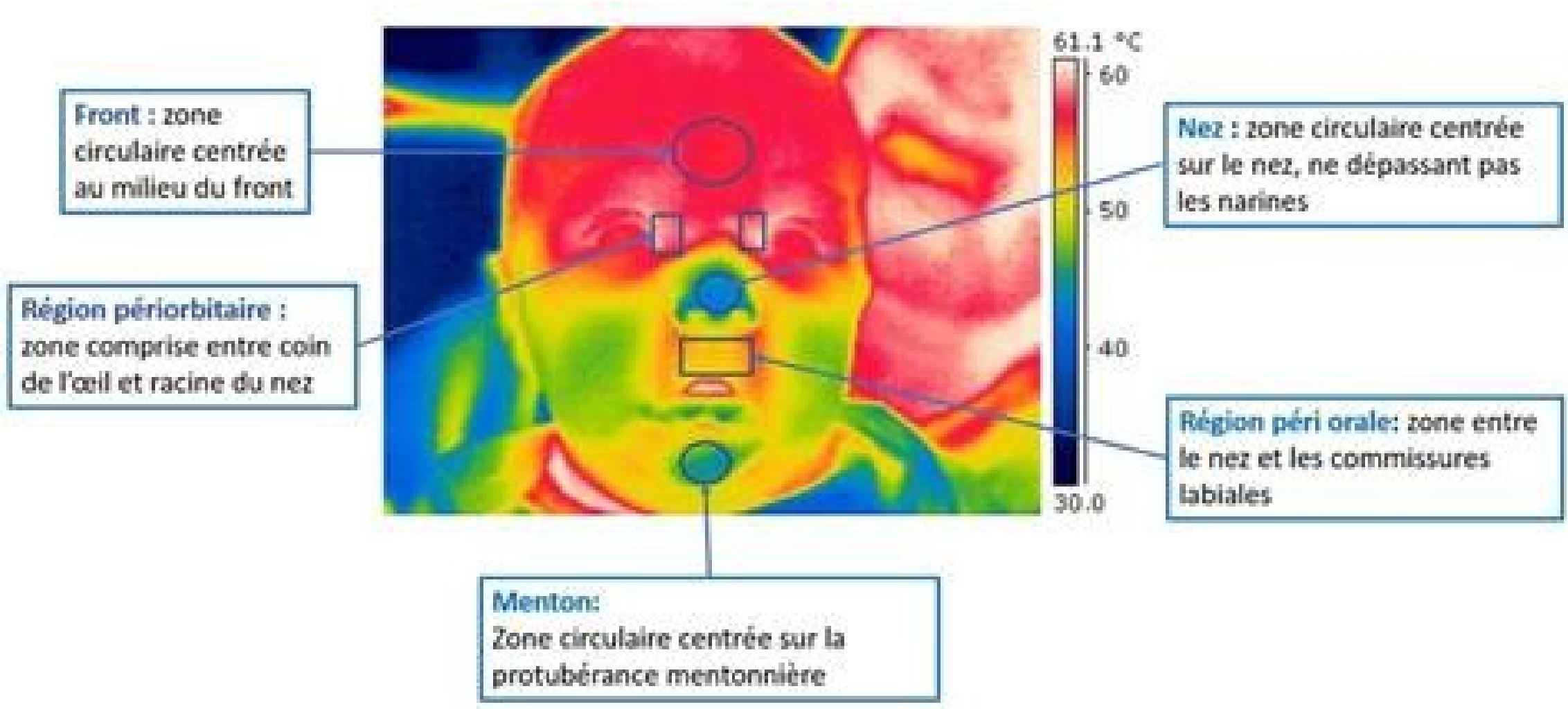

Figure 2: Regions of Interest on the Face

<u>Calculation and Interpretation of Intrafacial ΔT</u>

Temperature differences between facial regions (**intra-facial ΔT**) will be calculated for each facial region of interest (ROIv2 through ROIv5), using the **central forehead (ROIv1)** as the internal reference area.

$$\Delta T_{ROIvX} = T_{ROIvX} - T_{ROIv1}$$

where:

- T_{ROIvX} = average temperature recorded in region of interest X;
- T_{ROIv1} = average temperature of the central front.

<u>Interpretation Criteria</u> (Appendix 8)

A **negative ΔT** (relative cooling compared to the forehead) indicates sympathetic-mediated cutaneous vasoconstriction associated with a stress or pain response.

An **absolute difference greater than 0.5 °C** is considered clinically significant, exceeding the physiological variability observed in healthy infants (35,59).

Intrafacial ΔT values will be compared between the two study groups (physiological crying vs. excessive crying) at each assessment point (T1 and T2).

6.3. SECONDARY JUDGING CRITERIA

➢ **Analysis of general movements based on video recordings.**

The assessment will be conducted by two external observers—doctoral students or researchers from the CHARt laboratory—who will be listed on the delegation of duties form prior to the start of the study, trained in the analysis of general movements (GM Trust Basic and Advanced), and blinded to the group to which the child belongs. Depending on the infant's age, scores have been developed by the GM Trust team:

A GMOS-R score (General Movement Optimal Score—Revised, Appendix 2) (74) for preterm and term infants (from birth to 2 months), comprising a general analysis of the sequence, temporal organization, and quality of general movements (GM). It also includes a more detailed analysis by body region: trunk, head, and limbs. Each item is scored, with a maximum score of 38. This score will be used during Visit 1.

A MOS (Motor Optimal Score, Appendix 2) (72) for movements from 3 to 5 months of age, including the presence and quality of fidget movements (FM), the quality of other spontaneous movements, the age-appropriate motor repertoire, postural pattern, and movement characteristics. Each item is rated on a scale of 1 to 4. The optimal score is 25 to 28; movements are reduced starting at 25; and the prognosis is unfavorable below 14.

➢ **<u>Analysis of results from the Bayley Scales of Infant and Toddler Development (Bayley-4).</u>**

The Bayley scale will be administered by the investigators. The coordinating investigator, who is blinded to the infants' assignment group, will score the results. The administration will be videotaped in case of doubt regarding the scoring (65).

Raw scores (sums of items for each subtest) are entered into the electronic case report form (eCRF) as follows: COG_total, CR_total, CE_total, FM_total, GM_total. Raw scores (per subtest) will be calculated as the sum of item-by-item ratings (0, 1, 2). The maximum raw score will depend on the number of items administered, as defined by the start and stop rules in the Bayley-IV manual: Maximum raw score = (number of items administered) × 2. Raw scores will be converted to scale scores (1–19) and then to composite scores (approx. 40–160, mean 100 ±15) using the manual's normative tables and the Pearson Q-Global platform. Only the scale/composite scores will be retained in the final database (imported/exported from the platform).

➢ **<u>Capillary Cortisol Level Analysis</u>**:

The logistics of sample transport will be handled by clinical study technicians (CSTs) via secure channels. Shipments will be made at room temperature and will be consolidated to optimize costs. Each shipment will be tracked (sample ID, date, time, person responsible) and will be subject to an incident response procedure.

Upon arrival, the samples will be verified, registered, and stored at -20 °C in a dedicated freezer within the PRB of the Biochemistry and Endocrinology Laboratory at Robert Debré Hospital (coordinated by Prof. de Roux). The storage period and access to the freezer will be strictly controlled. After analysis, the samples will be stored until the end of the study and then destroyed in accordance with the laboratory's biological waste procedures. The PRB is ISO 20387 certified, ensuring the quality, traceability, and safety of sample management.

The hair cortisol assay will be performed at the Biochemistry and Endocrinology Laboratory at Robert Debré Hospital using high-performance liquid chromatography coupled with tandem mass spectrometry (HPLC-MS/MS). This method involves an initial separation of the compounds by liquid chromatography, followed by specific detection using triple-quadrupole mass spectrometry (XEVO TQS, Waters). This technique combines a liquid chromatography (HPLC) separation step with detection by tandem mass spectrometry (MS/MS), ensuring identification and a quantification specific of cortisol. The triple quadrupole enables the selection and fragmentation of ions characteristic of the molecule, ensuring **optimal precision and sensitivity**, which are particularly well-suited to the measurement of cortisol in hair, where concentrations are low. The method is validated according to international bioanalytical standards for this type of matrix. It is the gold standard for measuring cortisol in hair.

6.4. OTHER DATA COLLECTED

- **Socio-demographic and medical questionnaire (QA: Appendix 5):**

This questionnaire will collect information regarding age, sex, pregnancy, childbirth, diet, medical history, and family circumstances. These variables will enable a description of the population and an analysis of potential confounding factors.

- **ColiQ® Questionnaire (QB: Appendix 6)**

The **ColiQ** (*Assessment of the severity of infant crying and its impact on parents*, Bellaiche et al., *Archives de Pédiatrie*, 2021) (75) is a validated French-language questionnaire designed to assess the severity of an infant's crying and its impact on parents.

It consists of 16 questions divided into two modules:

"Symptoms" Module (Infant): 10 questions assessing the quantity and quality of crying, associated symptoms, perceived pain, and the infant's behavior.

"Impact" Module (Parent): 6 questions assessing the psychological, family, and daily-life impacts on parents.

Responses are collected on numerical scales ranging from 0 to 10 (0 = no symptoms / 10 = maximum severity) or as binary responses (Yes = 1 / No = 0). The subscores for each dimension are converted to a scale of 0 to 100, according to the rules defined by the authors.

Three composite scores are then calculated:

- Infant Well-being Score = 100 – (average of symptom subscores),
- Parent Well-being Score = 100 – (average of the impact subscores),
- Overall ColiQ Score = average of the two previous scores.

A higher score reflects a better overall state of the parent–infant dyad. The ColiQ will be used in this study to quantify the impact of crying and classify infants into three severity groups (mild, moderate, severe) according to the thresholds defined in the original publication:

- **Mild Severity Group**: ColiQ score > 70
- **Moderate Severity Group:** ColiQ score between 50 and 70
- **High Severity Group:** ColiQ score < 50

This questionnaire is not a primary outcome measure of the study; it will be used for descriptive purposes and statistical adjustment. The data will be entered and analyzed for descriptive purposes and may be used as covariates in multivariate analyses if necessary.

7. STATISTICAL ANALYSES

The statistical analysis plan was designed to address the study's primary objective—namely, the **validation of infrared thermal imaging (ITI)** as a tool for assessing chronic pain in infants with excessive crying—as well as the secondary objectives: to describe any associations between pain, stress, and psychomotor development.
Given the sample size (n = 40 per group) and the exploratory nature of certain hypotheses, the analyses were limited to simple, robust methods suitable for small sample sizes.

<u>Power and Minimum Detectable Effects</u>

With 40 infants per group (total N = 80), the study is designed to detect, at a significance level of $\alpha = 0.05$ and with 80% power:

- a **minimum correlation** of **$\rho \approx 0.31$** between the infrared thermal index (composite ITI) and the EDIN score (Spearman's correlation test);
- a **minimum between-group difference d ≈ 0.63** (moderate effect) on an aggregated thermal indicator (mean bilateral ΔT, central–peripheral ΔTcp, intrafacial ΔT);
- in longitudinal analysis (T1–T2), and assuming an intra-subject correlation of approximately 0.5, the detectable effect is reduced to **d ≈ 0.45–0.50** in mixed models.

The analyses will focus on presenting effect sizes (standardized ρ, d, β) and 95% confidence intervals in order to assess the clinical relevance and feasibility of thermal imaging thermal infrared in this context. Multiple comparisons will be prioritized: the main test (ITI–EDIN correlation) will take precedence, while secondary analyses will be interpreted as exploratory with alpha risk adjustment (FDR method).

Descriptive Analysis

The sociodemographic and clinical characteristics of the infants will be described according to the inclusion group (physiological crying vs. excessive crying):

- **Continuous variables**: means, standard deviations, medians, and interquartile ranges.
- **Categorical variables**: counts and percentages.

Classification of participants and management of status changes

Infants will be divided into two groups based on the average daily duration of crying observed during the week preceding the inclusion assessment, as reported in the crying diary:

- **"excessive crying" group**: ≥ 3 hours/day for at least 3 consecutive days, divided into 3 severity categories based on their ColiQ score.
- **"Physiological crying" group**: < 2 hours/day.

This classification will be retained for the primary analysis (*baseline* approach) to avoid bias related to changes in status after enrollment. However, the proportion of infants experiencing remission of crying between 1 and 3 months will be described and will be the subject of secondary analyses using three approaches:

- **"As-treated" analysis**, reclassifying infants according to their status at 3 months.
- **Longitudinal models with repeated measures**, taking into account the average daily duration of crying as a time-dependent variable.
- **Exploratory trajectory analyses** to identify patterns of change (persistence, remission, fluctuations).

7.1. PRIMARY OBJECTIVE: ITI–EDIN CONCORDANCE

The primary objective is to assess the concordance between **infrared thermal indices (ITI)**—including bilateral, central–peripheral, and intrafacial gradients—and **pain scores assessed using the EDIN behavioral scale.**

For each infant, average temperatures will be extracted from the regions of interest (ROI1–ROI34): central areas (thorax, abdomen) and peripheral areas (upper and lower limbs, bilateral); and facial regions (ROIv1–ROIv5): forehead, nose, periocular, perioral, and chin areas.

The **ITI thermal indices** will be calculated as follows:

- **Bilateral body ΔT** = |T right – T left|
- **ΔTcp (central–peripheral)** = central T – peripheral T
- **Intrafacial ΔT** = T zone – T forehead (forehead serving as a reference)

An **absolute difference greater than 0.5 °C** will be considered **abnormal asymmetry**.

This agreement will be analyzed using **Spearman's correlation coefficient**, which is suitable for nonparametric distributions, and supplemented by a **Bland-Altman plot** to evaluate the deviations between the two measures. **Mixed-effects linear models** (fixed effects: group, time, group × time interaction; random effect: subject) will be used to examine the joint evolution of ITI and EDIN scores.

7.3. SECONDARY OBJECTIVES

7.3.1. ASSOCIATION BETWEEN ITI, EDIN, AND CAPILLARY CORTISOL

Bivariate correlations (Spearman) between bilateral ΔT, ΔTcp, intrafacial ΔT, EDIN, and cortisol.

Adjusted linear regressions (age, sex, ambient temperature) to explore the determinants of thermal gradients and the interactions between physiological and behavioral markers of pain.

7.3.2. COMPARISON OF PSYCHOMOTOR DEVELOPMENT BETWEEN GROUPS

Motor and cognitive scores (Bayley-4) will be compared between groups using **the Mann–Whitney test.**

General Movement Analysis (GMA) will provide both:

- quantitative scores (GMOS-R at 1 month and MOS at 3 months), which will be compared across groups and incorporated into quantitative analyses (correlations, regressions, mixed models)

-Qualitative assessments (description of motor patterns: quality of movement, symmetry, fluidity, presence of episodes of abnormal movement), which will be described and compared qualitatively across groups.

Longitudinal analyses (mixed linear models) will be used to examine changes in psychomotor development between 1 and 3 months of age according to inclusion group and remission status.

Motor profiles (quantitative + qualitative) may be associated with pain indicators in exploratory analyses.

7.3.3. COLLABORATIONS BETWEEN PAIN ANDAND MOTOR PATTERNS

- Spearman correlations between motor characteristics (fluidity, symmetry, variability, GMOS-R score, MOS) and indicators of prolonged pain (bilateral ΔT, ΔTcp, intrafacial ΔT, EDIN, and cortisol).

- Exploratory analyses using simple linear regression to identify the thermal or behavioral markers most strongly associated with early motor impairments.

7.3.4. SIGNIFICANCE THRESHOLDS AND INTERPRETATION

The threshold for statistical significance will be set at $p < 0.05$ (two-tailed). Results will be accompanied by effect sizes (Cohen's d, Spearman's r, standardized β) and confidence intervals of confidence at 95 %. Analyses will be interpreted primarily based on their clinical relevance and the consistency of the observed trends, given the pilot nature of the study and the small sample size.
Complex multivariate analyses will be limited or exploratory to avoid overfitting.
Multiple comparisons will be prioritized according to the study objectives, and non-primary analyses will be subject to FDR (Benjamini–Hochberg) correction where appropriate. The overall interpretation will be based on the consistency among thermal, behavioral, and physiological markers, with the aim of validating the feasibility and sensitivity of infrared thermal imaging for the assessment of chronic pain in infants.

The results obtained will make it possible to identify trends and estimate the effect sizes needed to plan a larger-scale study, without claiming immediate generalizability of the conclusions.

8. DATA PROCESSING AND STORAGE OF DOCUMENTS AND RESEARCH-RELATED DATA

8.1. ACCESS TO DATA

Access to research data is strictly limited to authorized individuals listed on the delegation of duties form.

For the purposes of this study, only Ms. Joëlle Provasi (scientific coordinator), Ms. Camille Perrolet, and Ms. Isabelle Carchon (researchers) will have access to identifying data. Other individuals mentioned in the protocol as scientific leaders or members of the project's academic community will not have access to identifying data.

They may, if necessary, be involved in the scientific analysis of the results based on aggregated and anonymized data, without access to the source data.

The procedures for accessing, storing, and securing the data are carried out in accordance with the procedures approved by the sponsor and the URC at the Saint-Denis Hospital Center.

The sponsor is responsible for obtaining the consent of all parties involved in the research to ensure direct access to all research sites, source data, source documents, and reports for the purpose of quality control and audit by the sponsor;

Investigators shall make available to persons responsible for monitoring, quality control, or auditing research involving human subjects the individual documents and data strictly necessary for such oversight, in accordance with applicable laws and regulations (Articles L.1121-3 and R.5121-13 of the Public Health Code).

A quality control review will be conducted by a Clinical Research Associate provided by the sponsor. Since the sponsor has classified this study as requiring minimal monitoring given the low risk to participants, the Clinical Research Associate (CRA) will conduct 1 setup visit, 1 monitoring visit after at least 1 patient has been enrolled at each site, 1 monitoring visit at the end of the study, and a closure visit at each site. All data in the case report forms will be verified during the first monitoring visit. At the end of the study, 50% of the records (selected at random) will be monitored.

These visits will be conducted in accordance with the sponsor's standard operating procedures and will result in the preparation of visit reports.

8.2. SOURCE DOCUMENTS

Source documents are defined as any original document or item that serves as proof of the existence or accuracy of data or facts recorded during the clinical trial. They will be stored in the patients' electronic medical records and in the study's patient file.

8.3. DATA CONFIDENTIALITY

In accordance with the provisions regarding the confidentiality of data accessible to individuals responsible for quality control in research involving human subjects (Article L.1121-3 of the Public Health Code), and in accordance with the provisions regarding the confidentiality of information concerning, in particular, clinical trials, the participants, and the results obtained (Article R. 5121-13 of the Public Health Code), individuals with direct access to the data shall take all necessary precautions to ensure the confidentiality of information relating to the trials, the participants—particularly with regard to their identity—and the results obtained.

These individuals, just like the investigators themselves, are bound by professional confidentiality (under the conditions defined by Articles 226-13 and 226-14 of the Penal Code).

During research involving human subjects or upon its completion, the data collected on the participants and transmitted to the sponsor by the investigators (or any other specialized personnel) shall be anonymized.

Under no circumstances may the data reveal the names or addresses of the individuals concerned.

Patient data will be extracted using a study-specific coded language.

The sponsor will ensure that each research participant has provided written consent for access to their individual data, which is strictly necessary for the quality control of the study.

Investigators agree to comply with the requirements of the sponsor and the Competent Authority regarding any audit or inspection of the study.

An audit may be conducted at any stage of the study, from protocol development to the publication of results and the archiving of data used or generated as part of the study.

8.4. DATA MANAGEMENT

Study data will be entered into an eCRF, a web-based data capture system called Goupile, which will be made available by the URC of the GHT Plaine de France (in compliance with current standards).

The initial data entry of questionnaire data will be performed by the associate investigator, who will first collect the data on paper CRFs and then pseudonymize them (Questionnaires A, B, and C in paper format; crying diary in paper format).

Assistance from the GHT Plaine de France's clinical data entry technicians (TECs) for data entry may be considered at centers affiliated with the Saint-Denis and Gonesse Hospital Centers.

Each principal investigator will have access to the e-CRF for their center. Access may be granted to staff listed on the Form for Data Protection (FDF) with the principal investigator's approval. Access to the e-CRF is secure and restricted to authorized individuals.

Data collection is the responsibility of the clinical trial staff at the site, under the supervision of the center's principal investigator. The investigator must ensure the accuracy, completeness, legibility, and timeliness of the reported data by signing the e-CRF. All source documents must be completed carefully and legibly to ensure accurate reading of the data and proper transcription into the e-CRF.

The data recorded in the electronic form (e-CRF) derived from the source documents must be consistent with the data recorded on the source documents.

To be valid, data must be expressed in an appropriate unit and must appear in the source documents. The data will be frozen within one month of the end of patient enrollment, allowing for the correction or removal of data (GDPR).

The database will be backed up and retained for 2 years after the last publication.

8.5. DATA FLOW AND SECURITY

In accordance with the European General Data Protection Regulation (GDPR), information regarding patients' identities is considered confidential. Its disclosure to unauthorized persons is strictly prohibited.

Therefore, participants' identities will be replaced with a code number for the purposes of this study.

Data Pseudonymization

Upon signing the consent form to participate in this study, each patient will be assigned a unique identification number. This number will be used throughout the study according to the following procedure:

- Center number (01 to 03)
- Patient number (01 to 80 for each center): numbers assigned in order of enrollment

Example: 01 to 03 (depending on the center) – 01 to 80 (depending on the number of participants)

That is:

- Center 01 (Neonatology Department, CHSD):

01-01, 01-02, 01-03, …, 01-80

- Center 02 (Perinatal Psychiatry Department, CHSD):

02-01, 02-02, 02-03, …, 02-80

This structure allows for clear and systematic patient identification while ensuring the confidentiality of their data in accordance with the GDPR.

This unique identification number will be generated by the eCRF (electronic case report form) during the enrollment process. In all study documents and on all research specimens, this same patient identification code (center number and patient number) will be recorded to ensure confidentiality.

In accordance with Good Clinical Practice (GCP) requirements, the Investigator will maintain a "patient identification registry" containing the name and contact information of each patient, along with the assigned identification number. This registry and the signed consent forms will be kept strictly confidential in the Investigator's File at each center.

Informing Participants About the Use of Their Data

Participants will be informed via the information sheet and the consent form that their personal data collected as part of the study will be kept confidential and

will be processed by the Sponsor and its representatives. Patients' legal representatives will also be informed of their rights in accordance with the General Data Protection Regulation and French law.

Participants in this research will be informed, via the information sheet and the consent form, of the following rights:

- the nature and purpose of the data collected as part of the research, as well as the retention period for such data

- the option to withdraw from the study at any time and the fact that the sponsor will retain the information collected (unless otherwise specified by the data subject)

- their rights of access, rectification, objection, restriction, erasure, and data portability regarding the data collected as part of the research. These rights may be exercised at any time during the research either by submitting a request to the physician overseeing the participants in the research (who will contact the sponsor) or by submitting a request to the sponsor's Data Protection Officer

-The sponsor (through the TEC or the investigators) agrees to respond to any request for access to data within a maximum of one month. Furthermore, only personnel authorized by the sponsor (investigators, ARC, TEC) and representatives of health authorities may have access to this information.

Data Storage

Home videos recorded by parents (on Days 15, 30, and 45 after Visit 1) will be saved on dedicated USB drives labeled with the patient's study number and stored in a locked cabinet at the URC facilities of the GHT Plaine de France. The USB drives will be formatted at the end of the study.

Footage recorded using thermal and digital cameras, as well as during the Bayley IV test, will be saved on an external hard drive dedicated to the project. A folder labeled with the study number will be created for each patient, and all footage corresponding to each patient will be filed in that folder. The external hard drive will be kept in a locked cabinet at the GHT Plaine de France URC facilities. It will be formatted at the end of the study.

Data entered into the Goupile e-CRF will be stored on secure Health Data Hosting servers, in accordance with the agreement established between the Saint-Denis Hospital Center and the Interhop association (3 Lieu-dit Val Jourdan, 35120 SAINT-MARCAN).

The database extracted from the Goupile e-CRF will be password-protected and stored on the secure servers of the Saint-Denis Hospital Center, the study sponsor. It will also be shared with the EPHE team—specifically, Ms. Joëlle Provasi and Ms. Camille Perrolet (via "BlueFiles")—who will perform the analyses.

The pseudonymized database will be stored on secure EPHE servers.

No data will be transferred outside the European Union.

8.6. PATIENT INFORMATION AND CONSENT

No research involving minimal risk and minimal burden may be conducted on a person without their prior written consent, obtained after the physician has provided them with the information required under Article L.1122-1.

- General information regarding research activities at the facility must be provided to the individuals concerned (posters on the premises, mention in the welcome booklet, etc.).
- Individuals participating in research and/or their legal representatives are informed in advance and individually about the processing of their personal data, including:
  - The nature of the information provided.
  - The purpose of the processing of their data.
  - The natural or legal persons to whom their data will be disclosed.
  - The right of access and rectification established in Articles 39 and 40 of the Data Protection Act.
  - The right to object, as provided for in the first and third paragraphs of Article 56 of the Data Protection Act.

This information must be included in the privacy notice.

Research participants and/or their legal representatives are also informed that their participation in the research is voluntary and that they may object to the use of their data without having to provide a reason.

Patients included in this study will be treated in accordance with standard clinical practices and in compliance with Good Clinical Practice guidelines.

8.7. FINAL RESEARCH REPORT

The final report on research involving human subjects referred to in Article R1123-67 of the Public Health Code (CSP) shall be prepared and signed by the sponsor and the investigator. A summary of the report, prepared in accordance with the competent authority's reference template, must be submitted to the competent authority within one year of the end of the research, which corresponds to the end of the participation of the last person taking part in the research.

8.8. CNIL

This study falls within the scope of the "Reference Methodology" (MR-001) pursuant to the provisions of Article 54, paragraph 5, of Law No. 78-17 of January 6, 1978, as amended, relating to information technology, files, and civil liberties. This change was approved by a decision dated January 5, 2006, and amended on July 21, 2016. The Clinical Research Unit at Saint-Denis Hospital, the study sponsor, has signed a commitment to comply with this "Reference Methodology."

The data collected as part of this study are gathered for scientific research purposes, in the public interest.

8.9. ARCHIVING

The investigator agrees to properly maintain all study documentation and records to ensure complete documentation of the study's conduct and to allow for subsequent verification of the study data.

Upon completion of the study, investigators must archive all study files and documents, eCRFs on CD-ROM, copies of the study product liability forms, copies of approvals from the Institutional Review Board (IRB)/regulatory authorities, and all correspondence in a safe and secure location. Trial-related documents must be retained at the investigator's site for 15 years in accordance with European Directive 2003/63/EC and local legal requirements. The investigator or their institution must take measures to prevent the accidental or premature destruction of these documents.

- Protocol and appendices (identified by version number and version date), any amendments,
- Original signed informed consent forms
- Individual data (certified copies of raw data)
- Follow-up documents
- Statistical analyses
- Final study report

No removal or destruction may be carried out without the Sponsor's consent. At the end of the 15-year period, the Sponsor will be consulted regarding destruction. All data, documents, and reports may be subject to audit or inspection.

8.10. INSURANCE

In accordance with Article L.1121-10 of the Public Health Code, insurance policies must cover the civil liability of the sponsor and that of any participant, and must cover the financial consequences of claims arising from research involving human subjects.

The sponsor has taken out insurance for the entire duration of the research that covers its own civil liability, as well as that of all personnel involved in conducting the research.
It also guarantees full compensation for any harmful consequences of the research to the research participant and his or her beneficiaries, unless the company can prove that the harm was not attributable to its own fault or that of any other party involved; the actions of a third party or the voluntary withdrawal of the person who initially consented to participate in the research may not be invoked as a defense.

The Saint-Denis Hospital Center has taken out insurance with LLOYD'S INSURANCE COMPANY SA, located at 8 rue Lamennais, 75008 PARIS, under policy number

No. B1339CTLICNWL24-54, covering its civil liability as well as that of any participant (physician or staff involved in conducting the research), in accordance with Article L.1121-10 of the CSP.

9. ETHICAL CONSIDERATIONS

This study falls under the Jardé Law (Law No. 2012-300 of March 5, 2012); it is a human subjects research study classified as RIPH2, specifically designated as Interventional Research with Minimal Risks and Constraints (RIRCM).

The study will be launched after receiving approval from a Human Subjects Protection Committee (*CPP*, *to be designated*) and after notifying the ANSM.

This study falls within the scope of the "Reference Methodology" (MR-001) pursuant to the provisions of Article 54, paragraph 5, of Law No. 78-17 of January 6, 1978, as amended, relating to information technology, data files, and civil liberties. This change was approved by a decision dated January 5, 2006, and amended on July 21, 2016.

This project received the support of the Scientific and Ethical Review Committee of the GHT Plaine de France on November 15, 2024.

It is registered on the "clinicaltrials.gov" website under NCT number *(pending)*

10. PUBLICATION POLICY

The publication guidelines for scientific articles are described below (http://www.icmje.org/).

Anyone designated as an author must be qualified to hold that title: Each author must have contributed sufficiently to the work to take public responsibility for all or part of its content. The credibility of authorship is based on essential contributions:

- Design and methodology and/or analysis and interpretation of results
- Drafting of the article or critical revisions with significant contribution to the intellectual content
- Final approval of the published version

Order of authorship (coordinating and associate investigators, methodologist, principal investigators from participating centers): The order will reflect the various investigators' contributions to the trial (number of patients enrolled and evaluable) and those who made significant contributions during its conduct.

A statement will indicate the source of funding.

In the case of ancillary studies, their results may be published only with the consent of the principal investigators and the methodologist, and only after publication of the main study, which must be cited.

The coordinating investigator will sign the final version of the clinical trial report for this study, thereby indicating agreement with the analyses, results, and conclusions of the report.

11. BIBLIOGRAPHY

1. Goksan S, Hartley C, Emery F, Cockrill N, Poorun R, Moultrie F, et al. fMRI reveals neural activity overlap between adult and infant pain. eLife. Apr 21, 2015;4. doi:10.7554/eLife.06356 PubMed PMID: 25895592; PubMed Central PMCID: PMC4402596.

2. Fitzgerald M. The development of nociceptive circuits. Nat Rev Neurosci. July 2005;6(7):507-20. doi:10.1038/nrn1701

3. Ranger M, Grunau RE. How do babies feel pain? eLife. Apr 27, 2015;4:e07552. doi:10.7554/eLife.07552 PubMed PMID: 25915737; PubMed Central PMCID: PMC4410745.

4. Melchior M, Kuhn P, Poisbeau P. The burden of early-life stress on the development of the nociceptive system and pain responses. Eur J Neurosci. May 2022;55(9-10):2216-41. doi:10.1111/ejn.15153 PubMed PMID: 33615576.

5. Anand KJS. Defining pain in newborns: need for a uniform taxonomy? Acta Paediatr. Sept. 2017;106(9):1438-44. doi:10.1111/apa.13936 PubMed PMID: 28556311; PubMed Central PMCID: PMC5601230.

6. Schwaller F, Fitzgerald M. The consequences of pain in early life: injury-induced plasticity in developing pain pathways. Eur J Neurosci. Feb 2014;39(3):344-52. doi:10.1111/ejn.12414 PubMed PMID: 24494675; PubMed Central PMCID: PMC4264936.

7. Chang P, Fabrizi L, Fitzgerald M. Early-Life Pain Experience Changes Adult Functional Pain Connectivity in the Rat Somatosensory and Medial Prefrontal Cortex. J Neurosci. Nov 2, 2022;42(44):8284-96. doi:10.1523/JNEUROSCI.0416-22.2022 PubMed PMID: 36192150; PubMed Central PMCID: PMC9653276.

8. Douglas PS, Hill PS. A neurobiological model for crying and fussing problems in the first three to four months of life. Med Hypotheses. Nov 2013;81(5):816–22. doi:10.1016/j.mehy.2013.09.004 PubMed PMID: 24075595.

9. Bilgin A, Heinonen K, Girchenko P, Kajantie E, Wolke D, Räikkönen K. Early childhood multiple or persistent regulatory problems and diurnal salivary cortisol in young adulthood. Psychoneuroendocrinology. March 2024;161:106940. doi:10.1016/j.psyneuen.2023.106940 PubMed PMID: 38171041.

10. Douglas PS. Pre-emptive Intervention for Autism Spectrum Disorder: Theoretical Foundations and Clinical Translation. Front Integr Neurosci. 2019;13:66.

doi:10.3389/fnint.2019.00066 PubMed PMID: 31798425; PubMed Central PMCID: PMC6877903.

11. Williams MD, Lascelles BDX. Early Neonatal Pain—A Review of Clinical and Experimental Implications for Painful Conditions Later in Life. Front Pediatr. 2020;8:30. doi:10.3389/fped.2020.00030 PubMed PMID: 32117835; PubMed Central PMCID: PMC7020755.

12. Duff IT, Krolick KN, Mahmoud HM, Chidambaran V. Current Evidence for Biological Biomarkers and Mechanisms Underlying the Transition from Acute to Chronic Pain Across the Pediatric Age Spectrum. J Clin Med. August 9, 2023;12(16):5176. doi:10.3390/jcm12165176 PubMed PMID: 37629218; PubMed Central PMCID: PMC10455285.

13. Zouikr I, Karshikoff B. Lifetime Modulation of the Pain System via Neuroimmune and Neuroendocrine Interactions. Front Immunol. 2017;8:276. doi:10.3389/fimmu.2017.00276 PubMed PMID: 28348566; PubMed Central PMCID: PMC5347117.

14. Vermillet A, Tølbøll K, Litsis Mizan S, C. Skewes J, Parsons CE. Crying in the first 12 months of life: A systematic review and meta-analysis of cross-country parent-reported data and modeling of the "cry curve." Child Dev. 2022;93(4):1201-22. doi:10.1111/cdev.13760 PubMed PMID: 35438798; PubMed Central PMCID: PMC9541248.

15. Zeevenhooven J, de Bruin FE, Schappin R, Vlieger AM, van der Lee JH, Haverman L, et al. Follow-up of infants with colic into childhood: Do they develop behavioral problems? J Paediatr Child Health. Nov 2022;58(11):2076-83. doi:10.1111/jpc.16174 PubMed PMID: 36054703; PubMed Central PMCID: PMC9804625.

16. Wolke D, Bilgin A, Samara M. Systematic Review and Meta-Analysis: Duration of Fussing and Crying and Prevalence of Colic in Infants. J Pediatr. June 2017;185:55-61.e4. doi:10.1016/j.jpeds.2017.02.020 PubMed PMID: 28385295.

17. Ellwood J, Draper-Rodi J, Carnes D. Comparison of common interventions for the treatment of infantile colic: a systematic review of reviews and guidelines. BMJ Open. Feb. 25, 2020;10(2):e035405. doi:10.1136/bmjopen-2019-035405 PubMed PMID: 32102827; PubMed Central PMCID: PMC7202698.

18. Munck P, Maunu J, Kirjavainen J, Lapinleimu H, Haataja L, Lehtonen L, et al. Crying behavior in early infancy is associated with developmental outcomes at two years of age in very low birth weight infants. Acta Paediatr. March 2008;97(3):332-6. doi:10.1111/j.1651-2227.2008.00673.x PubMed PMID: 18298782.

19. Valla L, Småstuen MC, Andenæs R, Misvær N, Olbjørn C, Helseth S. Association between colic and sleep problems in infancy and subsequent development, emotional, and behavioral problems: a longitudinal study. BMC Pediatr. Jan. 7, 2021;21(1):23. doi:10.1186/s12887-020-02483-1 PubMed PMID: 33407244; PubMed Central PMCID: PMC7788887.

20. St James-Roberts I, Garratt R, Powell C, Bamber D, Long J, Brown J, et al. A support package for parents of excessively crying infants: development and feasibility study.

Health Technol Assess. Oct 2019;23(56):1-144. doi:10.3310/hta23560 PubMed PMID: 31597591; PubMed Central PMCID: PMC6801366.

21. Bellaiche M, Arnould B, Benmedjahed K, Arnould M, Bocquet A, Leblanc V, et al. Assessment of the severity of infant crying and its impact on parents: Development and validation of the ColiQ Questionnaire in France. Archives de Pédiatrie. May 1, 2021;28(4):264-72. doi:10.1016/j.arcped.2021.02.018

22. Pillai Riddell RR, Stevens BJ, McKeever P, Gibbins S, Asztalos L, Katz J, et al. Chronic pain in hospitalized infants: health professionals' perspectives. J Pain. Dec 2009;10(12):1217-25. doi:10.1016/j.jpain.2009.04.013 PubMed PMID: 19541547.

23. Ten Barge JA, Baudat M, Meesters NJ, Kindt A, Joosten EA, Reiss IKM, et al. Biomarkers for assessing pain and pain relief in the neonatal intensive care unit. Front Pain Res (Lausanne). 2024;5:1343551. doi:10.3389/fpain.2024.1343551 PubMed PMID: 38426011; PubMed Central PMCID: PMC10902154.

24. van der Vaart M, Duff E, Raafat N, Rogers R, Hartley C, Slater R. Multimodal pain assessment improves discrimination between noxious and non-noxious stimuli in infants. Paediatr Neonatal Pain. Sept 2019;1(1):21-30. doi:10.1002/pne2.12007 PubMed PMID: 35546868; PubMed Central PMCID: PMC8974881.

25. Salekin MS, Mouton PR, Zamzmi G, Patel R, Goldgof D, Kneusel M, et al. Future roles of artificial intelligence in early pain management of newborns. Paediatr Neonatal Pain. Sept. 2021;3(3):134-45. doi:10.1002/pne2.12060 PubMed PMID: 35547946; PubMed Central PMCID: PMC8975206.

26. Yamada J, Stevens B, de Silva N, Gibbins S, Beyene J, Taddio A, et al. Hair cortisol as a potential biological marker of chronic stress in hospitalized neonates. Neonatology. 2007;92(1):42-9. doi:10.1159/000100085 PubMed PMID: 17356304.

27. Hatfield LA. Neonatal pain: What's age got to do with it? Surg Neurol Int. 2014;5(Suppl 13):S479-489. doi:10.4103/2152-7806.144630 PubMed PMID: 25506507; PubMed Central PMCID: PMC4253046.

28. Debillon T, Zupan V, Ravault N, Magny JF, Dehan M. Development and initial validation of the EDIN scale, a new tool for assessing prolonged pain in preterm infants. Arch Dis Child Fetal Neonatal Ed. July 2001;85(1):F36-41. doi:10.1136/fn.85.1.f36 PubMed PMID: 11420320; PubMed Central PMCID: PMC1721270.

29. Zamzmi G, Kasturi R, Goldgof D, Zhi R, Ashmeade T, Sun Y. A Review of Automated Pain Assessment in Infants: Features, Classification Tasks, and Databases. IEEE Rev Biomed Eng. 2018;11:77-96. doi:10.1109/RBME.2017.2777907 PubMed PMID: 29989992.

30. M van der V, E D, N R, R R, C H, R S. Multimodal pain assessment improves discrimination between noxious and non-noxious stimuli in infants. Paediatric & neonatal pain. Sept. 9, 2019;1(1). doi:10.1002/pne2.12007 PubMed PMID: 35546868.

31. Heiderich TM, Carlini LP, Buzuti LF, Balda R de CX, Barros MCM, Guinsburg R, et al. Face-based automatic pain assessment: challenges and perspectives in neonatal intensive

care units. J Pediatr (Rio J). 2023;99(6):546-60. doi:10.1016/j.jped.2023.05.005 PubMed PMID: 37331703; PubMed Central PMCID: PMC10594024.

32. Cardone D, Merla A. New Frontiers for Applications of Thermal Infrared Imaging Devices: Computational Psychophysiology in the Neurosciences. Sensors (Basel). May 5, 2017;17(5):E1042. doi:10.3390/s17051042 PubMed PMID: 28475155; PubMed Central PMCID: PMC5469647.

33. AlZubaidi AK, Ethawi Y, Schmölzer GM, Sherif S, Narvey M, Seshia M. Review of Biomedical Applications of Contactless Imaging of Neonates Using Infrared Thermography and Beyond. Methods Protoc. Oct. 29, 2018;1(4):E39. doi:10.3390/mps1040039 PubMed PMID: 31164579; PubMed Central PMCID: PMC6481091.

34. Merla A, Di Donato L, Romani GL, Proietti M, Salsano F. Comparison of thermal infrared and laser Doppler imaging in the assessment of cutaneous tissue perfusion in scleroderma patients and healthy controls. Int J Immunopathol Pharmacol. Sept 2008;21(3):679-86. doi:10.1177/039463200802100322 PubMed PMID: 18831936.

35. Marins JCB, Fernandes AA, Cano SP, Moreira DG, da Silva FS, Costa CMA, et al. Thermal body patterns for healthy Brazilian adults (male and female). J Therm Biol. May 2014;42:1-8. doi:10.1016/j.jtherbio.2014.02.020 PubMed PMID: 24802142.

36. Uematsu S, Edwin DH, Jankel WR, Kozikowski J, Trattner M. Quantification of thermal asymmetry. Part 1: Normal values and reproducibility. J Neurosurg. Oct. 1988;69(4):552-5. doi:10.3171/jns.1988.69.4.0552 PubMed PMID: 3418388.

37. Haddad DS, Brioschi ML, Arita ES. Thermographic and clinical correlation of myofascial trigger points in the masticatory muscles. Dentomaxillofac Radiol. Dec 2012;41(8):621-9. doi:10.1259/dmfr/98504520 PubMed PMID: 23166359; PubMed Central PMCID: PMC3528192.

38. Doménech-García V, Peirotén AR, Imaz ML, Palsson TS, Herrero P, Bellosta-López P. Not just sensitization: sympathetic mechanisms contribute to the spread of experimental referred pain. Korean J Pain. July 1, 2022;35(3):240-9. doi:10.3344/kjp.2022.35.3.240 PubMed PMID: 35768979; PubMed Central PMCID: PMC9251400.

39. Ioannou S, Ebisch S, Aureli T, Bafunno D, Ioannides HA, Cardone D, et al. The autonomic signature of guilt in children: a thermal infrared imaging study. PLoS One. 2013;8(11):e79440. doi:10.1371/journal.pone.0079440 PubMed PMID: 24260220; PubMed Central PMCID: PMC3834185.

40. Shastri D, Merla A, Tsiamyrtzis P, Pavlidis I. Imaging facial signs of neurophysiological responses. IEEE Trans Biomed Eng. Feb 2009;56(2):477-84. doi:10.1109/TBME.2008.2003265 PubMed PMID: 19272941.

41. Kretschmer SCA, Paul M, Heussen N, Leonhardt S, Orlikowsky T, Heimann K. Facial thermal response to non-painful stressors in premature and term neonates. Pediatr Res. Oct 2023;94(4):1422-7. doi:10.1038/s41390-023-02614-1 PubMed PMID: 37161075; PubMed Central PMCID: PMC10589090.

42. Ioannou S, Morris P, Mercer H, Baker M, Gallese V, Reddy V. Proximity and gaze influence facial temperature: a thermal infrared imaging study. Front Psychol. 2014;5:845. doi:10.3389/fpsyg.2014.00845 PubMed PMID: 25136326; PubMed Central PMCID: PMC4120854.

43. Knobel RB, Guenther BD, Rice HE. Thermoregulation and thermography in neonatal physiology and disease. Biol Res Nurs. July 2011;13(3):274-82. doi:10.1177/1099800411403467 PubMed PMID: 21586499; PubMed Central PMCID: PMC3775585.

44. Shi Y, Payeur P, Frize M, Bariciak E. Infrared Imaging Tools for the Diagnosis of Necrotizing Enterocolitis (NEC) Guided by RGB-D Sensing. CMBES Proceedings [Internet]. May 21, 2019 [cited Oct. 6, 2023];42. Available from: https://proceedings.cmbes.ca/index.php/proceedings/article/view/848

45. Gratt BM, Sickles EA, Ross JB, Wexler CE, Gornbein JA. Thermographic assessment of craniomandibular disorders: diagnostic interpretation versus temperature measurement analysis. J Orofac Pain. 1994;8(3):278-88. PubMed PMID: 7812225.

46. Ford RG, Ford KT. Thermography in the diagnosis of headache. Semin Neurol. 1997;17(4):343-9. doi:10.1055/s-2008-1040947 PubMed PMID: 9474714.

47. Costa ACS, Dibai Filho AV, Packer AC, Rodrigues-Bigaton D. Intra- and inter-rater reliability of infrared image analysis of masticatory and upper trapezius muscles in women with and without temporomandibular disorder. Braz J Phys Ther. Feb 2013;17(1):24-31. doi:10.1590/s1413-35552012005000058 PubMed PMID: 23117649.

48. Skorupska E, Dybek T, Rychlik M, Jokiel M, Dobrakowski P. The Automation of a New Thermography Method Using Invasive Nociceptive Stimulation to Confirm an Autonomic Phenomenon within a Trigger Point Referred Pain Zone. Brain Sci. July 6, 2021;11(7):893. doi:10.3390/brainsci11070893 PubMed PMID: 34356127; PubMed Central PMCID: PMC8301943.

49. Owen R, Ramlakhan S, Saatchi R, Burke D. Development of a high-resolution infrared thermographic imaging method as a diagnostic tool for acute undifferentiated limp in young children. Med Biol Eng Comput. June 2018;56(6):1115-25. doi:10.1007/s11517-017-1749-0 PubMed PMID: 29181625; PubMed Central PMCID: PMC5978821.

50. Mota-Rojas D, Olmos-Hernández A, Verduzco-Mendoza A, Lecona-Butrón H, Martínez-Burnes J, Mora-Medina P, et al. Infrared thermal imaging associated with pain in laboratory animals. Exp Anim. Feb 6, 2021;70(1):1-12. doi:10.1538/expanim.20-0052 PubMed PMID: 32848100; PubMed Central PMCID: PMC7887630.

51. Alfieri FM, Battistella LR. Body temperature of healthy men evaluated by thermography: A study of reproducibility. Technol Health Care. 2018;26(3):559-64. doi:10.3233/THC-171164 PubMed PMID: 29578493.

52. Nahm FS. Infrared thermography in pain medicine. Korean J Pain. July 2013;26(3):219-22. doi:10.3344/kjp.2013.26.3.219 PubMed PMID: 23861995; PubMed Central PMCID: PMC3710935.

53. Park ES, Park CI, Jung KI, Chun S. Comparison of sympathetic skin response and digital infrared thermographic imaging in peripheral neuropathy. Yonsei Med J. Dec 1994;35(4):429-37. doi:10.3349/ymj.1994.35.4.429 PubMed PMID: 7871847.

54. Zhang HY, Chin DK, Cho YE, Kim YS. Correlation between Pain Scale and Infrared Thermogram in Lumbar Disc Herniations. Journal of the Korean Neurosurgical Society. 28(2):253-8.

55. Abbas AK, Leonhardt S. Intelligent neonatal monitoring based on a virtual thermal sensor. BMC Med Imaging. March 2, 2014;14:9. doi:10.1186/1471-2342-14-9 PubMed PMID: 24580961; PubMed Central PMCID: PMC4015601.

56. Czaplik M, Hochhausen N, Dohmeier H, Pereira CB, Rossaint R. Development of a "Thermal-Associated Pain Index" score using infrared thermography for objective pain assessment. Annu Int Conf IEEE Eng Med Biol Soc. July 2017;2017:3831-4. doi:10.1109/EMBC.2017.8037692 PubMed PMID: 29060733.

57. Aureli T, Grazia A, Cardone D, Merla A. Behavioral and facial thermal variations in 3- to 4-month-old infants during the Still-Face Paradigm. Front Psychol. 2015;6:1586. doi:10.3389/fpsyg.2015.01586 PubMed PMID: 26528229; PubMed Central PMCID: PMC4604256.

58. Giordano V, Deindl P, Gal E, Unterasinger L, Fuiko R, Steinbauer P, et al. Pain and neurodevelopmental outcomes of infants born very preterm. Dev Med Child Neurol. Aug. 2023;65(8):1043-52. doi:10.1111/dmcn.15505 PubMed PMID: 36647629.

59. Kolosovas-Machuca ES, González FJ. Distribution of skin temperature in Mexican children. Skin Res Technol. Aug. 2011;17(3):326-31. doi:10.1111/j.1600-0846.2011.00501.x PubMed PMID: 21338404.

60. Reyes Del Paso GA, Garcia-Hernandez A, Contreras-Merino AM, Galvez-Sánchez CM, de la Coba P, Montoro CI, et al. A two-component model of hair cortisol concentration in fibromyalgia: Independent effects of pain chronicity and severity. Eur J Pain. May 2024;28(5):821-30. doi:10.1002/ejp.2223 PubMed PMID: 38102835.

61. Defelipe RP, Terra J, Francischelli I, Pacheco B, Araújo PP, Mesquita AR, et al. Hair collection protocol in 12-month-old infants. Compr Psychoneuroendocrinol. Aug. 2024;19:100243. doi:10.1016/j.cpnec.2024.100243 PubMed PMID: 39070240; PubMed Central PMCID: PMC11279705.

62. Nist MD, Sullenbarger BA, Harrison TM, Pickler RH. Protocol for Measuring Cortisol in Hair from Low-Mass Samples of Very Preterm Infants. Nurs Res. 2020;69(4):316-21. doi:10.1097/NNR.0000000000000436 PubMed PMID: 32287079; PubMed Central PMCID: PMC7610218.

63. Einspieler C, Prechtl HFR. Prechtl's assessment of general movements: a diagnostic tool for the functional assessment of the young nervous system. Ment Retard Dev Disabil Res Rev. 2005;11(1):61-7. doi:10.1002/mrdd.20051 PubMed PMID: 15856440.

64. Hadders-Algra M. Neural substrate and clinical significance of general movements: an update. Dev Med Child Neurol. Jan 2018;60(1):39-46. doi:10.1111/dmcn.13540 PubMed PMID: 28832987.

65. Yue A, Jiang Q, Wang B, Abbey C, Medina A, Shi Y, et al. Concurrent validity of the Ages and Stages Questionnaire and the Bayley Scales of Infant Development III in China. PLoS One. Sept. 5, 2019;14(9):e0221675. doi:10.1371/journal.pone.0221675 PubMed PMID: 31487302; PubMed Central PMCID: PMC6728026.

66. Spittle AJ, Olsen J, Kwong A, Doyle LW, Marschik PB, Einspieler C, et al. The Baby Moves prospective cohort study protocol: using a smartphone application with the General Movements Assessment to predict neurodevelopmental outcomes at age 2 years for extremely preterm or extremely low birth weight infants. BMJ Open. Oct. 3, 2016;6(10):e013446. doi:10.1136/bmjopen-2016-013446 PubMed PMID: 27697883; PubMed Central PMCID: PMC5073614.

67. Zeevenhooven J, Koppen IJN, Benninga MA. The New Rome IV Criteria for Functional Gastrointestinal Disorders in Infants and Toddlers. Pediatr Gastroenterol Hepatol Nutr. March 2017;20(1):1-13. doi:10.5223/pghn.2017.20.1.1 PubMed PMID: 28401050; PubMed Central PMCID: PMC5385301.

68. St James-Roberts I, Alvarez M, Csipke E, Abramsky T, Goodwin J, Sorgenfrei E. Infant crying and sleeping in London, Copenhagen, and when parents adopt a “proximal” form of care. Pediatrics. June 2006;117(6):e1146-1155. doi:10.1542/peds.2005-2387 PubMed PMID: 16740816.

69. Ferrari F, Frassoldati R, Berardi A, Di Palma F, Ori L, Lucaccioni L, et al. The ontogeny of fidgety movements from 4 to 20 weeks post-term age in healthy full-term infants. Early Hum Dev. Dec 2016;103:219-24. doi:10.1016/j.earlhumdev.2016.10.004 PubMed PMID: 27825041.

70. Julious SA. Pilot studies in clinical research. Stat Methods Med Res. June 2016;25(3):995-6. doi:10.1177/0962280216651022 PubMed PMID: 27207088.

71. Hertzog MA. Considerations in determining sample size for pilot studies. Res Nurs Health. Apr 2008;31(2):180-91. doi:10.1002/nur.20247 PubMed PMID: 18183564.

72. Ring EFJ, Ammer K. Infrared thermal imaging in medicine. Physiol Meas. Mar 2012;33(3):R33-46. doi:10.1088/0967-3334/33/3/R33 PubMed PMID: 22370242.

73. Ioannou S. Functional Infrared Thermal Imaging: A Contemporary Tool in Soft Tissue Screening. Sci Rep. June 9, 2020;10(1):9303. doi:10.1038/s41598-020-66397-9 PubMed PMID: 32518287; PubMed Central PMCID: PMC7283266.

74. Einspieler C, Bos AF, Spittle AJ, Bertoncelli N, Burger M, Peyton C, et al. The General Movement Optimality Score-Revised (GMOS-R) with Socioeconomically Stratified Percentile Ranks. J Clin Med. Apr 13, 2024;13(8):2260. doi:10.3390/jcm13082260 PubMed PMID: 38673533; PubMed Central PMCID: PMC11050782.

75. Bellaiche M, Arnould B, Benmedjahed K, Arnould M, Bocquet A, Leblanc V, et al. Assessment of the severity of infant crying and its impact on parents: Development and validation of the ColiQ Questionnaire in France. Arch Pediatr. May 2021;28(4):264-72. doi:10.1016/j.arcped.2021.02.018 PubMed PMID: 33812728.

12. APPENDICES

APPENDIX 1: NEWBORN PAIN AND DISCOMFORT SCALE (EDIN)

Echelle de douleur et d'inconfort du nouveau-né (EDIN)
élaborée et validée pour le nouveau-né à terme ou prématuré
utilisable jusqu'à 6 à 9 mois
pour mesurer un état douloureux prolongé (lié à une maladie ou à une intervention chirurgicale ou à la répétition fréquente de gestes invasifs)
non adaptée à la mesure d'une douleur aiguë comme celle d'un soin isolé.
score de 0 à 15, seuil de traitement 5

	**Date**								
	**Heure**								
**VISAGE**	**0** Visage détendu<br>**1** Grimaces passagères : froncement des sourcils / lèvres pincées / plissement du menton / tremblement du menton<br>**2** Grimaces fréquentes, marquées ou prolongées<br>**3** Crispation permanente **ou** visage prostré, figé **ou** visage violacé								
**CORPS**	**0** Détendu<br>**1** Agitation transitoire, assez souvent calme<br>**2** Agitation fréquente mais retour au calme possible<br>**3** Agitation permanente, crispation des extrémités, raideur des membres **ou** motricité très pauvre et limitée, avec corps figé								
**SOMMEIL**	**0** S'endort facilement, sommeil prolongé, calme<br>**1** S'endort difficilement<br>**2** Se réveille spontanément en dehors des soins et fréquemment, sommeil agité<br>**3** Pas de sommeil								
**RELATION**	**0** Sourire aux anges, sourire-réponse, attentif à l'écoute<br>**1** Appréhension passagère au moment du contact<br>**2** Contact difficile, cri à la moindre stimulation<br>**3** Refuse le contact, aucune relation possible. Hurlement ou gémissement sans la moindre stimulation								
**RECONFORT**	**0** N'a pas besoin de réconfort<br>**1** Se calme rapidement lors des caresses, au son de la voix ou à la succion<br>**2** Se calme difficilement<br>**3** Inconsolable. Succion désespérée								
	**SCORE TOTAL**								
**OBSERVATIONS**									

DEBILLON T, SGAGGERO B, ZUPAN V, TRES F, MAGNY JF, BOUGUIN MA, DEHAN M. Sémiologie de la douleur chez le prématuré.Arch Pediatr 1994, 1, 1085-1092.
DEBILLON T., ZUPAN V., RAVAULT N, MAGNY J.F., DEHAN M. Development and initial validation of the EDIN scale, a new tool for assessing prolonged pain in preterm infants. Arch Did Child Neonatal Ed 2001, 85 : F36-F41.

APPENDIX 2: EVALUATION OF GENERAL MOVEMENT FROM 0 TO 2 MONTHS

General Movement Optimality Score-Revised (GMOS-R)

The GM Trust

The General Movements Optimality Score-Revised (GMOS-R)

Detailed Assessment of General Movements during Preterm and Term Age:

Christa Einspieler and Arie Bos for the GM Trust, 2024

Name		Date of Birth	
Recording Date		Postmenstrual Age	Weeks
Behavioural State (Coincidence)	☐ State 2 (Active Sleep)	☐ State 4 (Active Wakefulness)	

GM CATEGORY	☐ Normal<br>☐ Poor Repertoire<br>☐ Cramped-Synchronised<br>☐ Chaotic	SEQUENCE	☐ 2 variable<br>☐ 1 monotonous and/or incomplete sequence<br>☐ 0 synchronised<br>☐ 0 disorganised

Detailed Scoring:

	NECK	TRUNK
	☐ 2 variably involved in the sequence	☐ 2 fluent and elegant rotations
	☐ 1 isolated movements	☐ 1 repetitive or few rotations
	☐ 0 does not move at all	☐ 0 almost no rotations or en bloc movement

	UPPER EXTREMITIES	LOWER EXTREMITIES
Amplitude	☐ 2 variable<br>☐ 1 monotonous<br>☐ 0 almost always small<br>☐ 0 almost always large	☐ 2 variable<br>☐ 1 monotonous<br>☐ 0 almost always small<br>☐ 0 almost always large
Speed	☐ 2 variable<br>☐ 1 monotonous<br>☐ 0 almost always slow<br>☐ 0 almost always fast	☐ 2 variable<br>☐ 1 monotonous<br>☐ 0 almost always slow<br>☐ 0 almost always fast
Spatial range	☐ 2 full age-specific space used<br>☐ 1 limited space<br>☐ 0 in one plane only, e.g. only on surface	☐ 2 full age-specific space used<br>☐ 1 limited space<br>☐ 0 in one plane only, e.g. lifted-released
Proximal rotatory components	☐ 2 present, variable, fluent and elegant<br>☐ 1 present but monotonous<br>☐ 0 almost no rotations	☐ 2 present, variable, fluent and elegant<br>☐ 1 present but monotonous<br>☐ 0 almost no rotations
Distal rotatory components	☐ 2 present, variable, fluent and elegant<br>☐ 1 present but monotonous<br>☐ 0 almost no rotations	☐ 2 present, variable, fluent and elegant<br>☐ 1 present but monotonous<br>☐ 0 almost no rotations
Beginning	☐ 2 smooth and gradually increasing<br>☐ 1 minimal fluctuation<br>☐ 0 almost always abrupt	☐ 2 smooth and gradually increasing<br>☐ 1 minimal fluctuation<br>☐ 0 almost always abrupt
End	☐ 2 smooth and gradually decreasing<br>☐ 1 minimal fluctuation<br>☐ 0 almost always abrupt	☐ 2 smooth and gradually decreasing<br>☐ 1 minimal fluctuation<br>☐ 0 almost always abrupt
Stiffness	☐ 2 movements are smooth without stiffness<br>☐ 1 stiffness occasionally present<br>☐ 0 stiffness almost always present	☐ 2 movements are smooth without stiffness<br>☐ 1 stiffness occasionally present<br>☐ 0 stiffness almost always present

Subscore SEQUENCE (0 – 2)	
Sub Subscore NECK and TRUNK (0 – 4)	
Subscore UPPER EXTREMITIES (0 – 16)	
Subscore LOWER EXTREMITIES (0 – 16)	
Total GMOS-R (0-58)	

☐ LMIC/UMIC
☐ HIC

Age-Specific Centile Rank within GM Category	

Groupement Hospitalier de Territoire Saint-Denis Gonesse Plaine de France

École Pratique des Hautes Études | PSL

CHArt Cognitions Humaine et Artificielle

APPENDIX 2: EVALUATION OF GENERAL MOVEMENTS AT 3–5 MONTHS

Motor Optimal Score (MOS)

Assessment of Motor Repertoire - 3 to 5 Months

Christa Einspieler and Arie Bos, the GM Trust 2000

Ref: Einspieler C, Prechtl HFR, Bos AF, Ferrari F, Cioni G. Prechtl's Method of the Qualitative Assessment of General Movements in Preterm, Term and Young Infants. Clin Dev Med 167. London: MacKeith Press 2004, page 28.

Name: ....

born: .... PMA: .... BW: ....

Recording Date: .... Age: ....

Observed movement patterns:

Number (excl.fidgety mov.) [|] normal [|] abnormal

N A fidgety movements

N A swipes	N A hand-mouth contact	N A arching
N A wiggling-oscillating	N A hand-hand contact	N A rolling to side
N A kicking	N A fiddling / cloths, blanket	N A visual scanning
N A excitement bursts	N reaching	N hand regard
N A smiles	N A foot-foot contact	N A head anteflexion
N A mouth movements	A asym segm movements	A arm movements in circles
A tongue movements	N A legs lift	A absent leg movements
N A head rotation	N A hand-knee contact	A

Observed postural patterns: [|] normal [|] abnormal

N A head in midline (20 ")	N variable finger postures	A hyperextension of the neck
N A symmetrical	A predominant fisting	A hyperextension of trunk
N A spontaneous ATNR absent or could be overcome	A finger spreading	A extended arms / on / above surface
A body and limbs 'flat' on surface	A few finger postures	A extended legs
	A synchronised opening and closing	

Movement character (global score):

N smooth and fluent	A stiff	A predominantly slow speed
A jerky	A cramped-synchronized	A predominantly fast speed
A monotonous	A	A predominantly large ampl.
A tremulous		A predominantly small ampl.

Motor Optimality List:

1.	Fidgety Movements	normal	❑	12
		abnormal	❑	4
	± + ++ * **	absent / sporadic	❑	1
2.	Repertoire of co-existent other movements	age-adequate	❑	4
		reduced	❑	2
		absent	❑	1
3.	Quality of other movements	N > A	❑	4
		N = A	❑	2
		N < A	❑	1
4.	Posture	N > A	❑	4
		N = A	❑	2
		N < A	❑	1
5.	Movement character	smooth and fluent	❑	4
		abnormal but not CS	❑	2
		cramped-synchronized	❑	1

Motor Optimality Score: [ ]

from 28 to 5

APPENDIX 3: BAYLEY-4 PSYCHOMOTOR DEVELOPMENT SCALE

Bayley-4 Test: 1 to 6 months

1 month – 4 months (A–D)	1 month – 5 months (A–E)	5 months and older (E)	1 month – 15 months (A–J)

Return Policy: If the child does not earn 2 points on any of the first 3 items of a subtest, find the starting point (highlighted in gray) for the previous age and administer the items in the in which administration.

Points de départ	
Âge / Âge corrigé	Point de départ
0 mois 16 jours – 1 mois 30 jours	A
2 mois 0 jour – 2 mois 30 jours	B
3 mois 0 jour – 3 mois 30 jours	C
4 mois 0 jour – 4 mois 30 jours	D
5 mois 0 jour – 5 mois 30 jours	E
6 mois 0 jour – 6 mois 30 jours	F
7 mois 0 jour – 7 mois 30 jours	G
8 mois 0 jour – 10 mois 30 jours	H
11 mois 0 jour – 13 mois 30 jours	I
14 mois 0 jour – 16 mois 30 jours	J
17 mois 0 jour – 19 mois 30 jours	K
20 mois 0 jour – 22 mois 30 jours	L
23 mois 0 jour – 25 mois 30 jours	M
26 mois 0 jour – 28 mois 30 jours	N
29 mois 0 jour – 32 mois 30 jours	O
33 mois 0 jour – 38 mois 30 jours	P
39 mois 0 jour – 42 mois 30 jours	Q

Reminder of starting points:

Stopping rule: Stop when the The child participant scores 0 on 5 consecutive items.

COG-x: Cognitive subtest, item number x

CR-x: Receptive communication subtest

CE-x: Expressive communication subtest

FM-x: Fine motor subtest

GM-x: Gross motor subtest

I. During the test

Equipment checklist: stopwatch.

- **Calms down when held in the arms** (COG-1)

Instructions: If the child becomes increasingly agitated during the assessment or becomes irritable, ask the parent to hold the child.

Item	Materials	Scoring Criteria	Score
**Calms down when held**	Stopwatch	He **consistently** calms down **within 30 seconds** when picked up and **remains calm**.	2
		**Sometimes** calms down **within 30 seconds** or calms down **only briefly** when held but **does not remain calm**.	1
		**Does not calm down** when held.	0
		Time elapsed:	
		Comments:	
	**Question for the parent:** When your child cries and you hold him or her, does he or she calm down and stay calm almost always (2), sometimes (1), or never (0)?		☐

- **Puts hand to mouth** (FM-4)

Instructions: Observe whether the child brings their hand to their mouth.

Item	Materials	Scoring Criteria	Score
**Brings hand to mouth**	None	Tries to put a hand in the mouth and consistently succeeds.	2
		Tries to put hand in mouth but does not always succeed.	1
		Does not try to put his hand in his mouth.	0
		Comments:	
	**Question for parents:** When your child tries, is he or she able to put his or her hand in his or her mouth almost always (2), sometimes (1), or never (0)?		☐

- **Keeps hands open** (FM-8)

Instructions: Observe whether one or both hands are open or whether the fist is clenched when not performing a task.

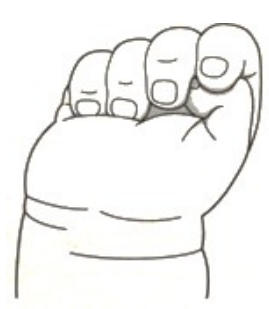

If the fist is clenched, check whether the thumb is pressed against the palm and covered by the other fingers (*cortical thumb position*).

Item	Materials	Scoring Criteria	Score
**Keeps hands open**	None	Keeps both hands open almost all the time.	2
		Keeps both hands open sometimes.	1
		One or both hands are clenched into a fist almost all the time, or the thumb is pressed against the palm and covered by the other fingers (cortical thumb position) at least some of the time.	0
		Comments:	
	**Question for the parent:** When your child is not holding anything, are their hands open almost all the time (2), sometimes (1), or never (0)?		☐

II. Position – Prone

1 month – 4 months (A–D)	1 month – 5 months (A–E)	5 months and older (E)	1 month – 15 months (A–J)

Equipment checklist: stopwatch, bell, rattle, attention-grabbing object.

- **Turns head to both sides** (GM-3)

Position: Prone.

Instructions: Observe whether the child is able to lift the head and turn it to each side (that is, by lifting the head high enough above the examination surface to clear the nose).

If this behavior is not observed, draw the child's attention to an object. For example, wave the rattle on either side of the child's head.

Item	Materials	Scoring Criteria	Score
**Turns head to both sides**	Eye-Catching Item	Turns its head from side to side, **in both directions**	2
		Turns head from side to side, **on one side only**.	1
		Does not turn head from side to side, regardless of which side.	0

		Comments:

○ **Head Control: Prone Suspension** (GM-5) Position: Prone

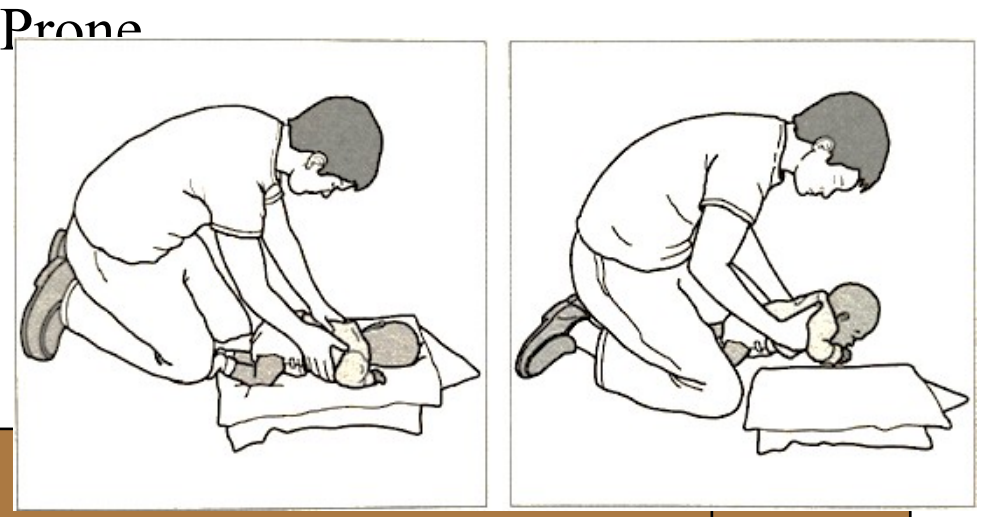

Instructions: Place both hands under the child so that your thumbs are behind the child's armpits. Gently lift the child and hold him or her suspended in a prone position.

Item	Equipment	Scoring Criteria	Score
**Head control: suspended in prone position**	None	Holds the head in **a midline position** or **lifts** the head **slightly**.	2
		Holds the head **briefly** before letting it fall forward.	1
		Does not hold the head in a neutral position even briefly or lets the head fall forward when lifted.	0
		Comments:	

○ **Head control in the prone position: 45°** (GM-7)

Position: Prone.

Instructions: When the child is lying on his or her stomach on the examination surface, observe whether the child lifts the head above the surface or raises the upper body using the arms.

If this behavior is not observed, stand facing the child and hold an object about 30 cm above the examination surface, or speak to the child to encourage him or her to lift the head.

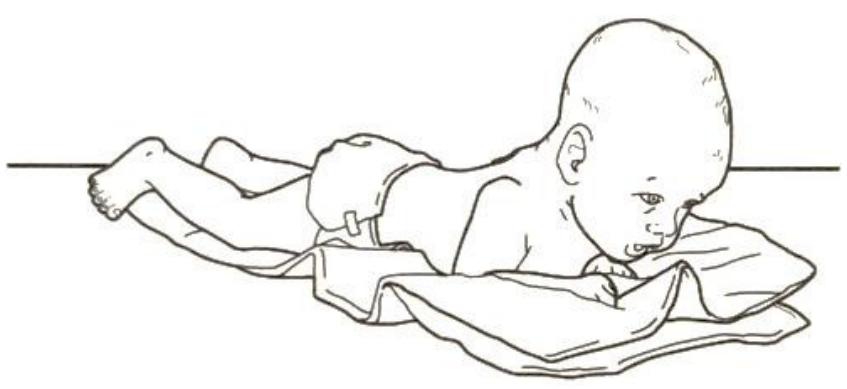

Item	Materials	Scoring Criteria	Score
**Controls their head in supine prone: 45°**	Stopwatch, an attractive	Holds head raised at a 45° angle for **at least 3 seconds**.	2
		Holds head raised at a 45° angle for **less than 3 seconds**.	1
		Does not raise the head to at least 45°.	0
		Elapsed time:	
		Comments:	

- Upper Body Lift in Prone Position (GM-11)

Position: Prone.

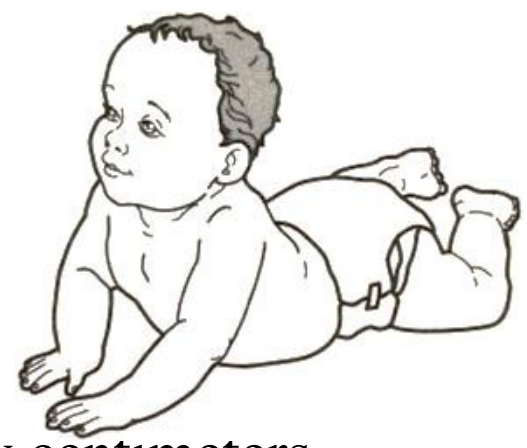

Instructions: Observe whether the child pushes upward using both arms, so that the head and upper body rise above the examination surface. The hips, abdomen, and upper legs remain on the examination surface.

If this behavior is not observed, stand near the child's head and hold an object a few centimeters above the examination surface to encourage the child to lift themselves up.

Item	Equipment	Scoring Criteria	Score
**Lifts the upper body while in the prone position**	Attractive object	Lift the head and upper body by pushing **with both hands.**	2
		Raise the head and upper body by pushing on the **elbows** or **forearms**.	1
		Does not lift the head and upper body, or **use their legs** to lift their abdomen.	0
		Comments:	

- Rolls from stomach to back (GM-10)

Position: Prone.

Instructions: Observe whether the child rolls from the stomach to the back, rolling to the right or to the left. If this behavior is not observed, shake the bell or rattle on one side.

If the child does not respond by rolling onto their back, repeat the procedure on the other side.

Item	Materials	Scoring Criteria	Score
Rolls from stomach to back	Bell, rattle	Is consistently able to roll from stomach to back, rolling to the right or to the left.	2
		Is able to roll from stomach to back by rolling to the right or left, but **not consistently**, or rolls from stomach **to side**.	1
		Does not roll from their stomach to their back.	0
		Comments:	
		**Question for parents**: Does your child manage to roll from their stomach to their back almost always (2), sometimes (1), or never (0)?	☐

III. Position – Supine

1 month – 4 months (A–D)	1 month – 5 months (A–E)	5 months and older (E)	1 month – 15 months (A–J)

Equipment checklist: stopwatch, ring with string, small ball, spoon, rattle, bell, pencil

- **Spreads arms and extends legs** (GM-1)

Position: Supine.

Instructions: Observe the child's arm and leg movements.

Item	Materials	Scoring Criteria	Score
**Spreads their arms and extends their legs**	None	Spreads arms and extends legs at least once, in a **fluid and coordinated** manner.	2
		Spreads arms and extends legs, but the movements are neither fluid nor coordinated.	1
		Shows **asymmetry** in movements, spreading out only on one side or not moving the arms and legs at the same time.	0
		Comments:	

- [Asymmetric tonic neck reflex](#) (GM-12)

<u>Position</u>: Supine.

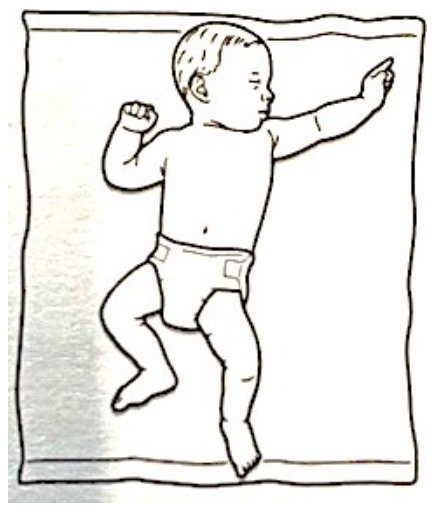

<u>Instructions</u>: Observe the child while lying on his or her back. Check to see if the child assumes the “fencer’s position,” with the head turned to one side and the arm on the same side extended outward.

The opposite arm should be flexed at an angle of 45° to 90°. The leg on the side of the face (toward which the head is turned) should be extended, and the other leg should be bent.

Item	Equipment	Scoring Criteria	Score
**Asymmetric tonic neck reflex**	None	No spontaneous asymmetric tonic neck posture.	2
		Transient spontaneous tonic neck posture, mostly absent.	1
		Frequently exhibits a tonic neck posture while lying on the back.	0
		Comments:	

- **[Recognizes the parent](#)** (COG-5 and CR-4)

<u>Position</u>: Supine, head slightly supported.

<u>Instructions</u>: Attract the child’s attention by interacting with them, then step out of their field of vision.

Without taking your eyes off the child’s face, ask the parent to step into the child’s field of vision.

Observe whether the child recognizes the parent (smiles, looks at them intentionally, makes sounds, or displays an animated expression and then pays attention to the parent).

Administer 3 times, if necessary.

Item	Materials	Scoring Criteria	Score
**Recognizes the parent**	None	The child’s expression changes **immediately,** indicating that he or she recognizes the parent **on at least one occasion**.	2

		Shows a **brief** and **slight** change in facial expression, or the reaction is **not immediate**.	1
		Shows **no change in facial expression**.	0
		Comments:	

- **Responds to parent** (COG-6 and CR-6) Position: Supine

Instructions: Ask the parent to stand at the child's feet and lean over the child so that their hands are positioned as if to lift the child. After a brief pause, the parent should place their hands under the child's arms, as if to lift the child, but without actually lifting them.

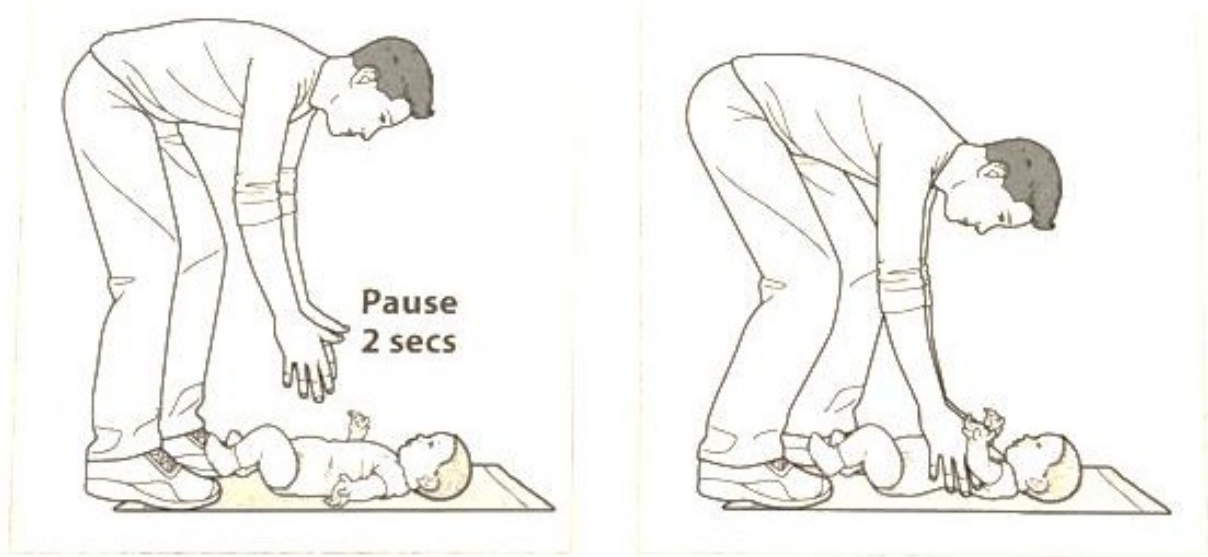

Observe whether the child exhibits an anticipatory reaction (increased motor activity such as extending their arms, kicking their feet, rapid breathing, vocalizing, or a brightening facial expression).

Item	Materials	Scoring Criteria	Score
**Reacts to the parent**	None	Shows a **clear anticipatory reaction**.	2
		Displays a reaction **that does not appear to be related to or reflect anticipation**.	1
		Shows **no reaction**.	0
		Comments:	
	**Question for the parent:** When you are about to pick up your child, does he or she show anticipation of being picked up almost always (2), sometimes (1), or never (0)?		☐

- **Reacts to the parent's departure** (COG-7)

Position: Supine, head slightly supported on the examiner's lap.

Instructions: Ask the parent to position themselves within the child's field of vision and interact with the child. When the child looks at the parent's face, ask the parent to quickly leave the child's field of vision, ceasing interaction.

Observe the child's facial expression throughout this process.

Item	Materials	Scoring Criteria	Score
**Reacts to the parent's departure**	None	**Changes facial expression** or **clearly reacts** in some other way when the parent leaves (*stops all activity, frowns, or shows other signs of facial expression, makes a rudimentary search, etc.*).	2
		Responds **briefly** or **ambiguously**.	1
		**Shows no signs of a reaction**.	0
		Comments:	

- **Looks at an object** (COG-2)

Position: Supine, cradled, or supported sitting position.

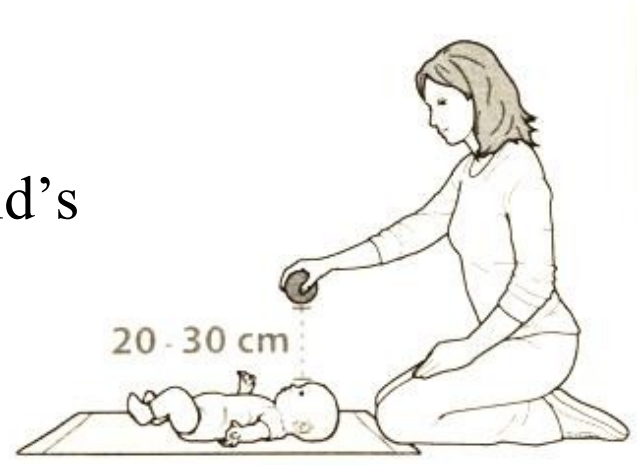

Instructions: Show the object to the child, holding it 20 to 30 cm from the child's face. Move the object slightly to attract the child's attention. Try with a second object if necessary. Then note the elapsed time.

Item	Materials	Scoring Criteria	Score
**Looks at an object**	Ring with cord, small ball, spoon, other small, attractive object, stopwatch	Look at the object continuously for **7 to 10 s**.	2
		Look at the object **continuously** for **3 to 6 s** or look at the object **intermittently** for **3 to 6 s**.	1
		Look at the object continuously **for 0 to 2 s** or **do not look at the object**.	0
		Elapsed time:	
		Comments:	

- [**Getting used to the rattle**](#)

(COG-3) Position: Supine.

Instructions: When the child is calm, hold the rattle 20–30 cm from the right side of the child's head. Shake the rattle for 3 seconds to elicit a head-turning response toward the sound source (a change indicated either by a movement of a certain amplitude or by an increased level of alertness), then pause for 5 seconds. This constitutes one trial. If the child exhibits a head-turning response to the right, repeat the trial 4 times in the same manner.

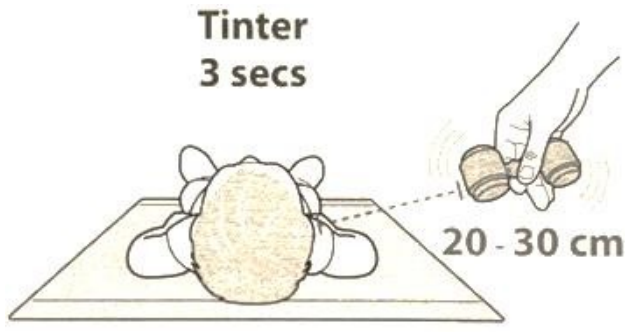

If the child does not exhibit a head-tilt response to the right, repeat the procedure on the **left** side (1 initial trial, followed by 4 additional trials, if a head-tilt response toward the sound source is observed on the 1st trial).

If the child does not show a head-turning response to the left, do not persist and move on to the next item.

Administer the COG-4 item immediately after administering this item.

Item	Materials	Scoring Criteria	Score
**Becomes accustomed to the rattle**	Rattle	Shows **signs of habituation** (reduced orienting response) during the **first 3 trials** on the right or left side.	2
		Shows **signs of habituation** during **the 4th or 5th trial** on the right or left side.	1
		Shows **no orienting response** or **no signs of habituation during the 5 trials**, on side or the other.	0
		Comments:	

- [**Responds to novelty**](#) (COG-4)

Note: *If the child did not demonstrate an orienting response during item COG-3 and is getting used to the rattle, score this item as 0 and proceed to item COG-5. Recognizes the parent.*

Position: Supine.

Instructions: Hold the bell 30–40 cm from the child's head, on the side that elicited an orienting response during item COG-3. When the child is calm, gently ring the bell twice.

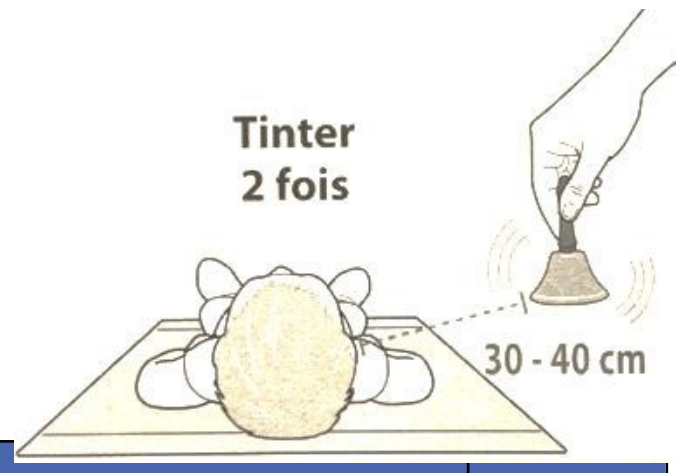

Record the elapsed time.

Item	Materials	Scoring Criteria	Score
**Reacts to something new**	Bell, stopwatch	Responds to the bell by exhibiting a marked change in behavior **within 5 seconds**.	2
		Reacts to the bell by exhibiting a but **is brief** or **does not occur within 5 seconds**.	1
		**Does not react to the bell** (no sign of a change).	0
		Time elapsed:	
		Comments:	

- **Follow the pencil: horizontal plane** (FM-7)

Position: Supine or supported sitting.

Instructions: Hold the pencil by one end, positioning it approximately 20–30 cm from the child's face and parallel to the axis of their body. Ensure that the child is looking at the pencil and move the pencil horizontally over a distance of approximately 15 cm, from the midline toward the child's right side, then toward the left, and back to the midline.

This constitutes one trial.

If the child stops following the pencil, draw their gaze back to the pencil before continuing the test.

Conduct up to three trials if necessary.

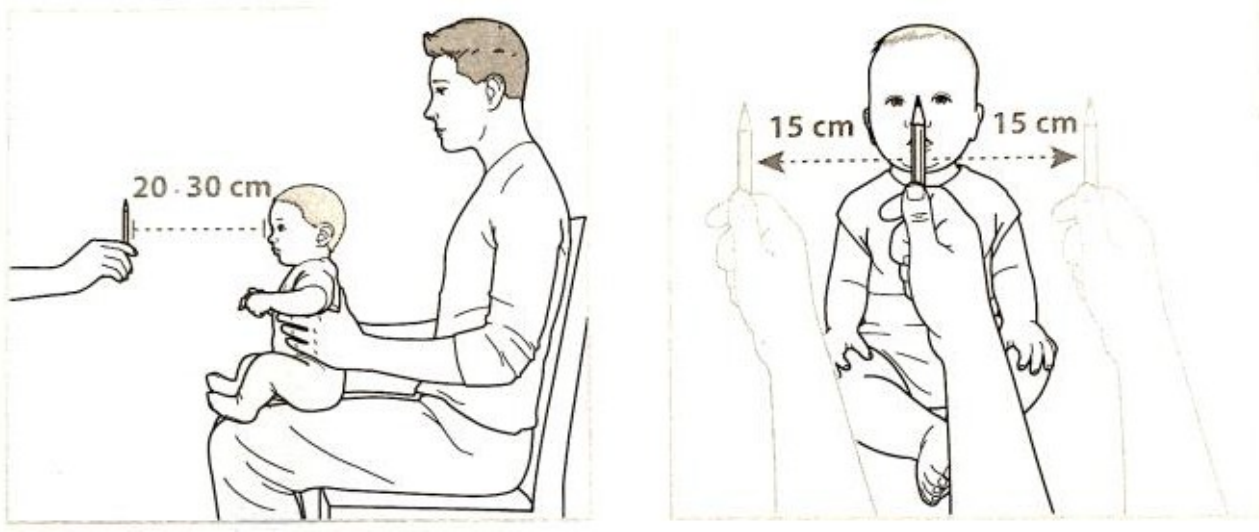

Item	Materials	Scoring Criteria	Score
**Follows the pencil: horizontal plane**	Pencil	The eyes follow the pencil throughout a drawing.	2
		The eyes follow the pencil, but not throughout the entire test.	1
		The eyes do not follow the pencil.	0
		Comments:	

- **Eyes Follow the Ring: Horizontal Plane** (FM-2)

Position: Supine.

Instructions: Suspend the ring by its string so that the lower edge of the ring is at the child's midline and approximately 20 to 30 cm from the child's face. Move the ring slightly to attract the child's attention. Gently move the ring (at a rate of 30 cm every 3–4 seconds) in a horizontal plane 15 cm from the child's midline to the right, then to the left, and back to the midline.

This constitutes one trial.

If the child takes their eyes off the ring, draw their gaze back it before continuing the trial.

Conduct up to three trials if necessary.

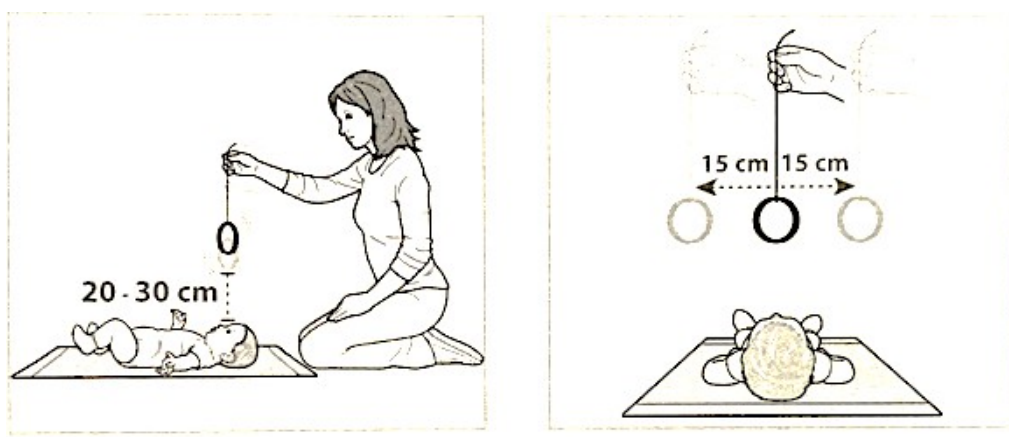

Item	Materials	Scoring Criteria	Score
**Eyes follow the ring: horizontal plane**	Ring with cord	The eyes follow the ring throughout a trial.	2
		The eyes follow the ring, but not throughout the entire trial.	1
		The eyes do not follow the ring.	0

		Comments:

- **Eyes Follow the Ring: Vertical Plane** (FM-3)

Position: Supine.

Instructions: Suspend the ring by its string so that the lower edge of the ring is level with the child's midline and approximately 20 to 30 cm from the child's face. Move the ring slightly to attract the child's attention. Gently move the ring (at a rate of 30 cm every 3–4 seconds) in a vertical plane along the midline over a distance of 15 cm, moving from the eyes toward the forehead, then toward the chest for about 15 cm, and then back to eye level.

This constitutes one trial.

If the child takes their eyes off the ring, draw their gaze back to it before continuing the trial.

Conduct up to three trials if necessary.

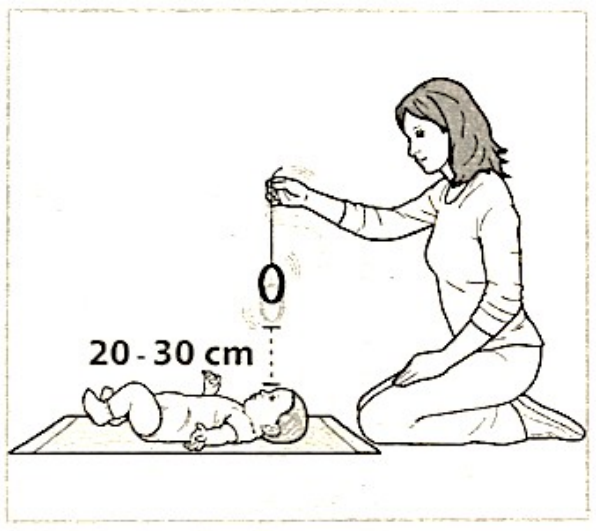

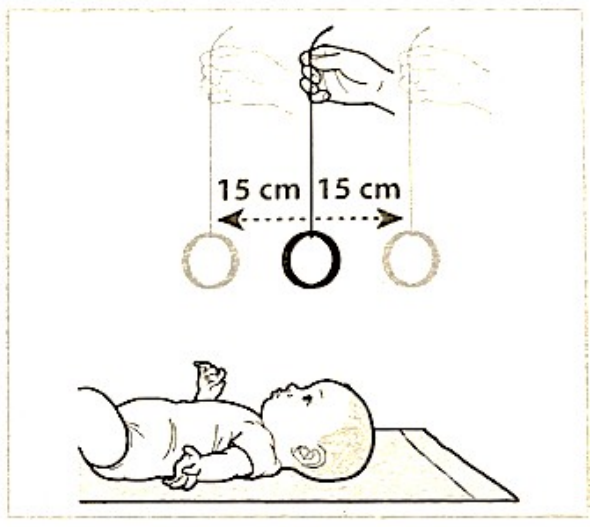

Item	Materials	Scoring Criteria	Score
**Eyes follow the ring: vertical plane**	Ring with cord	The eyes follow the ring throughout a trial.	2
		The eyes follow the ring, but not throughout the entire trial.	1
		The eyes do not follow the ring.	0
		Comments:	

- **Holds the ring** (FM-5)

Position: Supine.

Instructions: Place the ring in the child's hand. If the child has a clenched fist, gently open their fist or ask a parent to do so, then place the ring in the child's hand.

Item	Materials	Scoring Criteria	Score
**Holds the ring**	Stopwatch, ring with cord	Holds the ring for **at least 5 seconds**.	2
		Holds the ring for **2 to 4 seconds**.	1
		Holds the ring for **0 to 1 seconds**.	0
		Time elapsed:	
		Comments:	
	**Question for parents:** When your child has something in their hand, are they able to hold it for at least 5 seconds almost always (2), sometimes (1), or never (0)?		☐

IV. Position – Dorsal Proclive

1 month – 4 months (A–D)	1 month – 5 months (A–E)	From 5 months (E)	1 month – 15 months (A–J)

Equipment checklist: soft duck toy, rattle, bell.

○ **Briefly focuses attention on a person)** (CR-1) Position: Supine or rocking position.

Instructions: Observe how the child's attention or interest manifests itself—does the child look toward the source of stimulation? If these behaviors are not observed during indirect observation, ask the parent to try to elicit these sounds using the following methods:

- Cuddle up close to the child while talking to them
- Vary the quality of the voice by changing the pitch, volume, or duration of the sounds

- Add an exclamation or expressive sounds while speaking
- Gently pat the child while speaking or singing softly
- Gently stroke or tickle the child

Item	Materials	Rating Criteria	Score
**Briefly focuses their attention on a person**	None	**Unfailingly** looks at the person and maintains eye contact for at least 3 seconds.	2
		**Briefly** looks at the person for less than 3 seconds.	1
		Don't look at the person.	0
		Comments:	

- **The eyes follow a moving person (ocular tracking)** (FM-1) Position: Supine.

Instructions: Ask the parent to stand at the child's feet. Ask them to walk across the child's visual field, moving from the child's left to their right, then from their right to their left.

This is a trial. Ensure that the parent remains within the child's visual field. Administer up to two trials if necessary.

Item	Materials	Scoring Criteria	Score
**The eyes follow a moving person (ocular tracking)**	None	The eyes follow the person to the left and right, crossing the midline.	2
		The eyes briefly follow the person, but without crossing the midline.	1
		The eyes do not follow the person, or the child is looking at something else in the room.	0
		Comments:	

- **Head control: dorsal suspension** (GM-4)

Position: Dorsal proclive.

Instructions: With the child held in a supine prone position, carefully remove your hand from the back of the child's head, taking into account the child's ability to maintain head control, and ensure that the child's head does not fall backward.

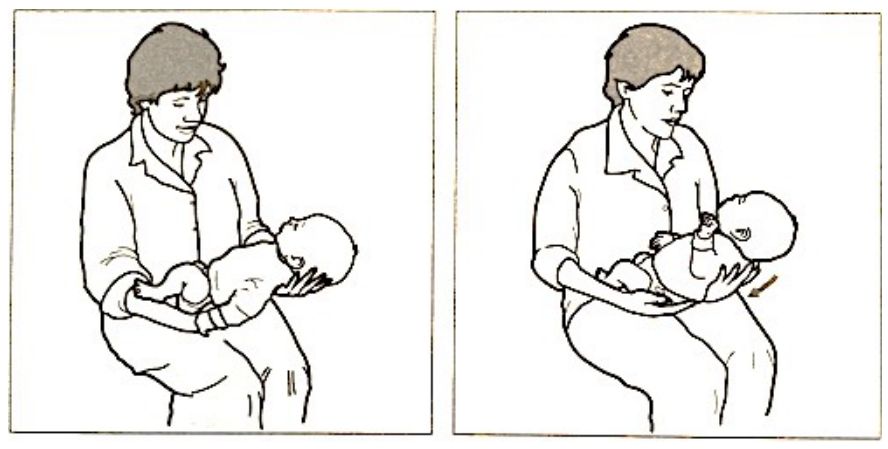

Item	Equipment	Scoring Criteria	Score
**Controls their head: dorsal suspension**	None	Holds the head in a midline position or lifts it slightly<br><br>the head.	2
		Briefly holds the head in a midline position before it falls backward.	1
		Does not keep the head in the midline position, even briefly. Support is constantly needed.	0
		Comments:	

- Calms down when spoken to (CR-2)

Position: Supine.

Instructions: When the child is agitated or fussy, observe whether he or she calms down when spoken to. Avoid touching or picking up the child.

Item	Materials	Scoring Criteria	Score
**Calms down when spoken to**	None	Calms down **without fail** and remains calm when spoken to.	2
		**Sometimes** calms down or calms down **briefly** when spoken to.	1
		Does not calm down when spoken to.	0
		Comments:	

- Undifferentiated vocalizations (CE-1)

Position: Supine or rocking position

Instructions: Observe whether the child spontaneously produces guttural sounds, grunts, chirps, or high-pitched cries. If these behaviors are not observed during indirect observation, ask the parent to try to elicit these sounds using the following methods:

- Cuddle up close to the child while talking to them
- Vary the way you speak by changing your voice's pitch (high, medium, low), volume, or the duration of the sounds
- Gently pat the child while speaking or singing softly

If the child's vocalizations change in pitch (high, medium, low) or show variations in intonation, award 2 points.

Item	Materials	Scoring Criteria	Score
Undifferentiated vocal exercises	None	**Frequently** produces guttural sounds, high-pitched cries, growls, or chirps.	2
		**Occasionally** produces guttural sounds, high-pitched cries, growls, or chirps.	1
		Does not produce any sounds.	0
		Comments:	
	**Question for the parent:** When you play and talk with your child, does he or she make guttural sounds, grunts, chirps, or high-pitched cries—often (2), not often (1), or not at all (0)?		☐

- Social Smiling (CE-2)

Position: Supine or rocking position

Instructions: Observe whether the child responds to the attention being given to them. For example, do they smile or laugh? If these behaviors are not observed during indirect observation, ask the parent to try to elicit them from the child using the following methods:

- Cuddle up close to the child while talking to them
- Vary the way you speak by changing your voice's pitch (high, medium, low), volume, or the duration of the sounds
- Gently pat the child while speaking to them or singing softly

Focus on the child's smile.

Item	Materials	Rating Criteria	Score
Social smile	None	Smiles **frequently** in response to attention or interactions.	2
		Smiles **occasionally** in response to attention or interactions.	1
		Does not smile in response to attention or interactions, or cries.	0
		Comments:	
	**Question for the parent:** When you or other adults look at your child, or when you cuddle with him or her, does he or she smile or laugh often (2), not often (1), or not at all (0)?		☐

- Expresses mood vocally (CE-3)

Position: Supine or rocking position

Instructions: Observe whether the child spontaneously produces expressive vocalizations reflecting the following states:

- Pleasure during toy-based activities, free play, or social interactions
- Displeasure (without crying) when play is interrupted, when toys are taken away, or when the child is restrained or secured
- Enthusiasm or impatience when trying to touch or grab a toy that is out of reach
- Satisfaction when he manages to grab a toy or solve a problem

Item	Materials	Rating Criteria	Score
Expresses his mood vocally	None	Produces **frequent** and **varied** vocalizations that express its mood.	2
		Makes **occasional** vocalizations that express its mood.	1
		Does not make vocalizations that express its mood or cry.	0
		Comments:	
	**Question for the parent:** Does your child make sounds other than crying to show you how he or she feels when happy or sad, such as laughing or whining, often (2), not often (1), or not at all (0)?		☐

- Social vocalizations and laughter (CE-4)

Position: Supine or rocking position

Instructions: Observe whether the child responds to the attention given to them. For example, do they laugh or produce vocalizations (giggles, coos, high-pitched cries)? If these behaviors are not observed during indirect observation, ask the parent to try to elicit these sounds using the following methods:

- Cuddle up close to the child while talking to them
- Vary the way you speak by changing your voice's pitch (high, medium, low), volume, or the duration of the sounds
- Gently pat the child while speaking or singing softly

Focus on the child's vocalizations, not just on their smiles.

Item	Materials	Rating Criteria	Score
Social vocalizations and laughter	None	**Frequently** laughs and makes vocalizations in response to attention or interactions.	2
		**Occasionally** laughs and makes vocalizations in response to attention or interactions.	1
		The child only smiles, does not make any vocalizations, or cries.	0
		Comments:	
	**Question for the parent:** Does your child often (2), not often (1), or never (0) make sounds or laugh when you talk to him or when you cuddle with him?		☐

- Vowel sounds (CE-5)

Position: Supine or cradled position

Instructions: Observe whether the child spontaneously makes vowel sounds, such as: "ah" / "uh" / "oh" / "eh."

If these vowel sounds are not observed during indirect observation, ask the parent to try to elicit them from the child using the following methods:

- Cuddle up close to the child while talking to them

- Vary the way you speak by changing your voice's pitch (high, medium, low), volume, or the duration of the sounds
- Gently pat the child while speaking or singing softly

Item	Materials	Rating Criteria	Score
Vowel sounds	None	**Frequently** produces a variety of distinct vowel sounds.	2
		**Occasionally** produces vocalizations consisting of distinct vowel sounds.	1
		Does not produce vocalizations consisting of distinct vowel sounds.	0
		Comments:	
	**Question for the parent:** When your child makes sounds, do you hear them say *"ah," "uh," "oh,"* or *"eh"* often (2), not often (1), or not at all (0)?		☐

- **Reacts to sounds in the environment** (CR-3)

Position: Supine.

Instructions: Observe whether the child reacts to surrounding sounds (whether they are startled, turn their head, shift their gaze, change their activity level, show a change in facial expression, or vocalize). If these behaviors are not observed during indirect observation, stand next to the child outside their field of vision and squeeze the soft rubber duck or shake the rattle.

Item	Materials	Scoring Criteria	Score
**Responds to environmental sounds**	Soft duck rattle	Consistently demonstrates a clear reaction to sounds.	2
		The reaction to sounds is **ambiguous** or **brief**.	1
		Does not react to sounds.	0
		Comments:	

- **Responds to a person's voice** (CR-5)

Position: Supine.

Instructions: Observe whether the child reacts to a voice (whether they are surprised, turn their head, shift their gaze, change their activity level, show a change in facial expression, or vocalize). If these behaviors are not observed during indirect observation, ask the parent to stand next to the child, within the child's peripheral field of vision, and call the child by name in a normal voice.

Item	Materials	Scoring Criteria	Score
**Responds to a person's voice**	None	Unfailingly exhibits a clear reaction to the voice.	2
		The reaction to the voice is **ambiguous** or **brief**.	1
		Does not react to the voice.	0
		Comments:	
	**Question for parents**: Does your child react to your voice by turning their head or changing their facial expression almost always (2), sometimes (1), or never (0)?		☐

- **Shifts attention** (CR-7 and COG-8)

Position: Prone.

Instructions: When the child is calm and attentive, hold the bell in one hand and the rattle in the other, about 20 cm apart and 20–30 cm above the child's eyes, but within their field of vision. Gently shake one of the objects so that it produces a soft but audible sound. Wait 3–4 seconds, then shake the other object.

This constitutes one trial. Then conduct two additional trials in the same manner.

Item	Materials	Scoring Criteria	Score
**Shifts focus**	Bell, rattle	Shifts visual attention from one object to another during the **3 trials**.	2
		Shifts visual attention from one object to another during **1 or 2 trials**.	1
		Does not shift visual attention to the presented objects.	0
		Comments:	

- **Discriminates sounds** (CR-8) Position: Supine or sitting.

Instructions: Stand next to the child, out of their field of vision. Squeeze the soft duck about 10 cm behind their left ear. Continue until the child no longer tries to locate the source of the sound or no longer pays attention to it (shows no further interest).

Then gently shake the rattle, roughly in the same spot as the soft duck, and observe whether the child reacts to the sound (whether they are surprised, turn their head, shift their gaze, change their activity level, show a change in facial expression, or vocalize).

Item	Materials	Scoring Criteria	Score
**Discriminates between sounds**	Soft duck rattle	Consistently demonstrates a clear reaction to sounds.	2
		The reaction to sounds is **ambiguous** or **brief**.	1
		Does not react to sounds.	0
		Comments:	

- **Explores an object** (CR-9) Position: Prone.

Instructions: Place the rattle in the child's hand.

Item	Materials	Scoring Criteria	Score
**Explores an object**	Rattle	Shows interest in the sight, sound, and feel of the rattle by touching it, shaking it, or engaging in any playful activity.	2
		Plays with the rattle for a very short time or simply puts it in their mouth.	1
		Holds the rattle but does not interact with it.	0
		Comments:	

V. Upright position

1 month – 4 months (A–D)	1 month – 5 months (A–E)	5 months and up (E)	1 month – 15 months (A–J)

Equipment checklist: No equipment required.

- Head Upright

(GM-8) Position: Upright position

Instructions: Hold the child in an upright position above the examination surface. Slowly tilt the child’s body 45° forward, then return to the upright position.

Pause, then slowly tilt the child 45° backward, and return to the upright position. Tilt the child’s body 45° to the left, then 45° to the right, always returning to the upright position and pausing for a few seconds before tilting in a new direction.

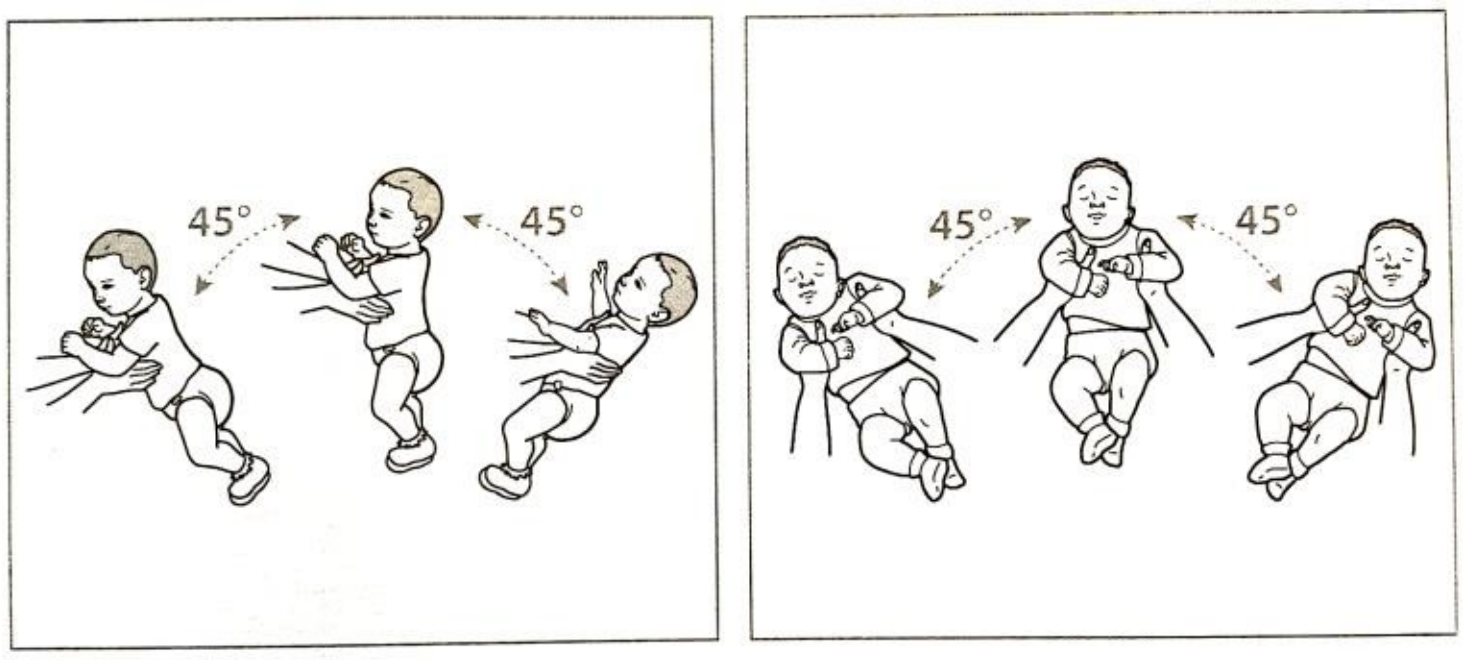

Item	Equipment	Scoring Criteria	Score
Head straight	None	Consistently maintains head balance and keeps the head in the same plane as the body. The child may overcompensate by tilting the head toward the vertical.	2
		Briefly maintains head balance, but not in all four planes; head balance is not constant.	1
		The head position is not adjusted when the child is tilted; head control is unstable.	0
		Comments:	

Descending Parachute (GM-9)

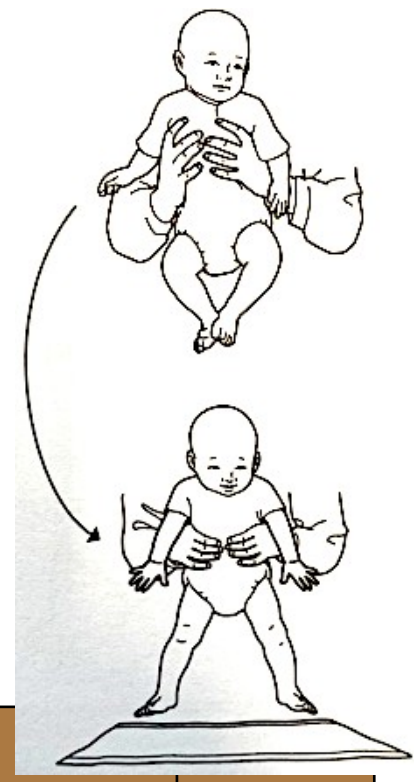

Position: Upright Position

Instructions: Hold the child under the arms in an upright position, standing behind him or her. Drop the child downward abruptly and with enough force to elicit a reaction, stopping before the child's feet touch the examination surface.

Observe whether a protective reaction is present (e.g., extension of the legs and feet turned slightly outward).

Item	Equipment	Scoring Criteria	Score
Parachute descending	None	Extend the legs and rotate the legs and feet outward symmetrically.	2
		Only partial extension of the legs, with slight symmetrical outward rotation.	1
		Keeps legs bent, asymmetric response, or scissor legs (crossed).	0
		Comments:	

VI. Position – Supported on the shoulder

1 month – 4 months (A–D)	1 month – 5 months (A–E)	Starting at 5 months (E)	1 month – 15 months (A–J)

Equipment checklist: stopwatch.

- **Holds head upright: 3 seconds** (GM-2) Position: Held against the parent's shoulder

Instructions: Ask the parent to hold the child in an upright position, with the child's head resting on the parent's shoulder, while supporting the child's head. Ask the parent to remove their hand from the child's head based on the child's ability to lift their head off the shoulder and keep it upright.

Record the elapsed time.

Item	Equipment	Scoring Criteria	Score
**Check head in a upright position: 3 s**	Stopwatch	Holds head upright without support for **at least 3 seconds**.	2
		Lifts the head without support **intermittently** for **1 to 2 seconds**.	1
		Does not lift the head	0
		Time elapsed:	
		Comments:	

- **Holds head upright: 15 seconds** (GM-6) Position: Held against the parent's shoulder

2180 Instructions: Ask the parent to hold the child in an upright position, with the child's head resting on the parent's shoulder, while supporting the child's head. Ask the parent to remove their hand from the child's head based on the child's ability to lift their head off the parent's shoulder and keep it upright. Record the elapsed time.

Item	Equipment	Scoring Criteria	Score
**Holds head in an upright position: 15 s**	Stopwatch	Holds head up, straight, and unsupported for **at least 15 s**.	2
		Holds head up, straight, and unsupported for **10 to 14 seconds**.	1
		Holds head up, straight, and unsupported for **0 to 9 seconds**.	0
		Time elapsed:	
		Comments:	

2185

VII. Position – Supported Sitting VIII.

1 month – 4 months (A–D)	1 month – 5 months (A–E)	5 months and older (E)	1 month – 15 months (A–J)

Equipment checklist: ring with string, attractive object, stimulus booklet, stopwatch

- **Head follows the ring** (FM-6)

Position: Supported sitting

Instructions: Hang the ring by its string so that the bottom edge is at the child's midline, at eye level, and approximately 20–30 cm from the child's head. Gently move the ring to attract the child's attention. Gently move the ring (at a rate of 30 cm every 3–4 seconds) in a horizontal plane from the midline toward the right ear, then toward the left ear, and then back to the midline.

This constitutes one trial. Conduct up to 3 trials if necessary.

Item	Materials	Scoring Criteria	Score
**The head follows the ring**	Ring with cord	The head follows the ring **throughout one test**.	2
		The head follows the ring, but not for the entire duration of a trial.	1
		The head does not follow the ring.	0
		Comments:	

- **Seeks attention or interaction** (CE-6) Position: Supported sitting

Instructions: Observe whether the child tries to get others' attention. If this behavior is not observed, engage in a brief interaction with the child, then turn away. Observe the child in your peripheral vision to see if they try to get your attention by vocalizing, gesturing, reaching out toward you, or grabbing you or a parent.

Item	Materials	Scoring Criteria	Score
**Seeks attention or interaction**	None	**Frequent** attempts to attract attention.	2
		**Occasional** attempts to attract attention.	1
		Does not seek attention.	0
		Comments:	

	**Question for the parent:** When you aren't paying attention to your child, does he or she try to get your attention often (2), not often (1), or not at all (0)?	☐

- Consonant Sounds

(Kindergarten 7) Position: Supported sitting

Instructions: Observe whether the child spontaneously produces consonant sounds, such as: "ba" / "da" / "di" / "ga."

If these consonant sounds are not observed during the indirect observation, ask the parent to try to elicit them from the child using the following methods:

- Tap the table with the rattle while saying "bou-bou-bou" or "pa-pa-pa"
- Gently tickle the child while saying "da-da-da" or "ta-ta-ta"
- Tap the table with your hand while saying "ma-ma-ma"

Focus on the child's vocalizations, not just on their smiles.

Item	Materials	Scoring Criteria	Score
Consonant sounds	Attractive object	**Frequently** produces a variety of vocalizations consisting of distinct consonantal sounds.	2
		**Occasionally** produces vocalizations consisting of distinct consonantal sounds.	1
		Does not produce consonantal vocalizations.	0
		Comments:	
	**Question for the parent:** When your child makes sounds, do you hear them say *"ba," "da," "di,"* or *"ga"* often (2), not often (1), or not at all (0)?		☐

- Shows a preference for visual stimuli (COG-9) Position: Supported sitting

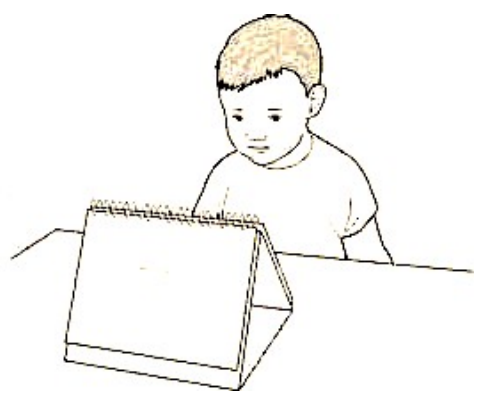

Instructions: Go to item 9 of the Cognitive Subtest in the Stimulus Booklet.

Item	Materials	Scoring Criteria	Score
		Look at the striped pattern longer **on both trials**.	2
		Look longer at the striped model with **just one attempt**.	1

**Shows a visual preference**	Stimulus booklet, stopwatch	Do not look at the striped pattern any longer, regardless of the trial.	0
		Comments:	

- [Getting used to it (balloons)](#)

(COG-10) Position: Supported sitting

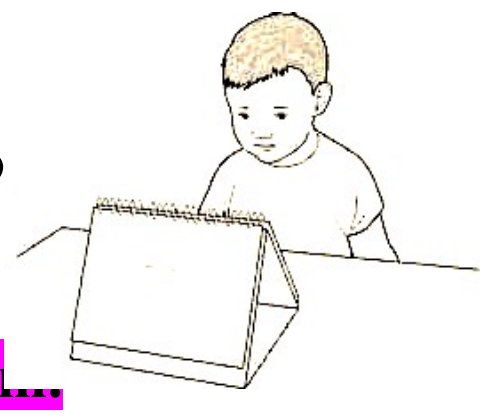

Instructions: Go to item 10 of the Cognitive Subtest in the Stimulus Booklet. Reco the elapsed time.

Administer item COG-11 (next item) immediately after administering this ite...

Item	Materials	Scoring Criteria	Score
**Habituates (balloons)**	Stimulus booklet, stopwatch	Shows interest and gets used to it within **30 seconds**.	2
		Shows interest but does not habituate within 30 seconds.	1
		Shows no interest or only glances briefly.	0
		Time elapsed:	
		Comments:	

- [Prefers novelty (ball)](#) (COG-11)

Position: Seated with support

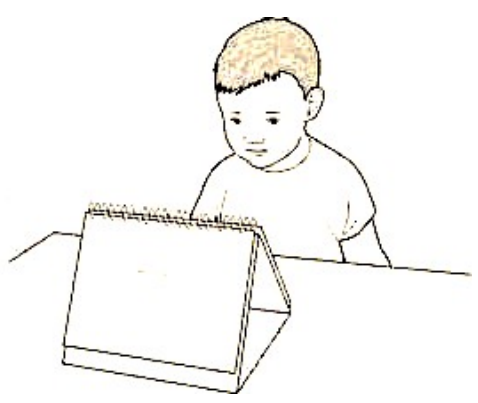

Instructions: Go to Item 11 of the Cognitive Subtest in the Stimulus Booklet.

Note: Item COG-10 (the previous item) must be completed before this item. If the child did not demonstrate an orientation response during item COG-10, assign a score of 0 to this item.

Item	Materials	Scoring Criteria	Score
Prefers novelty (ball)	Stimulus booklet, stopwatch	Looks at the ball longer than at the balloons in **both trials**.	2
		Looks at the ball longer than at the balloons in **the single-trial condition**.	1
		Do not look at the ball any longer than at the balloons, regardless of the trial.	0
		Comments:	

APPENDIX 4: CRYING DIARY – 7 DAYS

NEOTHERM: Diary of Tears

Instructions

This is a log of your baby's behavior over 7 consecutive days.

Each day is represented by **4 horizontal bars** (night/morning/afternoon, and evening), divided into boxes according to the following layout:

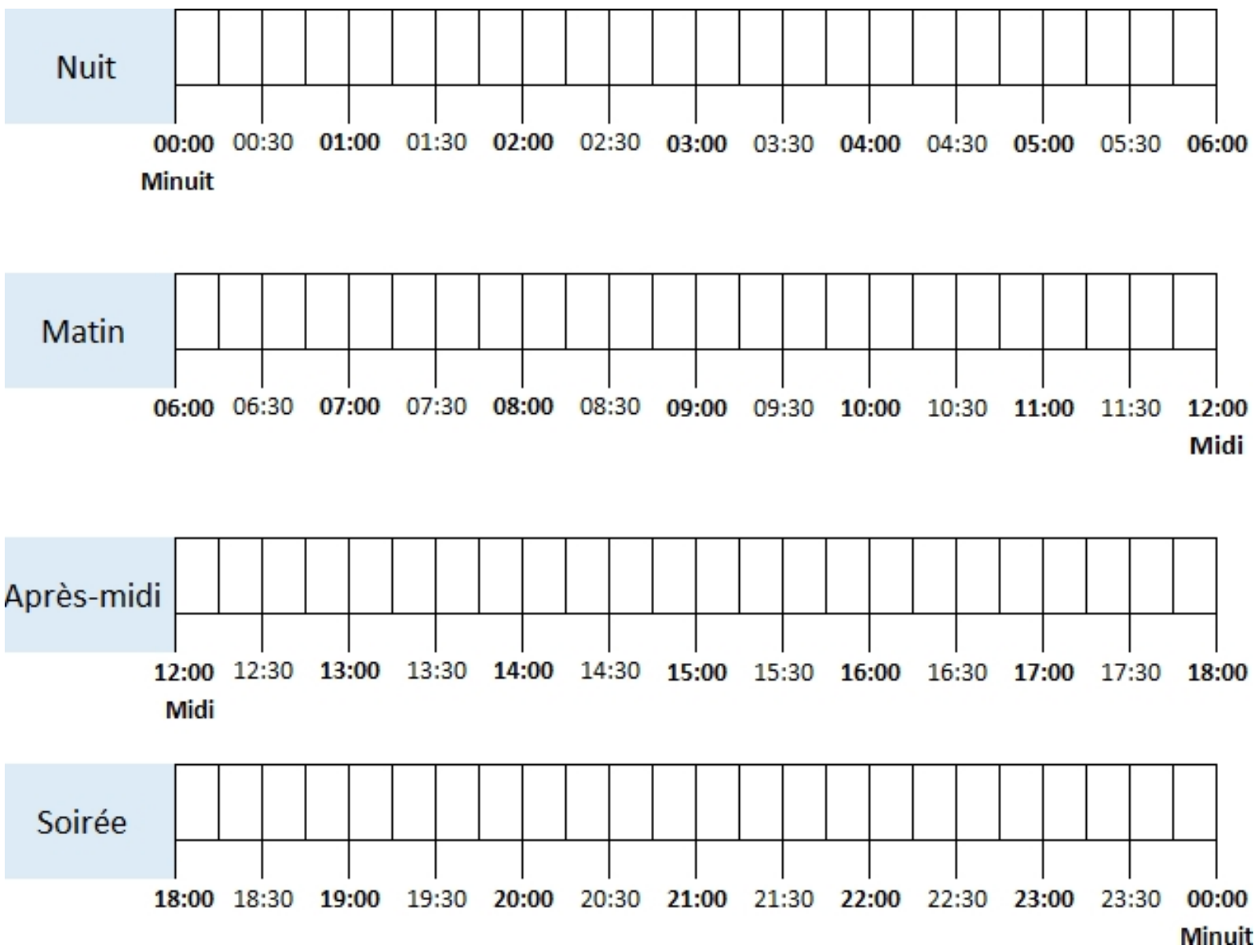

Each **box** represents **15 minutes** (4 boxes per hour), and

the time is indicated below the horizontal bar.

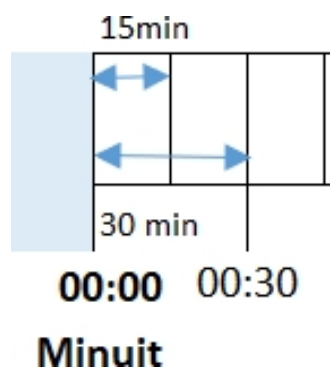

How to fill out this log:

1. **Fill in each box for the day based on your baby's main behavior during the corresponding 15-minute period:**

P: crying (your baby cries with every breath and seems very uncomfortable; it's hard to soothe them)

A : restlessness (your baby is restless, seems upset, moves around a lot, may make some noises or whimper but isn't crying; your baby is awake and uncomfortable)

: sleep (your baby is sleeping peacefully)

: feeding (your baby is feeding; he or she is sucking and swallowing)

Exemple :

Après-midi	/	/	/	/	P	P	—	—	A	A	P					P	P
	12:00		12:30		13:00		13:30		14:00		14:30		15:00		15:30		16:00

✓ **Record only one behavior in each block**. Sometimes your child may exhibit two different behaviors within a 15-minute period; record only the one that was predominant (the long or most notable).

✓ **Carry the crying log with you** and your baby during the day and place it next to where your baby sleeps at night. This way, you can fill it out as as you go.

2. Répondre aux deux questions de bas de page :

1) Mon bébé présente des crises de pleurs

Sans raison apparente : oui ☐ non ☐ *Cocher la bonne case*

Inconsolables : oui ☐ non ☐ *Cocher la bonne case*

Accompagnées d'autres signes : agitation et/ou visage rouge et/ou poings serrés et/ou membres tendus : oui ☐ non ☐ *Cocher la bonne case*

2) C'était une journée habituelle : oui ☐ non ☐ *Cocher la bonne case*

Ce n'était pas une journée habituelle car : …..<br><br>*Remplir les évènements marquants ou comportement inhabituel de votre bébé.*

3. Remplir un feuillet par jour de ce journal numéroté J1 à J7 pendant 7 jours consécutifs.

Groupement Hospitalier de Territoire Saint-Denis Gonesse Plaine de France

École Pratique des Hautes Études | PSL

CHArt Cognitions Humaine et Artificielle

Day 1 – Date:

Search ID: .../.../.../

Nuit												
00:00 Minuit	00:30	01:00	01:30	02:00	02:30	03:00	03:30	04:00	04:30	05:00	05:30	06:00

Matin												
06:00	06:30	07:00	07:30	08:00	08:30	09:00	09:30	10:00	10:30	11:00	11:30	12:00 Midi

Après-midi												
12:00 Midi	12:30	13:00	13:30	14:00	14:30	15:00	15:30	16:00	16:30	17:00	17:30	18:00

Soirée												
18:00	18:30	19:00	19:30	20:00	20:30	21:00	21:30	22:00	22:30	23:00	23:30	00:00 Minuit

Fill in each box for the day based on your baby's main behavior during the corresponding 15-minute period:

[P] : **crying** (your baby cries with every breath and seems very uncomfortable; it is difficult to soothe)

[A] : **restlessness** (your baby is restless, seems upset, moves around a lot, may make some noises or whimper but isn't crying; your baby is awake and uncomfortable)

[/] : **sleep** (your baby is sleeping peacefully)

[–] : **feeding** (your baby is feeding; he or she is sucking and swallowing)

1) Mon bébé présente des crises de pleurs

Sans raison apparente : oui ☐ non ☐ *Cocher la bonne case*

Inconsolables : oui ☐ non ☐ *Cocher la bonne case*

Accompagnées d'autres signes : agitation et/ou visage rouge et/ou poings serrés et/ou membres tendus : oui ☐ non ☐ *Cocher la bonne case*

2) C'était une journée habituelle : oui ☐ non ☐ *Cocher la bonne case*

Ce n'était pas une journée habituelle car : .....

Remplir les évènements marquants ou comportement inhabituel de votre bébé.

Groupement Hospitalier de Territoire Saint-Denis Gonesse Plaine de France

École Pratique des Hautes Études | PSL

CHArt Cognitions Humaine et Artificielle

Day 2 - Date:

Search ID: /../../

Nuit

00:00 Minuit	00:30	01:00	01:30	02:00	02:30	03:00	03:30	04:00	04:30	05:00	05:30	06:00

Matin

06:00	06:30	07:00	07:30	08:00	08:30	09:00	09:30	10:00	10:30	11:00	11:30	12:00 Midi

Après-midi

12:00 Midi	12:30	13:00	13:30	14:00	14:30	15:00	15:30	16:00	16:30	17:00	17:30	18:00

Soirée

18:00	18:30	19:00	19:30	20:00	20:30	21:00	21:30	22:00	22:30	23:00	23:30	00:00 Minuit

Fill in each box for the day based on your baby's main behavior during the corresponding 15-minute period:

P : **crying** (your baby cries with every breath and seems very uncomfortable; it is difficult to soothe)

A: **restlessness** (your baby is restless, seems upset, moves around a lot, may make some noises or whimper but does not cry; your baby is awake and uncomfortable)

/ : **sleep** (your baby is sleeping peacefully)

⊟ : **feeding** (your baby is being fed; he or she is sucking and swallowing)

1) Mon bébé présente des crises de pleurs

Sans raison apparente : oui ☐ non ☐ *Cocher la bonne case*

Inconsolables : oui ☐ non ☐ *Cocher la bonne case*

Accompagnées d'autres signes : agitation et/ou visage rouge et/ou poings serrés et/ou membres tendus : oui ☐ non ☐ *Cocher la bonne case*

2) C'était une journée habituelle : oui ☐ non ☐ *Cocher la bonne case*

Ce n'était pas une journée habituelle car : .....

Remplir les évènements marquants ou comportement inhabituel de votre bébé.

Day 3 – Date:

Search ID: /../../

Nuit

00:00 (Minuit) 00:30 01:00 01:30 02:00 02:30 03:00 03:30 04:00 04:30 05:00 05:30 06:00

Matin

06:00 06:30 07:00 07:30 08:00 08:30 09:00 09:30 10:00 10:30 11:00 11:30 12:00 (Midi)

Après-midi

12:00 (Midi) 12:30 13:00 13:30 14:00 14:30 15:00 15:30 16:00 16:30 17:00 17:30 18:00

Soirée

18:00 18:30 19:00 19:30 20:00 20:30 21:00 21:30 22:00 22:30 23:00 23:30 00:00 (Minuit)

Fill in each box for the day based on your baby's main behavior during the corresponding 15-minute period:

P : **crying** (your baby cries with every breath and seems very uncomfortable; it is difficult to soothe)

A: **restlessness** (your baby is restless, seems upset, moves around a lot, may make some noises or whimper but does not cry; your baby is awake and uncomfortable)

/ : **sleep** (your baby is sleeping peacefully)

— : **feeding** (your child is being fed; he or she is suckling and swallowing)

1) Mon bébé présente des crises de pleurs

Sans raison apparente : oui ☐ non ☐ *Cocher la bonne case*

Inconsolables : oui ☐ non ☐ *Cocher la bonne case*

Accompagnées d'autres signes : agitation et/ou visage rouge et/ou poings serrés et/ou membres tendus : oui ☐ non ☐ *Cocher la bonne case*

2) C'était une journée habituelle : oui ☐ non ☐ *Cocher la bonne case*

Ce n'était pas une journée habituelle car : .....

Remplir les évènements marquants ou comportement inhabituel de votre bébé.

Day 4 - Date:

Search ID: /../../

Nuit	00:00 Minuit	00:30	01:00	01:30	02:00	02:30	03:00	03:30	04:00	04:30	05:00	05:30	06:00

Matin	06:00	06:30	07:00	07:30	08:00	08:30	09:00	09:30	10:00	10:30	11:00	11:30	12:00 Midi

Après-midi	12:00 Midi	12:30	13:00	13:30	14:00	14:30	15:00	15:30	16:00	16:30	17:00	17:30	18:00

Soirée	18:00	18:30	19:00	19:30	20:00	20:30	21:00	21:30	22:00	22:30	23:00	23:30	00:00 Minuit

Fill in each box for the day based on your baby's main behavior during the corresponding 15-minute period:

- P : **crying** (your baby cries with every breath and seems very uncomfortable; it is difficult to soothe)
- A : **restlessness** (your baby is restless, seems upset, moves around a lot, may make some noises or whimper but isn't crying; your baby is awake and uncomfortable)
- ╱ : **sleep** (your baby is sleeping peacefully)
- ⊟ : **feeding** (your baby is feeding; he or she is sucking and swallowing)

1) Mon bébé présente des crises de pleurs

Sans raison apparente : oui ☐ non ☐ *Cocher la bonne case*

Inconsolables : oui ☐ non ☐ *Cocher la bonne case*

Accompagnées d'autres signes : agitation et/ou visage rouge et/ou poings serrés et/ou membres tendus : oui ☐ non ☐ *Cocher la bonne case*

2) C'était une journée habituelle : oui ☐ non ☐ *Cocher la bonne case*

Ce n'était pas une journée habituelle car : .....

Remplir les évènements marquants ou comportement inhabituel de votre bébé.

Day 5 –
Date:

Search ID: /../../

Nuit

00:00 Minuit	00:30	01:00	01:30	02:00	02:30	03:00	03:30	04:00	04:30	05:00	05:30	06:00

Matin

06:00	06:30	07:00	07:30	08:00	08:30	09:00	09:30	10:00	10:30	11:00	11:30	12:00 Midi

Après-midi

12:00 Midi	12:30	13:00	13:30	14:00	14:30	15:00	15:30	16:00	16:30	17:00	17:30	18:00

Soirée

18:00	18:30	19:00	19:30	20:00	20:30	21:00	21:30	22:00	22:30	23:00	23:30	00:00 Minuit

Fill in each box for the day based on your baby's main behavior during the corresponding 15-minute period:

P : **crying** (your baby cries with every breath and seems very uncomfortable; it's hard to soothe them)

A: **restlessness** (your baby is restless, seems upset, moves around a lot, may make some noises or whimper but does not cry; your baby is awake and uncomfortable)

☐ : **sleep** (your baby is sleeping peacefully)

☐ : **feeding** (your baby is feeding; he or she is sucking and swallowing)

1) Mon bébé présente des crises de pleurs

Sans raison apparente : oui ☐ non ☐ *Cocher la bonne case*

Inconsolables : oui ☐ non ☐ *Cocher la bonne case*

Accompagnées d'autres signes : agitation et/ou visage rouge et/ou poings serrés et/ou membres tendus : oui ☐ non ☐ *Cocher la bonne case*

2) C'était une journée habituelle : oui ☐ non ☐ *Cocher la bonne case*

Ce n'était pas une journée habituelle car : …..

Remplir les évènements marquants ou comportement inhabituel de votre bébé.

Groupement Hospitalier de Territoire Saint-Denis Gonesse Plaine de France

École Pratique des Hautes Études | PSL

CHArt Cognitions Humaine et Artificielle

Day 6 –
Date:

Search ID: /../../

Nuit

00:00 Minuit | 00:30 | 01:00 | 01:30 | 02:00 | 02:30 | 03:00 | 03:30 | 04:00 | 04:30 | 05:00 | 05:30 | 06:00

Matin

06:00 | 06:30 | 07:00 | 07:30 | 08:00 | 08:30 | 09:00 | 09:30 | 10:00 | 10:30 | 11:00 | 11:30 | 12:00 Midi

Après-midi

12:00 Midi | 12:30 | 13:00 | 13:30 | 14:00 | 14:30 | 15:00 | 15:30 | 16:00 | 16:30 | 17:00 | 17:30 | 18:00

Soirée

18:00 | 18:30 | 19:00 | 19:30 | 20:00 | 20:30 | 21:00 | 21:30 | 22:00 | 22:30 | 23:00 | 23:30 | 00:00 Minuit

Fill in each box for the day based on your baby's main behavior during the corresponding 15-minute period:

P : **crying** (your baby cries with every breath and seems very uncomfortable; it's hard to soothe them)

A : **restlessness** (your baby is restless, seems upset, moves around a lot, may make some noises or whimper but isn't crying; your baby is awake and uncomfortable)

☐ : **Sleep** (your baby is sleeping peacefully)

☐ : **feeding** (your baby is feeding; he or she is sucking and swallowing)

1) Mon bébé présente des crises de pleurs

Sans raison apparente : oui ☐ non ☐ *Cocher la bonne case*

Inconsolables : oui ☐ non ☐ *Cocher la bonne case*

Accompagnées d'autres signes : agitation et/ou visage rouge et/ou poings serrés et/ou membres tendus : oui ☐ non ☐ *Cocher la bonne case*

2) C'était une journée habituelle : oui ☐ non ☐ *Cocher la bonne case*

Ce n'était pas une journée habituelle car : .....

Remplir les évènements marquants ou comportement inhabituel de votre bébé.

Day 7 –
Date:

Search ID: /../../

Nuit	00:00 Minuit	00:30	01:00	01:30	02:00	02:30	03:00	03:30	04:00	04:30	05:00	05:30	06:00

Matin	06:00	06:30	07:00	07:30	08:00	08:30	09:00	09:30	10:00	10:30	11:00	11:30	12:00 Midi

Après-midi	12:00 Midi	12:30	13:00	13:30	14:00	14:30	15:00	15:30	16:00	16:30	17:00	17:30	18:00

Soirée	18:00	18:30	19:00	19:30	20:00	20:30	21:00	21:30	22:00	22:30	23:00	23:30	00:00 Minuit

Fill in each box for the day based on your baby's main behavior during the corresponding 15-minute period:

P : **crying** (your baby cries with every breath and seems very uncomfortable; it's hard to calm them down)

A: **restlessness** (your baby is restless, seems upset, moves around a lot, may make some noises or whimper but isn't crying; your baby is awake and uncomfortable)

☐ : **sleep** (your baby is sleeping peacefully)

☐ : **feeding** (your child is being fed; they are suckling and swallowing)

1) Mon bébé présente des crises de pleurs

Sans raison apparente : oui ☐ non ☐ *Cocher la bonne case*

Inconsolables : oui ☐ non ☐ *Cocher la bonne case*

Accompagnées d'autres signes : agitation et/ou visage rouge et/ou poings serrés et/ou membres tendus : oui ☐ non ☐ *Cocher la bonne case*

2) C'était une journée habituelle : oui ☐ non ☐ *Cocher la bonne case*

Ce n'était pas une journée habituelle car : .....

Remplir les évènements marquants ou comportement inhabituel de votre bébé.

APPENDIX 5 – QUESTIONNAIRE A: SOCIO-DEMOGRAPHIC AND MEDICAL DATA

Date:

Search ID: ……./……/…….

1. Administrative Information

- CHILD

Initials:

Date of birth: Gender:

Age: Corrected age:

- MOTHER

Initials:

Occupation (socio-professional category): Address:

Phone:

Email:

- FATHER

Initials:

Occupation (socio-professional category): Phone:

Email:

- **Sibling**

s: Number:

Ages:

2. Obstetric and Medical Data

➢ **Maternal**

History: Previous

Pregnancies:

- Gestational history (number of pregnancies):
- Parity (number of deliveries after 22 weeks of

gestation): Singleton or multiple pregnancy:

Maternal age:

Pre-existing conditions:

Pregnancy-related conditions:

Pain: location:

Duration (in days):

VAS on a scale of 10:

Smoking: yes / no

Treatments received during pregnancy:

- Medications:
- Other: osteopathy/chiropractic; physical

therapy; psychologist Stress level during pregnancy,

on a scale of 1 to 10:

➢ **Pediatric**

Medical conditions:

3. Birth Data

- Gestational age at birth: GW (gestational weeks)
- Type:
- Vaginal delivery: induced / spontaneous
- Cesarean section: elective / emergency
- Complications:
- Instrumentation:
- Duration (in min):
- Work:
- Expulsion:
- APGAR:
- Measurements:
- Weight: g
- Height: cm
- Head circumference: cm
- Treatment received:
- Other:

4. Child's medical history

➢ Medical:

➢ Surgical:

➢ Treatments received:
- Medications: Yes No
- Other: Osteopathy/chiropractic, no. of sessions

5. General condition (according to crying log)

➢ Crying: average hours per day

➢ Feeding:
- bottle-fed/breastfed/mixed
- Possible issues:

➢ Sleep: average hours per day
- Average number of times waking up per night:
- Number of naps:

Notes:

APPENDIX 6: COLIQ® QUESTIONNAIRE

Crying and Discomfort in Infants

GENERAL INFORMATION

Questionnaire Title: ColiQ Questionnaire (Infant Colic Quality of Life Questionnaire)

Version: Validated in 2021

Reference: Archives of Pediatrics 2021

Type of Measure: Patient-Reported Outcome (PRO) - Parent Questionnaire **Target Population:** Parents of infants with excessive crying/colic **Time to Complete:** 10–15 minutes

Administration method: Self-administered parental questionnaire (investigator supervision possible)

Reference period: Last 7 days

QUESTIONNAIRE STRUCTURE

Overview

- **Total: 14 main questions**
- **Module A - Infant Symptoms:** 10 questions (Infant Well-being Score)
- **Module B - Impact on Parents:** 6 questions (Parent Well-being Score)

SECTION 1: PATIENT INFORMATION

Field	Format	Required
Patient ID	[_________]	☑
Infant's Date of Birth	[//____] (DD/MM/YYYY)	☑
Infant's age	[__] weeks	☑
Date filled out	[//____] (DD/MM/YYYY)	☑
Visit	☐ Inclusion ☐ Appointment 1 ☐ Appointment 2	☑
Filled out by	☐ Mother ☐ Father ☐ Both	☑

MODULE A: INFANT SYMPTOMS (Baby Score)

Instructions for parents: *The following questions concern your baby's behavior over the past 7 days. Please answer based on your observations and your impressions.*

DIMENSION 1: QUANTITATIVE DESCRIPTION OF CRYING

Q1. How often does your baby cry?

0 = Never | 10 = Constantly

0 ------- 1 ------- 2 ------- 3 ------- 4 ------- 5 ------- 6 ------- 7 ------- 8 ------- 9 ------- 10

☐ ☐ ☐ ☐ ☐ ☐ ☐ ☐ ☐ ☐ ☐

Score: [___/10]

Q2. When your baby cries, how long does the crying usually last?

0 = Never | 10 = Constantly

0 ------- 1 ------- 2 ------- 3 ------- 4 ------- 5 ------- 6 ------- 7 ------- 8 ------- 9 ------- 10

☐ ☐ ☐ ☐ ☐ ☐ ☐ ☐ ☐ ☐ ☐

Score: [___/10]

Q3. On average, how many hours a day does your baby cry?

☐ Less than 1 hour ☐ 1 to 2 hours ☐ 2 to 3 hours ☐ More than 3 hours

0 1 3 6

Score: [___/10]

DIMENSION 2: QUALITATIVE DESCRIPTION OF CRYING

Q4. How would you describe your baby's crying? *(Multiple answers possible)*

☐ High-pitched/piercing ☐ Screaming ☐ Prolonged ☐ Normal

4 4 3 1

Score: [___/10]

DIMENSION 3: ASSOCIATED SYMPTOMS

Q5. Does your baby exhibit the following symptoms during or after crying?
(Check YES or NO for each item)

Symptom	YES	NO
Hard stomach	☐	☐
Bloated stomach	☐	☐
Legs drawn up to the abdomen	☐	☐
Passing gas	☐	☐
Red face	☐	☐
Tense face	☐	☐
Restlessness/nervousness	☐	☐
Arches back	☐	☐
Squirming	☐	☐
Has clenched fists	☐	☐

1 point / YES

Score: [___/10]

DIMENSION 4: DIGESTIVE PAIN AND DISCOMFORT

Q6. On a scale of 0 to 10, how would you rate your baby's pain/discomfort?

0 = No pain | 10 = Maximum pain

0 ------- 1 ------- 2 ------- 3 ------- 4 ------- 5 ------- 6 ------- 7 ------- 8 ------- 9 ------- 10
☐ ☐ ☐ ☐ ☐ ☐ ☐ ☐ ☐ ☐ ☐

Score: [___/10]

DIMENSION 5: INFANT BEHAVIOR

Q7. When your baby cries, are you unable to comfort him or her?

0 = Never | 10 = All the time

0 ------- 1 ------- 2 ------- 3 ------- 4 ------- 5 ------- 6 ------- 7 ------- 8 ------- 9 ------- 10
☐ ☐ ☐ ☐ ☐ ☐ ☐ ☐ ☐ ☐ ☐

Score: [___/10]

Q8. Is your baby restless when sleeping?

0 = Never | 10 = All the time

0 ------- 1 ------- 2 ------- 3 ------- 4 ------- 5 ------- 6 ------- 7 ------- 8 ------- 9 ------- 10

☐ ☐ ☐ ☐ ☐ ☐ ☐ ☐ ☐ ☐ ☐

Score: [___/10]

Q10. After sleeping, does your baby seem tired?

0 = Never | 10 = All the time

0 ------- 1 ------- 2 ------- 3 ------- 4 ------- 5 ------- 6 ------- 7 ------- 8 ------- 9 ------- 10

☐ ☐ ☐ ☐ ☐ ☐ ☐ ☐ ☐ ☐ ☐

Score: [___/10]

MODULE B: IMPACT ON PARENTS (Parent Score)

Instructions for Parents: *The following questions concern the impact of your baby's crying on your daily life and well-being over the past 7 days.*

DIMENSION 1: ACTIONS AND SOLUTIONS

Q11. What actions have you taken to soothe your baby's crying?
(Multiple answers allowed)

☐ *Holding/rocking* ☐ *Pacifier/sucking* ☐ *Changing position* ☐ *Tummy rub* ☐ *Bath* ☐ *Walk/outing* ☐ *Music/white noise* ☐ *Change in diet* ☐ *Medical consultation* ☐ *Medications/treatments* ☐ *Other:* ______________________________

(Not rated)

DIMENSION 2: PSYCHOLOGICAL IMPACT

Q12. Does your baby's crying cause you to experience the following feelings? *(Scale: 0 = Never | 10 = All the time)*

Feeling	Score 0–10
You find the situation difficult	[__/10]
You feel stressed	[/10]
Do you feel desperate?	[/10]
Do you feel guilty?	[_ /10]

Do you feel useless [__/10]
You're crying [__/10]
You feel depressed [__/10]
You feel helpless [__/10]
You lose your temper and can no longer control yourself [__/10]
You're handling the situation poorly [

_________/10] Score: [_________/100]

DIMENSION 3: IMPACT ON YOUR RELATIONSHIP

Q13. Does your baby's crying have an impact on your relationship? *(If applicable)*

Impact	YES	NO	N/A
Irritation	☐	☐	☐
Impatience	☐	☐	☐
Tension	☐	☐	☐
Arguments	☐	☐	☐
Emotional distance	☐	☐	☐

10 points per "yes" answer

Score: [___/50]

DIMENSION 4: IMPACT ON GENERAL WELL-BEING AND DAILY LIFE

Q14. Because of your baby's crying: ?

Impact	YES	NO
You have trouble sleeping	☐	☐
Are you tired?	☐	☐
You don't get enough sleep	☐	☐
You have pain (back, arm, etc.)	☐	☐

You don't have enough time to:

- yourself ☐ ☐
- take care of household chores ☐ ☐
- your family or your friends ☐ ☐
- going out and seeing people ☐ ☐

10 points/yes

Score: [___/80]

DIMENSION 6: OVERALL IMPACT

Q16. On a scale of 0 to 10, how would you rate the situation you find yourself in today because of your baby's crying?

0 = Easy to handle | 10 = Impossible to handle

0 ------- 1 ------- 2 ------- 3 ------- 4 ------- 5 ------- 6 ------- 7 ------- 8 ------- 9 ------- 10

☐ ☐ ☐ ☐ ☐ ☐ ☐ ☐ ☐ ☐ ☐

Score: [___/10]

SECTION 2: SCORE CALCULATION (To be completed by the investigator)

Calculated Scores

Infant Well-Being Score: - Formula: 100 - (average of symptom severity scores) - Score obtained: [_________________] / 100

Parent Well-Being Score: - Formula: 100 - (average of impact severity scores) - Score obtained: [______________] / 100

Overall ColiQ Score: - Formula: average (Baby Score + Parent Score) - Score obtained: [____] / 100

Range: 0–100 (higher = better condition)

SECTION 3: INVESTIGATOR'S OBSERVATIONS

Comments on the completion of the questionnaire:

____

____

____

Difficulties encountered:

☐ None ☐ Understanding the questions ☐ Emotional burden ☐ Time required to complete ☐ Other: _______________

Questionnaire completed:

☐ Completely ☐ Partially (specify missing questions): __________________________

SECTION 4: VALIDATION

Completed by: ________________________ Date: [//_____]

Verified by (Investigator): ____________________________ Date: [//_____]

Investigator's signature: ______________________________

APPENDIX:

Scientific Rationale

Questionnaire Validation: -

French validation study published in 2021 in Archives de Pédiatrie, based on a validation sample of 1,298 parents of infants –

Demonstrated psychometric properties: - **Test-retest reliability:** ICC > 0.70 - **Internal consistency:** Cronbach's alpha > 0.70 - **Construct validity:** Confirmed –

Sensitivity: Detection of significant changes as early as 1 month ($p < 0.05$)

Ethical Considerations

Benefits: - Standardized and objective assessment of symptom severity - Longitudinal monitoring of progress - Facilitates communication between parents and professionals - Aids in treatment decision-making

Risks: - No physical risks (non-invasive questionnaire) - Potential emotional burden (discussion of difficulties) - Acceptable completion time (10–15 minutes)

Protective Measures: - Support from the investigator if necessary - Option to pause while completing the questionnaire - Psychological support will be recommended if parents experience distress

Use of Data

Primary variables: - Infant Well-being Score (0–100) - Parent Well-being Score (0–100) - Overall ColiQ Score (0–100) - Severity classification (Mild/Moderate/Severe)

Secondary variables: - Scores by dimension - Individual items - Longitudinal changes

Interpretation of Results:

Mild Severity Group: ColiQ score > 70

- Average infant score: 71.57
- Average Parent Score: 74.9

Moderate Severity Group: Colic score between 50 and 70

- Average Baby Score: 58.7
- Average Parent Score: 67.63

High Severity Group: ColiQ score < 50

- Average Baby Score: 39.89
- Average Parent Score: 53.13

APPENDIX 7: INSTRUCTIONS FOR PARENTS ON FILMING GENERAL MOVEMENTS

How to Film Your Baby's General Movements

You will be asked to film your baby's general movements for the research team **15, 30, and 45 days after the first appointment.** A reminder via text message will be sent to you a few days beforehand. The videos will be transferred to a USB drive, which the team will give you at Appointment 1 and collect at Appointment 2.

General movements are your baby's spontaneous movements that occur when they are in a calm, awake state, free to move, without any interaction, pacifier, or toys.

Analyzing these movements is very helpful in assessing your child's development.

To ensure this analysis is possible, there are certain rules to follow:

What to do:

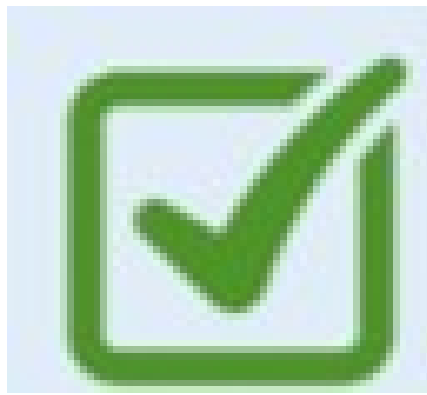

1. Film the child when they are **awake and calm**
2. Place your baby on a **flat, firm surface**: a playmat, blanket, or sheet
3. Your baby is **lying on their back**
4. Your baby is wearing **only a diaper or a onesie**
5. **Film while standing** above your baby
6. The baby's **feet and hands** must be **visible**
7. Keep the **camera steady**
8. Film for **3 minutes**

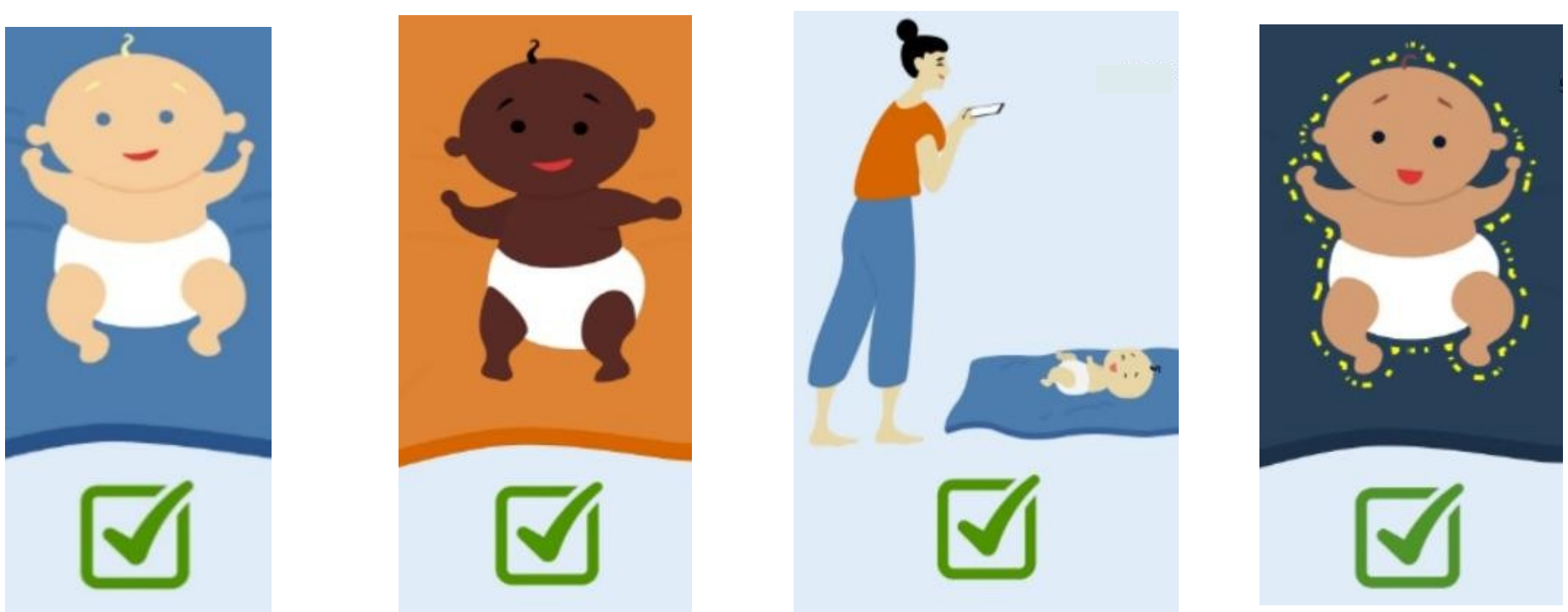

Avoid:

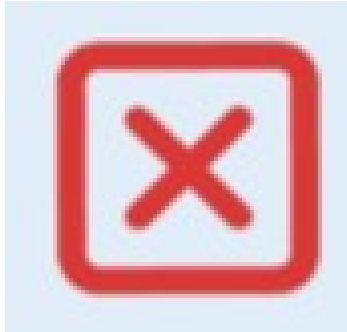

1. Your baby is lying on **their side or stomach**
2. Your baby is lying on **a soft surface** (sofa or bed)
3. Your baby **has access to toys** or a comfort blanket
4. Your baby **has a pacifier**
5. Your baby **is crying or fussing**
6. **Distract or talk** to your baby

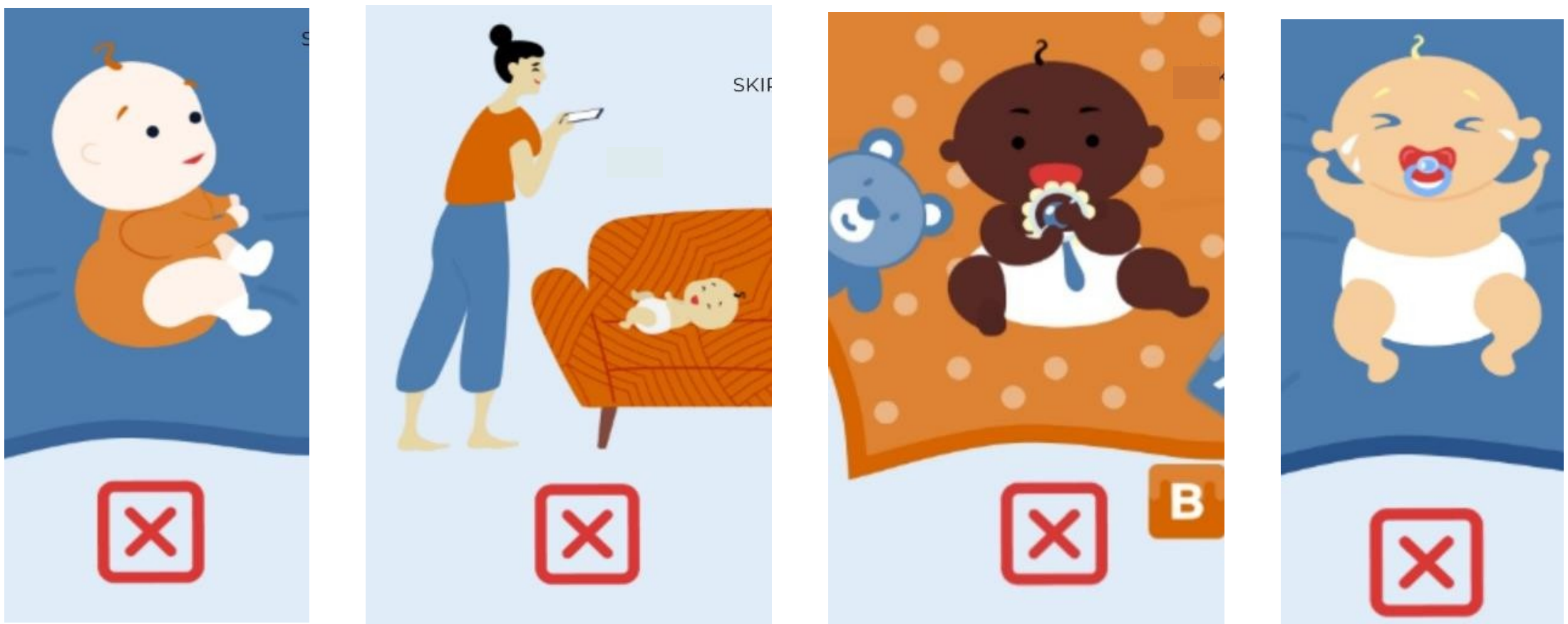

Illustrations and instructions translated from English, Baby Moves® app

APPENDIX 8 – INFRARED THERMAL IMAGING (ITI)

Physiological Interpretation of Thermal Gradients (ΔT)

Thermographic analysis is based on the principle that **skin temperature reflects microvascular perfusion**, which is regulated by the **autonomic nervous system**. Thermal gradients between body regions therefore reflect **changes in sympathetic tone** and **local adjustments in blood flow** related to pain, stress, or thermoregulation.

1. Bilateral ΔT (right/left asymmetries)

A bilateral thermal asymmetry greater than **0.5 °C** is considered abnormal. It suggests **lateralized vasomotor dysregulation**, potentially linked to a **localized nociceptive response** or **asymmetric activation of the sympathetic nervous system**. These thresholds are derived from normative studies of healthy adults (Marins et al., 2014; Ring & Ammer, 2012) and have been confirmed in pathological or experimental contexts in infants.

2. Central–Peripheral ΔT

The **central–peripheral gradient (ΔTcp)** corresponds to the difference between the central regions (thorax, abdomen) and distal (hands, feet).
It reflects the **redistribution of blood flow** between deep and superficial tissues.

A **low ΔTcp (< 0.5 °C)** indicates **stable homeothermy** and good peripheral perfusion.

A **high ΔTcp (> 0.5 °C)** indicates **reflex peripheral vasoconstriction**, observed during **pain**, or **stress** or **hemodynamic** . These phenomena have been described in adults (Marins et al., 2014; Ioannou et al., 2014) and in preterm infants (Hammarlund et al., 1985), where ΔTcp is a sensitive marker of hypoperfusion.

3. Intrafacial ΔT

The **intrafacial gradient (ΔT)** is calculated between emotionally reactive areas (nose, periocular region, chin) and the thermally stable frontal region. A **decrease in temperature** in the nose or periocular region (> 0.5 °C) indicates **sympathetic activation** causing **cutaneous vasoconstriction**, associated with **pain** or to **emotional** . This physiological signature is well documented in infants and children *(Kolosovas-Machuca et al., 2011; Ioannou et al., 2013–2020).*

4. Clinical Significance and Integration

The combination of **bilateral**, **central–peripheral,** and **intrafacial** ΔT values allows for a **multidimensional** assessment **of prolonged pain**:

- **ΔTcp** reflects the **systemic response** (stress, pain)
- **bilateral ΔT** values identify **areas of localized pain**;
- **intrafacial ΔT** values provide information on **emotional and autonomic reactivity**

These parameters, integrated with the **EDIN** behavioral scale and **capillary cortisol**, provide a **multimodal and noninvasive** approach to chronic pain in infants.

Details of regions of interest in whole-body thermal images

ROI No.	Anatomical region	View	Side	ΔT physiological	Notes	References
1–2	**Upper chest**	Anterior	L / R	< 0.4 °C	Stable central zone; strong cutaneous perfusion. Bilateral differences > 0.5 °C may indicate an autonomic .	Marins et al., 2014 ; Ring & Ammer, 2012
3–4	**Upper abdomen**	Anterior	L / R	< 0.5 °C	Metabolically active area, sensitive to digestion and visceral stress. Homogeneous temperature expected.	Lahiri et al., 2012; Niu et al., 2019
5–6	**Lower abdomen**	Anterior	L / R	< 0.5 °C	Visceral reflex zone. Thermal asymmetries may indicate hypertonicity or abdominal discomfort.	Ammer, 2008; Chudecka & Lubkowska, 2012
7–8	**Proximal arm**	Anterior	L / R	< 0.4 °C	Expected thermal stability.	Marins et al., 2014
9–10	**Forearm**	Anterior	L / R	< 0.5 °C	Slight variability possible, homogeneous area	Hildebrandt et al., 2010
11–12	**Hand (palm)**	Anterior	L / R	< 0.5 °C	Distal extremity: area reactive to stress and pain (sympathetic vasoconstriction).	Kolosovas-Machuca et al., 2011; Ioannou et et al., 2013
13–14	**Dorsal region (posterior thorax)**	Posterior	L / R	< 0.5 °C	Possible correlation with postural tone and spinal discomfort.	Ring & Ammer, 2012
15–16	**Lumbar region**	Posterior	D / G	< 0.5 °C	Area Sensitive tension (lumbar (psoas, visceromuscular structures).	Chudecka & Lubkowska, 2012
17–18	**Proximal arm**	Posterior	L / R	< 0.4 °C	Mild bilateral asymmetry expected. Good inter-session reproducibility. Homogeneous area	Marins et al., 2014
19–20	**Forearm**	Posterior	L / R	< 0.5 °C	ΔT values greater than 0.6 °C reflect a physiological response to stress.	Hildebrandt et al., 2010
21–22	**Hand (back)**	Posterior	L / R	< 0.5 °C	High sensitivity to stress, useful for monitoring peripheral vasoconstriction .	Ioannou et et al., 2014
23–24	**Thigh**	Anterior	L / R	< 0.4 °C	Large muscle area; thermal homogeneity expected.	Chudecka et al., 2015
25–26	**Leg**	Anterior	L / R	< 0.5 °C	Homogeneous zone.	Marins et al., 2014
27–28	**Dorsal (anter foot) ior**	Anterior	L / R	< 0.6 °C	Area sensitive to thermoregulation and stress responses.	Kolosovas-Machuca et al., 2011

29–30	**Thigh**	Posterior	D / G	< 0.4 °C	Posterior muscle group ; Stable except for asymmetric posture.	Chudecka & Lubkowska, 2012
31–32	**Leg**	Posterior	D / G	< 0.5 °C	Correlation with perfusion distal. Prolonged asymmetries reflect a sympathetic .	Marins et al., 2014
33–34	**Foot (posterior plantar)**	Posterior	D / G	< 0.6 °C	Posterior posterior end, very stress-responsive. Gradient of temperature gradient associated with low back pain.	Kolosovas-Machuca et al., 2011
35–36	**Occiput**	Posterior	D / G	< 0.4 °C	Thermal thermally stable, used as control in reference.	Ring & Ammer, 2012; Ammer, 2008

Details of Areas of Interest in Facial Thermal Images

ROIv Code	Anatomical Region	Side	Physiological Notes	Main References
**ROIv1**	**Forehead Central (area of Reference)**	Median	The thermally most stable zone, used as a reference for calculating intrafacial ΔT values.	Kolosovas-Machuca & González, 2011; Ioannou et al., 2014; Zhou et al., 2020
**ROIv2**	**Nose**	Median	Area with a high density of arteriovenous anastomoses, highly sensitive to sympathetic vasoconstriction; rapid temperature changes during stress or pain.	Ioannou et al., 2014, 2017; Kolosovas-Machuca, 2011; Merla & Romani, 2010; Marins et al., 2014
**ROIv3**	**Periocular region**	Bilateral	Rich in microcirculation, sensitive to sympathetic activation; correlated with limbic system activity; reliable for stress detection.	Ioannou et al., 2013–2020; Zhou, 2020; Honner & Kosová, 2022
**ROIv4**	**Perioral region**	Median	Responds to emotions and oral motor activity (crying, sucking); moderate thermal variations possible during stress.	Ioannou et al., 2017; Kolosovas-Machuca & González, *Skin Res. Technol.*, 2011
**ROIv5**	**Chin region**	Median	Peripheral zone; useful for detecting lower thermal asymmetries; more influenced by posture.	Kolosovas-Machuca & González, 2011; Ioannou et al., 2014; Marins et al., 2014

Table of Data to Be Collected: Body ROI

ROI No.	Anatomical region	Plane	Side	Average temperature (°C)	Bilateral ΔT (°C)	ΔT central–peripheral (°C)
1	**Upper chest**	Anterior	Right	T_ROI01	=ABS(T_ROI01 - - T_ROI02)	=T_ROI01 - T_ROI11
2	**Upper chest**	Anterior	Left	T_ROI02	same as	=T_ROI02 - T_ROI12
3	**Upper abdomen**	Anterior	Right	T_ROI03	=ABS(T_ROI03 - T_ROI04)	=T_ROI03 - T_ROI27
4	**Upper abdomen**	Anterior	Left	T_ROI04	same as	=T_ROI04 - T_ROI28
5	**Lower abdomen**	Anterior	Right	T_ROI05	=ABS(T_ROI05 - T_ROI06)	=T_ROI05 - T_ROI27
6	**Lower abdomen**	Anterior	Left	T_ROI06	same	=T_ROI06 - T_ROI28
7	**Proximal arm**	Anterior	Right	T_ROI07	=ABS(T_ROI07 - - T_ROI08)	
8	**Proximal arm**	Anterior	Left	T_ROI08	same	
9	**Forearm**	Anterior	Straight	T_ROI09	=ABS(T_ROI09 - T_ROI10)	
10	**Forearm**	Anterior	Left	T_ROI10	same	
11	**Hand (palm)**	Anterior	Right	T_ROI11	=ABS(T_ROI11 - - T_ROI12)	—
12	**Hand (palm)**	Anterior	Left	T_ROI12	same	—
13	**Dorsal region**	Posterior	Right	T_ROI13	=ABS(T_ROI13 - T_ROI14)	=T_ROI13 - T_ROI21
14	**Dorsal region**	Posterior	Left	T_ROI14	same	=T_ROI14 - T_ROI22
15	**Lumbar region**	Posterior	Right	T_ROI15	=ABS(T_ROI15 - T_ROI16)	=T_ROI15 - T_ROI33
16	**Lumbar region**	Posterior	Left	T_ROI16	same	=T_ROI16 - T_ROI34
17	**Proximal arm**	Posterior	Right	T_ROI17	=ABS(T_ROI17 - T_ROI18)	
18	**Proximal arm**	Posterior	Left	T_ROI18	same	
19	**Forearm**	Posterior	Straight	T_ROI19	=ABS(T_ROI19 - T_ROI20)	
20	**Forearm**	Posterior	Left	T_ROI20	same	
21	**Hand (back)**	Back	Right	T_ROI21	=ABS(T_ROI21 - T_ROI22)	—
22	**Hand (back)**	Back	Left	T_ROI22	same	—
23	**Thigh**	Anterior	Straight	T_ROI23	=ABS(T_ROI23 - T_ROI24)	
24	**Thigh**	Front	Left	T_ROI24	same	
25	**Leg**	Anterior	Right	T_ROI25	=ABS(T_ROI25 - T_ROI26)	
26	**Leg**	Front	Left	T_ROI26	same	

**27**	**Foot (dorsal surface)**	Anterior	Right	T_ROI27	=ABS(T_ROI27 - T_ROI28)	—
**28**	**Foot (dorsal surface)**	Anterior	Left	T_ROI28	same	—
**29**	**Thigh**	Back	Straight	T_ROI29	=ABS(T_ROI29 - T_ROI30)	
**30**	**Thigh**	Back	Left	T_ROI30	same	
**31**	**Leg**	Posterior	Right	T_ROI31	=ABS(T_ROI31 - T_ROI32)	
**32**	**Leg**	Back	Left	T_ROI32	same	
**33**	**Foot (plantar surface)**	Posterior	Right	T_ROI33	=ABS(T_ROI33 - T_ROI34)	—
**34**	**Foot (plantar surface)**	Posterior	Left	T_ROI34	same	—
**35**	**Occiput**	Posterior	Straight	T_ROI35	=ABS(T_ROI35 - T_ROI36)	
**36**	**Occiput**	Posterior	Left	T_ROI36	same	

Principles and Formulas Used

Bilateral ΔT = ABS(T_D - T_G)

Difference between the average temperatures of the right and left ROIs within the same region.
→ Anomaly threshold: > 0.5 °C *(Marins et al., 2014; Kolosovas et al., 2011)*

Central–peripheral ΔT = T_central - T_peripheral

Difference between the average proximal and distal temperatures on the same side: right upper thoracic region and right right for example.
→ Abnormality threshold: > 0.8 °C anterior, > 1 °C posterior *(Marins et al. 2014)*

Average ROI T: average of the pixels in the region of interest defined according to the plane.

Table of data to be collected for the facial ROI

ROI code	Anatomical region	Side	Average temperature (°C)	ΔT Intrafacial (°C)	Notes / Excel Formula
**RIOv1**	Central front *(reference area)*	Median	T_RIOv1	—	Thermal reference zone (stable, used for ΔT calculations)
**RIOv2**	Nose (nasal dorsum)	Median	T_RIOv2	=T_RIOv2 – - T_RIOv1	ΔT nose–forehead: marker of stress/pain (Ioannou, Kolosovas)
**RIOv3**	Periocular region	Right	T_RIOv3	=T_RIOv3 - T_RIOv1	ΔT periocular D – forehead
**RIOv4**	Periocular region	Left	T_RIOv4	=T_RIOv4 - T_RIOv1	ΔT periocular G – forehead
**RIOv5**	Perioral region	Median	T_RIOv5	=T_RIOv5 - T_RIOv1	ΔT perioral – forehead
**RIOv6**	Chin region	Median	T_RIOv6	=T_RIOv6 - T_RIOv1	ΔT chin – forehead

Principles and formula used:

ΔT intrafacial = T_zone - T_forehead (with RIOv1 as the central reference)

A difference > 0.5 °C between a peripheral area (nose, periocular region, chin) and the forehead indicates sympathetic activation related to stress or pain (*Ioannou et al., 2013–2020; Kolosovas et al., 2011).*

The central forehead (RIOv1) is used as a reference because it is thermally more stable and less influenced by local sympathetic microcirculation.
