# Supplementary material for: Beyond crying: Protocol for a multimodal assessment of suspected prolonged pain in infants using infrared thermal imaging, behavioral assessment and physiological biomarkers (NEOTHERM)
Source: PLoS One. 2026 Nov 12;21(11):e0361344. doi: 10.1371/journal.pone.0361344 (PMC13759114; doi:10.1371/journal.pone.0361344)
Supplement: Supplementary file 2 [file pone.0361344.s002.pdf]

NEOTHERM : 7-days Crying diary

Instructions

This is a log of your baby's behavior over 7 consecutive days.

Each day is represented by **4 horizontal bars** (night/morning/afternoon, and evening), divided into boxes according to the following layout:

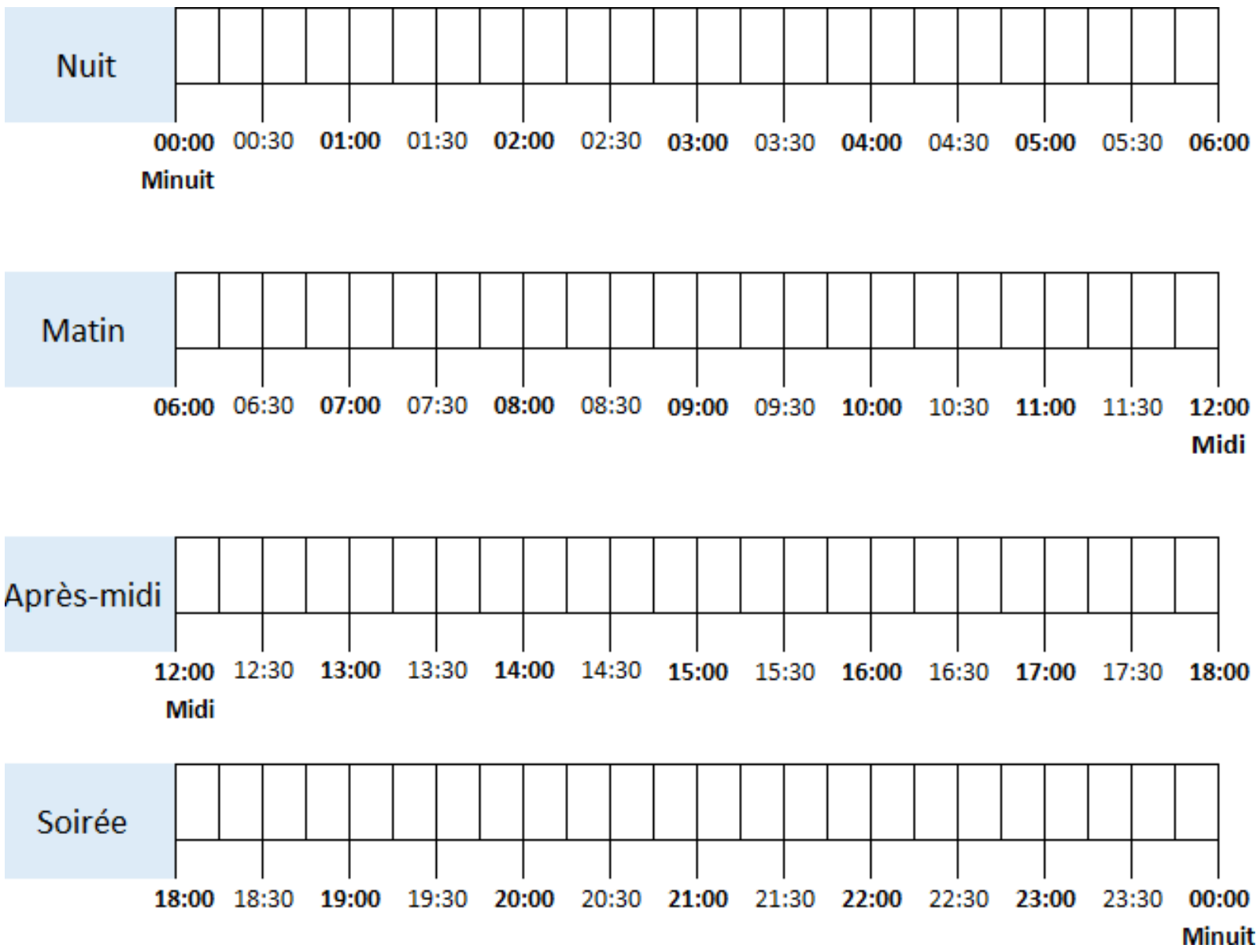

Each **box** represents **15 minutes** (4 boxes per hour), and **the time is indicated below the horizontal bar.**

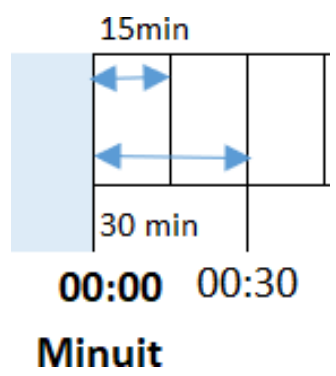

How to fill out this log:

1. **Fill in each box for the day based on your baby's main behavior during the corresponding 15-minute period:**

P : **crying** (your baby cries with every breath and seems very uncomfortable; it's hard to soothe)

A : **restlessness** (your baby is restless, seems upset, moves around a lot, may make some noises or whimper but isn't crying; your baby is awake and uncomfortable)

╱ : **sleep** (your baby is sleeping peacefully)

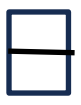 **: feeding** (your baby is feeding; he or she is sucking and swallowing)

Groupement Hospitalier de Territoire Saint-Denis Gonesse Plaine de France

École Pratique des Hautes Études | PSL

CHArt Cognitions Humaine et Artificielle

Exemple :

Après-midi					P	P			A	A	P					P	P
	**12:00**		12:30		**13:00**		13:30		**14:00**		14:30		**15:00**		15:30		**16:00**

- ✓ **Record only one behavior in each block**. Sometimes your child may exhibit two different behaviors within a 15-minute period; record only the one that was the most prominent (the longest-lasting or most notable).
- ✓ **Carry the crying log with you** and your baby during the day, and place it next to where your baby sleeps at night. This way, you can fill it out as events occur.

2. Answer the two questions at the bottom of the page

1) <u>My baby has crying episodes</u>

Without an apparent reason: yes ☐ no ☐ Check the appropriate box

Inconsolable: yes ☐ no ☐ Check the appropriate box

Accompanied by other signs: agitation and/or flushed face and/or clenched fists and/or extended legs: yes ☐ no ☐ Check the appropriate box

2) It was a typical day: yes ☐ no ☐ Check the appropriate box

It was not a typical day because: ……

Describe any significant events or unusual behavior observed in your baby.

3. Complete one page per day (J1 to J7) for seven consecutive days.

Groupement Hospitalier de Territoire Saint-Denis Gonesse Plaine de France

École Pratique des Hautes Études | PSL

CHArt Cognitions Humaine et Artificielle

Day 1 – Date:

Research ID: .../.../.../

Nuit	**00:00** **Minuit**	00:30	**01:00**	01:30	**02:00**	02:30	**03:00**	03:30	**04:00**	04:30	**05:00**	05:30	**06:00**

Matin	**06:00**	06:30	**07:00**	07:30	**08:00**	08:30	**09:00**	09:30	**10:00**	10:30	**11:00**	11:30	**12:00** **Midi**

Après-midi	**12:00** **Midi**	12:30	**13:00**	13:30	**14:00**	14:30	**15:00**	15:30	**16:00**	16:30	**17:00**	17:30	**18:00**

Soirée	**18:00**	18:30	**19:00**	19:30	**20:00**	20:30	**21:00**	21:30	**22:00**	22:30	**23:00**	23:30	**00:00** **Minuit**

Fill in each box for the day based on your baby's main behavior during the corresponding 15-minute period:

P : **crying** (your baby cries with every breath and seems very uncomfortable; it's hard to soothe him or her)

A : **restlessness** (your baby is restless, seems upset, moves around a lot, may make some noises or whimper but isn't crying; your baby is awake and uncomfortable)

⧄ : **sleep** (your baby is sleeping peacefully)

⊟ : **feeding** (your baby is being fed; he or she is sucking and swallowing)

1) My baby has crying episodes

Without an apparent reason:	yes ☐	no ☐	Check the appropriate box
Inconsolable:	yes ☐	no ☐	Check the appropriate box
Accompanied by other signs: agitation and/or flushed face and/or clenched fists and/or extended legs:	yes ☐	no ☐	Check the appropriate box
**2) It was a typical day:**	yes ☐	no ☐	Check the appropriate box

It was not a typical day because: ......

Describe any significant events or unusual behavior observed in your baby.

Groupement Hospitalier de Territoire Saint-Denis Gonesse Plaine de France

École Pratique des Hautes Études | PSL

CHArt Cognitions Humaine et Artificielle

Day 2 – Date:

Research ID: .../.../.../

Nuit

00:00 Minuit | 00:30 | 01:00 | 01:30 | 02:00 | 02:30 | 03:00 | 03:30 | 04:00 | 04:30 | 05:00 | 05:30 | 06:00

Matin

06:00 | 06:30 | 07:00 | 07:30 | 08:00 | 08:30 | 09:00 | 09:30 | 10:00 | 10:30 | 11:00 | 11:30 | 12:00 Midi

Après-midi

12:00 Midi | 12:30 | 13:00 | 13:30 | 14:00 | 14:30 | 15:00 | 15:30 | 16:00 | 16:30 | 17:00 | 17:30 | 18:00

Soirée

18:00 | 18:30 | 19:00 | 19:30 | 20:00 | 20:30 | 21:00 | 21:30 | 22:00 | 22:30 | 23:00 | 23:30 | 00:00 Minuit

Fill in each box for the day based on your baby's main behavior during the corresponding 15-minute period:

P : **crying** (your baby cries with every breath and seems very uncomfortable; it's hard to soothe him or her)

A : **restlessness** (your baby is restless, seems upset, moves around a lot, may make some noises or whimper but isn't crying; your baby is awake and uncomfortable)

/ : **sleep** (your baby is sleeping peacefully)

⊟ : **feeding** (your baby is being fed; he or she is sucking and swallowing)

1) My baby has crying episodes

Without an apparent reason:	yes ☐	no ☐	Check the appropriate box
Inconsolable:	yes ☐	no ☐	Check the appropriate box
Accompanied by other signs: agitation and/or flushed face and/or clenched fists and/or extended legs:	yes ☐	no ☐	Check the appropriate box
**2) It was a typical day:**	yes ☐	no ☐	Check the appropriate box

It was not a typical day because: ......

Describe any significant events or unusual behavior observed in your baby.

Groupement Hospitalier de Territoire
Saint-Denis Gonesse
Plaine de France

École Pratique des Hautes Études | PSL

CHArt Cognitions Humaine et Artificielle

Day 3 – Date:

Research ID: .../.../.../

Nuit	00:00 Minuit	00:30	01:00	01:30	02:00	02:30	03:00	03:30	04:00	04:30	05:00	05:30	06:00

Matin	06:00	06:30	07:00	07:30	08:00	08:30	09:00	09:30	10:00	10:30	11:00	11:30	12:00 Midi

Après-midi	12:00 Midi	12:30	13:00	13:30	14:00	14:30	15:00	15:30	16:00	16:30	17:00	17:30	18:00

Soirée	18:00	18:30	19:00	19:30	20:00	20:30	21:00	21:30	22:00	22:30	23:00	23:30	00:00 Minuit

Fill in each box for the day based on your baby's main behavior during the corresponding 15-minute period:

P **: crying** (your baby cries with every breath and seems very uncomfortable; it's hard to soothe him or her)

A **: restlessness** (your baby is restless, seems upset, moves around a lot, may make some noises or whimper but isn't crying; your baby is awake and uncomfortable)

/ **: sleep** (your baby is sleeping peacefully)

⊟ **: feeding** (your baby is being fed; he or she is sucking and swallowing)

1) My baby has crying episodes

Without an apparent reason:	yes ☐	no ☐	Check the appropriate box
Inconsolable:	yes ☐	no ☐	Check the appropriate box
Accompanied by other signs: agitation and/or flushed face and/or clenched fists and/or extended legs:	yes ☐	no ☐	Check the appropriate box
**2) It was a typical day:**	yes ☐	no ☐	Check the appropriate box

It was not a typical day because: ......

Describe any significant events or unusual behavior observed in your baby.

Groupement Hospitalier de Territoire Saint-Denis Gonesse Plaine de France — École Pratique des Hautes Études | PSL — CHArt Cognitions Humaine et Artificielle

Day 4 – Date:

Research ID: .../.../.../

Nuit

00:00 Minuit | 00:30 | 01:00 | 01:30 | 02:00 | 02:30 | 03:00 | 03:30 | 04:00 | 04:30 | 05:00 | 05:30 | 06:00

Matin

06:00 | 06:30 | 07:00 | 07:30 | 08:00 | 08:30 | 09:00 | 09:30 | 10:00 | 10:30 | 11:00 | 11:30 | 12:00 Midi

Après-midi

12:00 Midi | 12:30 | 13:00 | 13:30 | 14:00 | 14:30 | 15:00 | 15:30 | 16:00 | 16:30 | 17:00 | 17:30 | 18:00

Soirée

18:00 | 18:30 | 19:00 | 19:30 | 20:00 | 20:30 | 21:00 | 21:30 | 22:00 | 22:30 | 23:00 | 23:30 | 00:00 Minuit

Fill in each box for the day based on your baby's main behavior during the corresponding 15-minute period:

P : **crying** (your baby cries with every breath and seems very uncomfortable; it's hard to soothe him or her)

A : **restlessness** (your baby is restless, seems upset, moves around a lot, may make some noises or whimper but isn't crying; your baby is awake and uncomfortable)

╱ : **sleep** (your baby is sleeping peacefully)

⊟ : **feeding** (your baby is being fed; he or she is sucking and swallowing)

1) My baby has crying episodes

Without an apparent reason: yes ☐ no ☐ Check the appropriate box

Inconsolable: yes ☐ no ☐ Check the appropriate box

Accompanied by other signs: agitation and/or flushed face and/or clenched fists and/or extended legs: yes ☐ no ☐ Check the appropriate box

2) It was a typical day: yes ☐ no ☐ Check the appropriate box

It was not a typical day because: ......

Describe any significant events or unusual behavior observed in your baby.

Groupement Hospitalier de Territoire Saint-Denis Gonesse Plaine de France

École Pratique des Hautes Études | PSL

CHArt Cognitions Humaine et Artificielle

Day 5 – Date:

Research ID: .../.../.../

Nuit

00:00 Minuit	00:30	01:00	01:30	02:00	02:30	03:00	03:30	04:00	04:30	05:00	05:30	06:00

Matin

06:00	06:30	07:00	07:30	08:00	08:30	09:00	09:30	10:00	10:30	11:00	11:30	12:00 Midi

Après-midi

12:00 Midi	12:30	13:00	13:30	14:00	14:30	15:00	15:30	16:00	16:30	17:00	17:30	18:00

Soirée

18:00	18:30	19:00	19:30	20:00	20:30	21:00	21:30	22:00	22:30	23:00	23:30	00:00 Minuit

Fill in each box for the day based on your baby's main behavior during the corresponding 15-minute period:

[P] : **crying** (your baby cries with every breath and seems very uncomfortable; it's hard to soothe him or her)

[A] : **restlessness** (your baby is restless, seems upset, moves around a lot, may make some noises or whimper but isn't crying; your baby is awake and uncomfortable)

[/] : **sleep** (your baby is sleeping peacefully)

[⊟] : **feeding** (your baby is being fed; he or she is sucking and swallowing)

1) <u>My baby has crying episodes</u>

Without an apparent reason:	yes ☐	no ☐	Check the appropriate box
Inconsolable:	yes ☐	no ☐	Check the appropriate box
Accompanied by other signs: agitation and/or flushed face and/or clenched fists and/or extended legs:	yes ☐	no ☐	Check the appropriate box
**2) It was a typical day:**	yes ☐	no ☐	Check the appropriate box

It was not a typical day because: ......

Describe any significant events or unusual behavior observed in your baby.

Day 6 – Date:

Research ID: …/…/…/

Nuit

00:00 Minuit	00:30	01:00	01:30	02:00	02:30	03:00	03:30	04:00	04:30	05:00	05:30	06:00

Matin

06:00	06:30	07:00	07:30	08:00	08:30	09:00	09:30	10:00	10:30	11:00	11:30	12:00 Midi

Après-midi

12:00 Midi	12:30	13:00	13:30	14:00	14:30	15:00	15:30	16:00	16:30	17:00	17:30	18:00

Soirée

18:00	18:30	19:00	19:30	20:00	20:30	21:00	21:30	22:00	22:30	23:00	23:30	00:00 Minuit

Fill in each box for the day based on your baby's main behavior during the corresponding 15-minute period:

P : **crying** (your baby cries with every breath and seems very uncomfortable; it's hard to soothe him or her)

A : **restlessness** (your baby is restless, seems upset, moves around a lot, may make some noises or whimper but isn't crying; your baby is awake and uncomfortable)

/ : **sleep** (your baby is sleeping peacefully)

— : **feeding** (your baby is being fed; he or she is sucking and swallowing)

1) My baby has crying episodes

Without an apparent reason:	yes ☐	no ☐	Check the appropriate box
Inconsolable:	yes ☐	no ☐	Check the appropriate box
Accompanied by other signs: agitation and/or flushed face and/or clenched fists and/or extended legs:	yes ☐	no ☐	Check the appropriate box
**2) It was a typical day:**	yes ☐	no ☐	Check the appropriate box

It was not a typical day because: ……

Describe any significant events or unusual behavior observed in your baby.

Groupement Hospitalier de Territoire Saint-Denis Gonesse Plaine de France

École Pratique des Hautes Études | PSL

CHArt Cognitions Humaine et Artificielle

Day 7 – Date:

Research ID: .../.../.../

	00:00 Minuit	00:30	01:00	01:30	02:00	02:30	03:00	03:30	04:00	04:30	05:00	05:30	06:00
Nuit													

	06:00	06:30	07:00	07:30	08:00	08:30	09:00	09:30	10:00	10:30	11:00	11:30	12:00 Midi
Matin													

	12:00 Midi	12:30	13:00	13:30	14:00	14:30	15:00	15:30	16:00	16:30	17:00	17:30	18:00
Après-midi													

	18:00	18:30	19:00	19:30	20:00	20:30	21:00	21:30	22:00	22:30	23:00	23:30	00:00 Minuit
Soirée													

Fill in each box for the day based on your baby's main behavior during the corresponding 15-minute period:

P : **crying** (your baby cries with every breath and seems very uncomfortable; it's hard to soothe him or her)

A : **restlessness** (your baby is restless, seems upset, moves around a lot, may make some noises or whimper but isn't crying; your baby is awake and uncomfortable)

/ : **sleep** (your baby is sleeping peacefully)

— : **feeding** (your baby is being fed; he or she is sucking and swallowing)

1) My baby has crying episodes

Without an apparent reason:	yes ☐	no ☐	Check the appropriate box
Inconsolable:	yes ☐	no ☐	Check the appropriate box
Accompanied by other signs: agitation and/or flushed face and/or clenched fists and/or extended legs:	yes ☐	no ☐	Check the appropriate box
**2) It was a typical day:**	yes ☐	no ☐	Check the appropriate box

It was not a typical day because: ......

Describe any significant events or unusual behavior observed in your baby.
