# Supplementary material for: Beyond crying: Protocol for a multimodal assessment of suspected prolonged pain in infants using infrared thermal imaging, behavioral assessment and physiological biomarkers (NEOTHERM)
Source: PLoS One. 2026 Nov 12;21(11):e0361344. doi: 10.1371/journal.pone.0361344 (PMC13759114; doi:10.1371/journal.pone.0361344)
Supplement: Supplementary file 3 [file pone.0361344.s003.pdf]

NEOTHERM Study

Assessment of Prolonged Pain in Infants with Excessive Crying: The Role of Thermal Imaging – NEOTHERM Pilot Study

This research is being conducted under the supervision of Dr. Alizée Lori, Head of the Neonatology Department at Saint-Denis Hospital, and Ms. Camille Perrolet, osteopath; Ms. Joëlle Provasi, Associate Professor at the EPHE; and Ms. Isabelle Carchon, Research Engineer and Ph.D. in Cognitive Psychology (CHArt),
Saint-Denis Hospital, Delafontaine Hospital, 93200 Saint-Denis
Email:coordination.rechercheclinique@ghtpdfr.fr Phone: 01 42 35 61 40 ext. 6962

INFORMATION SHEET
(for PARENTS OR GUARDIANS of INFANTS)

Dear Sir or Madam,

Dr. Lori and Ms. Camille Perrolet, Ms. Joëlle Provasi, and Ms. Isabelle Carchon are inviting your child to participate in a research study titled NEOTHERM, which aims to assess pain in infants who cry repeatedly and for prolonged periods.

Before agreeing to your child's participation, it is important to take the time to read, understand, and carefully consider the following information. This information sheet describes the purpose of the research project, how it will be conducted, and its benefits and limitations. It also describes the alternative procedures that are available and clarifies your right to withdraw your child from the study at any time.
If you decide to have your child participate in this research, you will be asked to provide written informed consent.

This document may contain words you do not understand. Please feel free to ask Ms. Perrolet or her colleagues for further clarification.

If you decide not to have your child participate in this research, their care will remain unchanged, and no data will be collected for this study.
Your child will then be cared for under the same conditions as usual, by the same medical team.

1) What is the purpose of this research?

Chronic pain is now well studied in adults, but many questions remain to be answered regarding young infants: How can this pain

be characterized? Is there a link between pain and excessive crying? Does pain affect a baby's motor development?

This research aims to better understand these phenomena.

The objective will be to compare the signs of pain and motor development in a group of infants who cry excessively (more than 3 hours per day)—the experimental group—with those of a group of infants who do not cry excessively (less than 2 hours per day), the control group. An innovative tool, thermal imaging, will be used to try to detect pain in the infants.

2) <u>What does the research involve?</u>

Participation in the study is voluntary; you may withdraw your child from the study at any time.
If your child takes part in this study, they will be cared for in the same way as in the course of their regular care.

However, one enrollment visit and two additional follow-up visits, each lasting about one hour, will be scheduled.

If your child meets the criteria for participating in the study, and if you agree, Ms. Perrolet or one of her colleagues will contact you to explain how the study will proceed, answer your questions, and schedule an appointment for an enrollment visit. You will have at least 3 days to consider your options between receiving this information and the screening visit, so that you can decide whether your child will participate. This may be extended if necessary.

During the intake visit, Ms. Perrolet or one of her colleagues will once again explain in detail the study protocol and the specifics of participation. Together, you will review this information sheet as well as the informed consent form; Ms. Perrolet or one of her colleagues will take the time to answer all your questions, and will ask you to sign the consent form.
Once the consent form has been signed by both parties, you will be asked questions about the medical details of your pregnancy and your child's birth, as well as the impact of your baby's crying (ColiQ Questionnaire).
At the end of this intake visit, you will agree on the date of the first visit (Appointment 1), and the researcher will give you a crying log: you will be asked to fill it out (recording your baby's crying and sleeping hours) during the seven days leading up to this first visit.

During the first visit (Appointment 1), the researcher will collect the completed crying diary, and your child will participate, in your presence, in an assessment designed for infants, structured in two parts:

A pain assessment:

- Your child will be filmed by a thermal camera, which records the temperature of the face and body. Thermal imaging is harmless, painless, and quick to use.
- Ms. Perrolet or one of her colleagues will use a questionnaire to assess your child's pain and discomfort (Neonatal Pain and Discomfort Scale: EDIN).
- A painless sample of a small lock of hair will be collected to measure cortisol levels, the primary stress hormone. This sample will not include your child's identity, but rather a code number assigned for the duration of the study. It will be sent to the Biological Resources Platform (PRB) at Robert Debré University Hospital, 48 bd Sérurier, 75019 Paris, for analysis.

And a motor skills assessment:

- A **short 5-minute video** will be recorded to analyze your child's spontaneous movements (infant in a onesie, without interaction with their environment).
- Their overall motor development will be assessed using the **standardized Bayley-4 test** (a tool used to observe a baby's overall development).

At the end of the first visit, you will be asked to film your child's spontaneous movements 15, 30, and 45 days after this initial appointment. You will receive detailed instructions on the filming procedure both verbally and in writing, and we will send you a reminder via text message before each scheduled date. You will be given a second crying diary to complete seven days before the second visit (Appointment 2).

The second visit will be scheduled 2 months after the first and will proceed in the same manner as Appointment 1.

The results obtained from the thermal and digital images, hair samples, and Bayley-4 assessment, together with information collected from the medical record (including your child's medical and surgical history) and information regarding their current health status (clinical examinations, results of additional tests, and concurrent treatments), will be analyzed for this research.

To ensure confidentiality and protect your child's personal data, your child's identity will remain confidential. The code number assigned to your child for this study will be used with their data for research purposes. Only the research team will have access to your child's identity and medical records. Every measure will be taken to ensure the confidentiality of participating children.

3) <u>What data will be collected?</u>

As part of this research, we will use your child's medical data, which we will collect and record in a study-specific observation log.

Specifically, we will record your child's date of birth, weight, height, medical and surgical history, treatment history, most recent imaging results, and conditions at birth and in utero.

Other data collected will include:

- Characteristics of their crying and its effects (ColiQ questionnaire)
- Results from the Neonatal Pain and Discomfort Scale (EDIN)
- Thermal imaging assessments (2 min from the front, 2 min from the back)
- Digital video assessments for analysis of general movements (5 min)
- Results of hair samples (cortisol levels)
- Results from the Bayley-4 Developmental Scale

This data collection will be used to conduct statistical analyses to confirm or refute the hypotheses formulated.

4) What is the research timeline?

The study will last 22.5 months: 20 months to recruit approximately 80 infants (40 in the control group and 40 in the experimental group) at the Saint-Denis Hospital Center, the Maison du Bébé in Saint-Denis, and the CMP on Rue Brantôme.

Your child's participation is estimated to last 2.5 months (after we have obtained your consent for their participation in this study).

5) What are the benefits and challenges associated with your child's participation in this research?

Your participation will help advance research on a topic that has been studied very little, in order to better understand pain in infants and treat it more effectively.
We aim to deepen our understanding of how pain affects infant development, with the goal of developing early preventive interventions.

6) What are the foreseeable risks and challenges associated with this research?

No significant risks have been identified in connection with this study.

The constraints identified are as follows: one enrollment visit and two additional follow-up visits, three video recordings to be made at home (on Days 15, 30, and 45 between Appointments 1 and 2), and two crying diaries to be completed at home during the 7 days preceding Appointments 1 and 2.

Whenever possible, the research visits will coincide with your child's regular follow-up appointments to save you an extra trip. If this is

not possible, your travel expenses will be reimbursed upon presentation of a receipt.

7) <u>If your child participates, what will happen to the data collected for the research?</u>

As part of the research in which you are being asked to have your child participate, the Centre Hospitalier de Saint-Denis—the research sponsor and data controller—will process your child's personal data in order to analyze the results.

To ensure confidentiality and protect your child's personal data, your child's identity will remain confidential. The code number assigned to your child for this study will be used in conjunction with their data for research purposes. Only medical staff, Ms. Perrolet, and her associates will have access to your child's identity. All measures will be taken to ensure the confidentiality and anonymity of participants.

Medical data regarding your child, collected during visits, will be made available to Camille Perrolet, Joëlle Provasi, and Isabelle Carchon in France, under the supervision of Dr. Lori, a neonatologist at the Centre Hospitalier de Saint-Denis (this data will be **<u>pseudonymized</u>** using a code number). They will not have access to your child's medical record.

At the end of this study, the data will be retained for 2 years after publication of the final report in order to fulfill the study objectives and publish the research results, after which they will be destroyed. The research documents will be archived for 15 years at the Clinical Research Unit of the GHT Plaine de France (Saint-Denis and Gonesse Hospitals), in accordance with applicable regulations. At the end of this period, they will be destroyed.

Footage recorded by thermal cameras and video footage taken at home will be stored at the Clinical Research Unit (URC) of the GHT Plaine de France until the end of the study, after which it will be destroyed.

Your child's data may be used for future research or analyses complementary to this study in collaboration with the same partners in France, under conditions that ensure confidentiality, **if you give your consent on the consent form.**

You may object at any time to the future use of your child's data by contacting the physician treating your child as part of this study or the Clinical Research Unit of the GHT Plaine de France (coordination.rechercheclinique@ghtpdfr.fr)

If you decide to withdraw your child from the research, the data collected prior to withdrawal will be used in accordance with regulations and exclusively for the purposes of this research. Deleting this data could compromise the validity of the research results. In this case, your child's data will not be used at all in the future or for any other research.

8) How is this research regulated?

Dr. Alizée Lori, the principal investigator, along with Ms. Perrolet and her colleagues, have taken all necessary measures to conduct this research in accordance with the provisions of Law 78-17, known as the “Data Protection Act,” dated January 6, 1978, the General Data Protection Regulation (GDPR), and the Public Health Code, as applicable to research involving human subjects.

The Saint-Denis Hospital Center, as the data controller, is committed to adhering to the MR001 reference methodology to fulfill a public interest mission, in accordance with Article 6 of the GDPR.

The principal investigator has received a favorable opinion from the Comité de protection des personnes Île-de-France II (CPP IDF II) to conduct this study and has taken out insurance covering the civil liability of the investigator and all persons involved in the research with LLOYD'S INSURANCE COMPANY SA, 8 rue Lamennais, 75008 PARIS, under policy number B1339CTLICNWL24-54.

9) What are your rights?

Participation in this research is entirely voluntary.
You may withdraw your child from this study at any time, without having to provide a reason. Simply inform your child's doctor or contact the Clinical Research Unit at the following address: coordination.rechercheclinique@ghtpdfr.fr
Your decision will in no way affect the quality of care and treatment your child is entitled to receive.

If you encounter any difficulties in exercising your rights, you may contact the Data Protection Officer at the Saint-Denis Hospital Center by email at dpo@ghtpdfr.fr or by mail:

mail: Saint-Denis Hospital,
Clinical Research Unit (Attn: DPO) 2 rue du Dr
Delafontaine
93200 SAINT-DENIS

The Data Protection Officer will handle your request in coordination with the physicians and professionals involved in the study. In this case, your child’s identity (first name, last name) will be disclosed to the Data Protection Officer at the Saint-Denis Hospital Center.

The Data Protection Officer can also explain the avenues for appeal available to you through the CNIL.

You may exercise your right to file a complaint directly with the CNIL (www.cnil.fr), by calling 01.53.73.22.22, or by writing to the following address:

Commission Nationale de l'Informatique et des
Libertés 3 place de Fontenoy
TSA 80715
75334 PARIS CEDEX 07

Throughout the study and upon its completion, you may request information regarding your child's health as well as explanations about the progress of the study at the doctor treating your child as part of the study, or directly to Ms. Perrolet and her colleagues.
You may object to the use of your child's data at any time during the study without providing a reason, and this will have no impact on their care, the quality of the care they receive, or their relationship with their doctor. Following withdrawal, your child may continue to be treated by the same medical team. In this case, the data collected up until the time of withdrawal will be used to analyze the research results.

The computer database used for this research is managed in accordance with the French Data Protection Act (CNIL—Law 78-17 of January 6, 1978, as amended). You You have the right to access, correct, and delete your data. You also have the right to object to the transfer of data covered by professional confidentiality that may be used and processed in the context of this research. These rights may be exercised by contacting the Data Protection Officer (dpo@ghtpdfr.fr).

You may also access all of your child's data directly or through a physician of your choice, pursuant to Article L 1111-7 of the Public Health Code.

At the conclusion of the research and after analysis of the data related to this research, you may be informed of the overall results through the physician caring for your child as part of this study, or by contacting Ms. Perrolet and her colleagues.

Assessment of Prolonged Pain in Infants with Excessive Crying: The Role of Thermal Imaging – NEOTHERM Pilot Study

This research is being conducted under the supervision of Dr. Alizée Lori, Head of the Neonatology Department at Saint-Denis Hospital and Ms. Camille Perrolet, osteopath; Ms. Joëlle Provasi, Associate Professor at the EPHE; and Ms. Isabelle Carchon, Research Engineer and Ph.D. in Cognitive Psychology (CHArt), Saint-Denis Hospital, Delafontaine Hospital, 93200 Saint-Denis

Email:coordination.rechercheclinique@ghtpdfr.fr Phone: 01 42 35 61 40 ext. 6962

RESEARCH PARTICIPATION CONSENT FORM (for PARENTS OR LEGAL GUARDIANS of INFANTS)

I , the undersigned, (Last Name, first name)<br>……………………………………….........<br><br>☐ Legal Representative 1<br>☐ Guardian(s)/conservator(s)	I , the undersigned, (Last Name, first name)<br>……………………………………….........<br><br>☐ Legal Representative 2<br>☐ Guardian(s)/Conservator(s)

of the child (Last name, first name) ……………………………………........freely consents to the child's participation in the research study titled "Assessment of Prolonged Pain in Infants with Excessive Crying: The Contribution of Thermal Imaging," organized under the supervision of Dr. Alizée Lori at the Saint-Denis Hospital Center (CHSD) by Ms. Perrolet and her colleagues (Ecole Pratiques des Hautes Etudes, a partner of the CHSD for this project).

- I have read the information sheet, Version 2, dated February 26, 2026 (7 pages), which explains the objective of this research, how it will be conducted, and what my participation will entail,
- I will keep a copy of the information sheet and the consent form,
- I have received satisfactory answers to all my questions and have had sufficient time to make my decision,
- I understand that participation is voluntary and that I may withdraw at any time without incurring any liability or compromising the quality of care provided to my child,
- I have been informed that the data collected as part of this research may be reused for future research, and that I may object to this at any time,
- I understand that, in order to participate in this research, my child must be enrolled in a social security program or be a beneficiary of such a program. I confirm that this is the case,
- I have been fully informed that my child's participation in this research will last two and a half months, and that there will be two additional visits required for this project, as well as three video recordings of my child that I will need to make at home and two crying diaries to complete,
- I have been informed that the hair sample collected for the research will be sent to Robert Debré Hospital for cortisol testing and will then be destroyed at the end of this study,
- My consent in no way relieves the physician treating my child as part of this research, nor the hospital, of any of their responsibilities, and I retain all rights guaranteed by law.

<table>
<tr>
<td>I agree to my child's participation in the NEOTHERM research study:
☐ Yes ☐ No [check the box] I agree to have their data reused for future research:
☐ Yes ☐ No [check the box]

☐ Legal representative 1
☐ Guardian(s)/Conservator(s)

Date:
Signature:</td>
<td>I consent to my child participating in the NEOTHERM study:
☐ Yes ☐ No [check the box] I consent to their data being reused for future research:
☐ Yes ☐ No [check the box]

☐ Legal Representative 2
☐ Guardian(s)/Conservator(s)

Date:
Signature:</td>
</tr>
<tr>
<td colspan="2"><u>Person Authorized to Conduct the Research</u>:
Last Name,
First Name:
Date:
Signature:</td>
</tr>
</table>

This document is prepared in duplicate.
One copy must be retained by the inclusion center; the second must be given to the research participant's legal representative.
