# Supplementary material for: Beyond crying: Protocol for a multimodal assessment of suspected prolonged pain in infants using infrared thermal imaging, behavioral assessment and physiological biomarkers (NEOTHERM)
Source: PLoS One. 2026 Nov 12;21(11):e0361344. doi: 10.1371/journal.pone.0361344 (PMC13759114; doi:10.1371/journal.pone.0361344)
Supplement: Supplementary file 4 [file pone.0361344.s004.pdf]

Appendix 5 – Questionnaire A: Socio-demographic and Medical Data

Date:

Research ID: ……./……/…….

1. Administrative Information

- **Child**

Last Name: First Name:

Date of birth: Gender:

Age: Corrected age:

- **Mother**

Last Name: First Name:

Occupation (socio-professional category): Address:

Phone:

Email:

- **Father**

Last Name: First Name:

Occupation (socio-professional category): Phone:

Email:

- **Siblings**

Number:

Ages:

- **Comments**

2. Obstetric and Medical History

- **Maternal:**

Previous pregnancies:

- Number of pregnancies:
- Parity (number of deliveries after 22 weeks of

gestation): Single or multiple pregnancy:

Maternal age:

Pre-existing medical conditions:

Pregnancy-related conditions:

Pain: location:

Duration (in days):

VAS on a scale of 10:

Smoking: yes / no

Treatments received during pregnancy:

- Medications:
- Other:

Stress level during pregnancy: EVA on a scale of 10:

- **Pediatric**

Medical conditions:

3. Birth Data

- Gestational age at birth: GA (gestational age in weeks)
- Type:
- Vaginal delivery: induced / spontaneous
- Cesarean section: elective/emergency
- Complications:
- Instruments used:
- Duration (in min):
- Labor:
- Delivery:
- APGAR:
- Measurements:
- Weight: g
- Height: cm
- Head circumference: cm
- Care received:
- Other:

4. Child's medical history

- Medical:

- Surgical:

- Treatments received:
- Medications:
- Other:

5. General condition (according to crying log)

- Crying: average hours per day

- Feeding:
- bottle-fed/breastfed/mixed
- Possible issues:

- Sleep: hours per day on average
- Average number of times awake per night:
- Number of naps:

Notes
