# Supplementary material for: Beyond crying: Protocol for a multimodal assessment of suspected prolonged pain in infants using infrared thermal imaging, behavioral assessment and physiological biomarkers (NEOTHERM)
Source: PLoS One. 2026 Nov 12;21(11):e0361344. doi: 10.1371/journal.pone.0361344 (PMC13759114; doi:10.1371/journal.pone.0361344)
Supplement: Supplementary file 5 [file pone.0361344.s005.pdf]

ROI	Anatomical region	View	Corresponding ROIs	Expected physiological ΔT
1–2	Upper thorax	Anterior	Right–Left	< 0.4 °C
3–4	Upper abdomen	Anterior	Right–Left	< 0.5 °C
5–6	Lower abdomen	Anterior	Right–Left	< 0.5 °C
7–8	Upper arm	Anterior	Right–Left	< 0.4 °C
9–10	Forearm	Anterior	Right–Left	< 0.5 °C
11–12	Hand (palmar surface)	Anterior	Right–Left	< 0.5 °C
13–14	Upper dorsal region	Posterior	Right–Left	< 0.5 °C
15–16	Lumbar region	Posterior	Right–Left	< 0.5 °C
17–18	Upper arm	Posterior	Right–Left	< 0.4 °C
19–20	Forearm	Posterior	Right–Left	< 0.5 °C
21–22	Hand (dorsal surface)	Posterior	Right–Left	< 0.5 °C
23–24	Thigh	Anterior	Right–Left	< 0.4 °C
25–26	Leg	Anterior	Right–Left	< 0.5 °C
27–28	Foot (dorsal surface)	Anterior	Right–Left	< 0.6 °C
29–30	Thigh	Posterior	Right–Left	< 0.4 °C
31–32	Leg	Posterior	Right–Left	< 0.5 °C
33–34	Foot (plantar surface)	Posterior	Right–Left	< 0.6 °C
35–36	Occipital region	Posterior	Right–Left	< 0.4 °C

S5 Table. Whole-body regions of interest (ROIs) used for infrared thermal imaging analysis. ROI numbers correspond to paired right and left homologous anatomical regions. Expected physiological ΔT values represent reference temperature differences between homologous contralateral regions reported in the pediatric thermography literature. N/A indicates that no reference threshold was identified in the available literature.
