# Supplementary material for: Beyond crying: Protocol for a multimodal assessment of suspected prolonged pain in infants using infrared thermal imaging, behavioral assessment and physiological biomarkers (NEOTHERM)
Source: PLoS One. 2026 Nov 12;21(11):e0361344. doi: 10.1371/journal.pone.0361344 (PMC13759114; doi:10.1371/journal.pone.0361344)
Supplement: Supplementary file 6 [file pone.0361344.s006.pdf]

Facial ROI code	Anatomical region	Side	Expected physiological ∆T
ROIv1	Central forehead (reference region)	Midline	± 0.2 °C
ROIv2	Nasal dorsum	Midline	± 0.3 °C
ROIv3-ROIv4	Periorbital regions	Right-Left	± 0.3 °C
ROIv5	Perioral region	Midline	± 0.3 °C
ROIv6	Mental region (chin)	Midline	± 0.3 °C

S6 Table. Facial regions of interest (ROIs) used for infrared thermal imaging analysis and expected physiological temperature variations. Facial ROIs were selected based on previous infant thermography studies and include regions in which thermal variations have been associated with autonomic and emotional responses. The central forehead (ROIv1) serves as the reference region for calculating intra-facial thermal gradients, allowing comparison of thermal patterns potentially associated with stress, discomfort, or prolonged pain.
