# Supplementary material for: Beyond crying: Protocol for a multimodal assessment of suspected prolonged pain in infants using infrared thermal imaging, behavioral assessment and physiological biomarkers (NEOTHERM)
Source: PLoS One. 2026 Nov 12;21(11):e0361344. doi: 10.1371/journal.pone.0361344 (PMC13759114; doi:10.1371/journal.pone.0361344)
Supplement: Supplementary file 7 [file pone.0361344.s007.pdf]

S8-NEOTHERM eCRF

Assessment of prolonged pain in infants with excessive crying: Contribution of infrared thermal imaging – NEOTHERM Pilot Study

1. Inclusion Visit

a) General Information

Date of inclusion visit: __/__/____
Study center (drop-down): Neonatology CH Saint-Denis; Pediatrics CH Gonesse; Pediatric Emergency Department CH Gonesse; Perinatal Psychiatry CH Saint-Denis; Brantôme Community Mental Health Center
Infant sex: ☐ Male ☐ Female
Date of birth: __/__/____
Corrected age: ___ weeks ___ days (automatic calculation).

b) Eligibility Criteria

Inclusion criteria

Corrected age 2–6 weeks: ☐ Yes ☐ No
ROME IV criteria met: ☐ Yes ☐ No
Crying duration criterion met: ☐ Yes ☐ No
No known neurological or gastrointestinal disease: ☐ Yes ☐ No

If any response is 'No', the participant is not eligible.

Non-inclusion criteria

Born before 32 weeks gestation: ☐ Yes ☐ No
Documented brain lesion (MRI/ultrasound): ☐ Yes ☐ No
Major congenital or chromosomal anomaly: ☐ Yes ☐ No
Growth failure: ☐ Yes ☐ No
Participation in another interventional study: ☐ Yes ☐ No
Participation refused by one parent or legal guardian: ☐ Yes ☐ No
No health insurance coverage: ☐ Yes ☐ No

If any response is 'Yes', the participant is not eligible.

c) Information and Informed Consent

Date information provided to parents or legal guardians: __/__/____
Date written informed consent obtained: __/__/____
Automatic assignment of the study identification number.

d) Visit 1 Scheduling

Crying diary provided: ☐ Yes ☐ No
Planned Visit 1: __/__/____

2. Visit 1

Date visit 1 : : __/__/____

a) Questionnaires

Pregnancy / Maternal Data

Maternal age: __ years
Parity: __ pregnancy
Pregnancy: ☐ Singleton ☐ Multiple
Active smoking: ☐ Yes ☐ No
Stress during pregnancy: ☐ Yes ☐ No
Pre-existing medical condition: ☐ Yes ☐ No
Pregnancy-related condition: ☐ Yes ☐ No
Pain during the third trimester: VAS 0–10 (0 = no pain; 10 = worst pain imaginable)
Medication use during pregnancy: ☐ Yes ☐ No
Drug use during pregnancy: ☐ Yes ☐ No
Comments:

Pregnancy / Infant Data

Gestational age: __ weeks + __ days
In utero pathology: ☐ Yes ☐ No
Comments:

Birth

Delivery: ☐ Vaginal ☐ Cesarean section
If vaginal: ☐ Spontaneous ☐ Induced
If cesarean: ☐ Elective ☐ Emergency
Instrumental delivery: ☐ Yes ☐ No
Complications: ☐ Yes ☐ No
Labor duration: __ hours
Expulsion stage: __ minutes
APGAR score: __
Length: __.__ cm
Birth weight: ____ g

Head circumference: __.__ cm
Feeding: ☐ Breastfeeding ☐ Bottle-feeding ☐ Mixed

Medical History

Medical history: ☐ Yes ☐ No
Surgical history: ☐ Yes ☐ No
Current treatment(s): ☐ Yes ☐ No

ColiQ Questionnaire

Infant Well-being Score (0–100)
Parent Well-being Score (0–100)
Overall ColiQ Score = mean of Infant + Parent scores (0–100)

EDIN Scale

Facial expression: 0 Relaxed face; 1 Occasional grimacing; 2 Frequent/marked/prolonged grimacing; 3 Persistent facial tension, fixed or cyanotic face (score 0–3)
Body movements: 0 Relaxed; 1 Transient restlessness; 2 Frequent agitation with possible calming; 3 Persistent agitation, limb stiffness or very limited movements (score 0–3)
Sleep: 0 Falls asleep easily; 1 Difficulty falling asleep; 2 Frequent spontaneous awakenings; 3 No sleep (score 0–3)
Interaction: 0 Attentive/smiles; 1 Brief apprehension on contact; 2 Difficult contact, cries with stimulation; 3 Refuses contact, inconsolable crying without stimulation (score 0–3)
Consolability: 0 No comforting needed; 1 Calms quickly; 2 Difficult to console; 3 Inconsolable (score 0–3)

Total EDIN score: __ (automatic calculation)

Crying Diary – Period 1

Diary completed: ☐ Yes ☐ No
Crying duration (Days 1–7): ___ min/day
Mean crying duration/day over 1 week: ___ min
ROME IV criteria: unexplained crying ☐ Yes ☐ No; inconsolable ☐ Yes ☐ No; associated symptoms ☐ Yes ☐ No
Number of ROME IV criteria: 0 / 1 / 2 / 3
Sleep duration (Days 1–7): ___ min
Feeding difficulties: ☐ Yes ☐ No
Study arm: ☐ Experimental group ☐ Control group

b) Pain and Stress Assessment

Infrared Thermal Imaging

Thermal recording performed using the infrared camera.
Body Regions of Interest (ROI): 36 predefined regions (ROI01–ROI36).

Mean temperature recorded for each ROI (T_ROIx).
Calculated thermal indices:
ΔT bilateral: absolute right–left temperature difference for homologous regions.
ΔT CP (core-to-periphery): proximal–distal temperature difference for the same side.

Body Thermal ROIs

ROI	Anatomical region	View	Side	Variables
ROI01–02	Upper thorax	Anterior	Right / Left	Mean T, ΔT bilateral, ΔT CP
ROI03–06	Upper & lower abdomen	Anterior	Right / Left	Mean T, ΔT bilateral, ΔT CP
ROI07–12	Upper limbs	Anterior	Right / Left	Mean T, ΔT bilateral
ROI13–22	Back & upper limbs	Posterior	Right / Left	Mean T, ΔT bilateral, ΔT CP
ROI23–34	Lower limbs	Anterior/Posterior	Right / Left	Mean T, ΔT bilateral
ROI35–36	Occiput	Posterior	Right / Left	Mean T, ΔT bilateral

Facial Regions of Interest (ROIv)

Code	Region	Side	Variable
ROIv1	Central forehead (reference)	Midline	Reference temperature
ROIv2	Nasal dorsum	Midline	ΔT nose–forehead
ROIv3–4	Periorbital region	Right / Left	ΔT periorbital–forehead
ROIv5	Perioral region	Midline	ΔT perioral–forehead
ROIv6	Chin	Midline	ΔT chin–forehead

Hair Sampling

Hair sample collected: ☐ Yes ☐ No

Collection date: __/__/____

Hair cortisol concentration: ____ pg/mg hair ☐ Not available

c) Motor Assessment

Digital video recording for General Movements Assessment.

<u>Visit 1 (corrected age 2–6 weeks): GMOS-R (General Movements Optimal Score – Revised).</u>

Sequence subscore (0–2)
Head and trunk (0–4)
Upper limbs (0–16)
Lower limbs (0–16)
Total GMOS-R score (0–38)

Bayley-IV Scale

Cognitive Composite Score
Receptive Communication
Expressive Communication
Fine Motor
Gross Motor
Overall Bayley-IV score

d) Visit 2 Scheduling

Video recording instructions provided to parents: ☐ Yes ☐ No
Crying diary provided: ☐ Yes ☐ No
Planned Visit 2 date: __/__/____

3) Visit 2

Visit 2 date: __/__/____

(Visit 2 should take place approximately 2 months after Visit 1.)

a) Questionnaires

Medical questionnaire: update on treatments (pharmacological and non-pharmacological, including osteopathy, chiropractic care, psychomotor therapy, psychology, and other therapies), hospitalizations, and infant medical conditions.
ColiQ questionnaire: same as Visit 1.
EDIN scale: same scoring procedure as Visit 1.
Total EDIN score: __ (automatic calculation).
Crying diary – Period 2: same procedures as Visit 1.
Diary completed: ☐ Yes ☐ No

b) Pain and Stress Assessment

Infrared thermal imaging: same acquisition protocol as Visit 1.
Hair sample collected: ☐ Yes ☐ No
Collection date: __/__/____
Hair cortisol concentration: ____ pg/mg hair ☐ Not available

c) Motor Assessment

Digital video recording: General Movements Assessment.

Visit 2 (corrected age 10–14 weeks): Motor Optimal Score (MOS).
Fidgety Movements: Normal (12) / Abnormal (4) / Absent or sporadic (1).
Other movements: Age-appropriate (4) / Reduced (2) / Absent (1).
Movement quality: Normal > Abnormal (4); Normal = Abnormal (2); Normal < Abnormal (1).
Posture: Normal > Abnormal (4); Normal = Abnormal (2); Normal < Abnormal (1).

Movement character: Smooth (4); Abnormal without cramped-synchronized movements (2); Cramped-synchronized (1).
Motor Optimal Score (MOS): total score 5–28.
Bayley-IV assessment: same as Visit 1.

Family Videos

Parents or legal guardians recorded the infant according to the study procedure: ☐ Yes ☐ No

Video sequence at Visit 1 + 15 days: __/__/____ ☐ Not available

Video sequence at Visit 1 + 30 days: __/__/____ ☐ Not available

Video sequence at Visit 1 + 45 days: __/__/____ ☐ Not available

General Movements scoring: GMOS-R up to 8–9 weeks corrected age, then MOS thereafter.
