# Supplementary material for: Beyond crying: Protocol for a multimodal assessment of suspected prolonged pain in infants using infrared thermal imaging, behavioral assessment and physiological biomarkers (NEOTHERM)
Source: PLoS One. 2026 Nov 12;21(11):e0361344. doi: 10.1371/journal.pone.0361344 (PMC13759114; doi:10.1371/journal.pone.0361344)
Supplement: Supplementary file 8 [file pone.0361344.s008.pdf]

SPIRIT 2025 checklist of items to address in a randomized trial protocol*

Section / Topic	No	SPIRIT 2025 checklist item description	Reported on page no.
**Administrative information**			
Title and structured summary	1a	**Beyond crying: Protocol for a multimodal assessment of suspected prolonged pain in infants using infrared thermal imaging, behavioral assessment and physiological biomarkers (NEOTHERM)**	1
	1b	**Primary registry and Trial identifying number** : ClinicalTrials.gov NCT07693179<br>**Secondary identifying number : IDRCB 2025-A00767-42 (French national registration)**<br>**Support** : Funded by Fondation Brou de Laurière and Fondation de l'Avenir.<br>**Primary sponsor** : Saint-Denis Hospital center.<br>**Public query contact** : Stéphanie Cossec, Clinical Research Unit, Saint-Denis Hospital center (stephanie.cossec@ghtpdfr.fr)<br>**Scientific query contact** : Camille Perrolet (camille.perrolet@etu.ephe.psl.eu)<br>**Public title** : Assessment of prolonged pain in infants with excessive crying : the role of thermal imaging (NEOTHERM).<br>**Scientific title** : Assessment of prolonged pain in infants with excessive crying versus physiological using thermal imaging : protocol for a prospective, observational, longitudinal pilot study (NEOTHERM).<br>**Country** : France<br>**Condition** : Excessive crying<br>Intervention(s) : Observation of clinical status (excessive crying group vs. physiological crying group) ; no therapeutic intervention.<br>**Criteria : Inclusion :** Infants aged 2 to 5 weeks corrected age post-term, born after 32 weeks of gestational age. Infants scheduled for the T1 assessment at 2 to 6 weeks post-term corrected age, born at or after 32 weeks of gestational age, whose legal guardians are covered by social security and have provided written informed consent. **Exclusion :** major cerebral/chromosomal abnormalities, growth faltering, birth before 32 weeks of gestational age, absence of legal guardian consent or lack of social security coverage**.**<br>**Study type** : Multicenter, prospective, observational, longitudinal pilot study<br>**First enrollment : September 2026**<br>**Sample size** : 80 infants (40 per group)<br>**Primary outcome :** Concordance between infrared thermal imaging parameters and Neonatal Pain and Discomfort Scale (EDIN) scores.<br>**Secondary outcomes :** Associations between infrared thermal imaging, hair cortisol concentration, motor organization and psychomotor development**.**<br>**Ethics reviews** : Approved by the Île-de-France Committee for the Protection of Individuals (CPP).<br>**Data sharing :** De-identified participant data will not be public but may be shared upon reasonable request to the coordinator, subject to sponsor approval and compliance with applicable regulations regarding the protection of personal data (GDPR). Access to the data will require the signing of a data-sharing agreement and the obtaining of appropriate ethical approvals.	1–4

Protocol version	2	Version No. 2, February 24, 2026.<br>Changes from version 1.0 include regulatory and methodological clarifications following Ethics Committee recommendations, including updates to investigator roles, recruitment procedures, study timeline, recruitment sites, participant follow-up, and data access.	S1 Protocol, pp. 1–2
Roles and responsibilities	3a	**List of protocol contributors** :<br>**Camille Perrolet (CP) :**<br>Affiliations : Cognitions Humaine et Artificielle (CHArt) Laboratory, École Pratique des Hautes Études (EPHE), Paris Sciences et Lettres (PSL) University, Aubervilliers, France ; Collège Européen d'Enseignement Supérieur de l'Ostéopathie (CEESO), Saint-Denis, France.<br>Roles : Conceptualized the study, designed the protocol, funding acquisition, coordinated the project and drafted the manuscript.<br><br>**Sarah Bahmd-Alves (SBA) :**<br>Affiliation : CHArt Laboratory, EPHE, PSL University, Aubervilliers, France.<br>Roles : Contributed to methodological development, study organization, data management procedures, figure preparation and critical revision of the manuscript.<br><br>**Isabelle Carchon (IC) :**<br>Affiliation : CHArt Laboratory, EPHE, PSL University, Aubervilliers, France.<br>Roles : Contributed to methodology, administrative and logistical coordination, equipment procurement, and critical revision of the manuscript.<br><br>**Juliette Berthier (JB) :**<br>Affiliation : CHArt Laboratory, EPHE, PSL University, Aubervilliers, France.<br>Roles : Contributed to the methodology, developed the thermal imaging processing pipeline and Python analysis software, generated thermal maps, conducted preliminary analyses, and contributed to manuscript drafting and revision<br><br>**Joëlle Provasi (JP) :**<br>Affiliation : CHArt Laboratory, EPHE, PSL University, Aubervilliers, France.<br>Roles : Study design, scientific methodology supervision, funding acquisition, and critical revision of the manuscript.<br><br>No artificial intelligence technologies were used in the development or writing of this protocol.	2, 30
	3b	**Trial sponsor information** :<br>Name : Saint-Denis Hospital center<br>Contact person : Stéphanie Cossec, Clinical Research Unit<br>Address : 2, rue du Docteur Delafontaine, 93200 Saint-Denis, France.<br>Phone : +33 (0)1.42.35.61.40	S1 Protocol, p. 1

		Email address : stephanie.cossec@ghtpdfr.fr	
	3c	**Roles and authority** :<br>Funders : The research foundations (Fondation Brou de Laurière and Fondation de l'Avenir) provided financial support but had no role in study design, data collection, analysis, interpretation, decision to publish or preparation of the manuscript.<br><br>Sponsor : The Saint-Denis Hospital Center, as the study sponsor, is responsible for the overall regulatory oversight of the study, including obtaining the required regulatory and ethics approvals, ensuring compliance with applicable laws and Good Clinical Practice, monitoring study conduct, managing data protection and safety reporting, and providing quality assurance throughout the study. The sponsor has no direct role in the scientific design of the study, participant recruitment, data collection, data analysis, interpretation of the results, or writing of the manuscript, which remain under the responsibility of the investigators.<br><br>Final authority : Final scientific decisions regarding the study design, conduct, data analysis, interpretation, and reporting are made by the Scientific Coordinating Investigator (Joëlle Provasi) in collaboration with the project lead (Camille Perrolet) and the study investigators.<br><br>Manuscript review : Neither the sponsor nor the funders review, comment on, or approve the manuscript prior to its submission for publication.<br><br>Mitigation of influence : To mitigate any potential influence, it is explicitly stated that funders have no direct involvement in the trial. All authors declared that no competing interests exist.	2–3
	3d	**Organizational structure and oversight** :<br>Coordinating site : The CHArt Laboratory (EPHE, PSL) serves as the coordinating center. Joëlle Provasi (scientific coordinator) and Camille Perrolet (project leader) are responsible for scientific oversight, protocol design, and multi-site coordination.<br><br>Trial management group : This group is composed of the core research team (C. Perrolet, J. Berthier, S. Bahmd-Alves, I. Carchon and J. Provasi). The trial management group is responsible for the day-to-day conduct of the trial, including recruitment at the three participating centers (Department of Neonatology, Centre Hospitalier de Saint-Denis; Department of Perinatal Psychiatry, Centre Hospitalier de Saint-Denis; and Centre Médico-Psychologique Périnatalité et Petite Enfance Brantôme, Paris), infants' assessments, and the processing of thermal and motor imaging data.<br><br>Data management team : Data management is performed internally by the research team. Specifically, C. Perrolet, J. Berthier and S. Bahmd-Alves are responsible for de-identified data entry, data management procedures and figure preparation. Oversight of data treatment is ensured by S. Cossec (sponsor	12, 25–26

		representative and data controller at the Clinical Research Unit). Data are stored on secure, HDS-certified servers (health data hosting) in compliance with General Data Protection Regulation (GDPR).<br><br>Trial monitoring : A risk-proportionate monitoring plan is implemented by the Clinical Research Unit of the Saint-Denis Hospital center (sponsor). This includes site setup visits, quality control of the informed consent process, verification of eligibility criteria, and auditing the consistency between source data and the electronic Case Report Form (e-CRF). The project manager at the Clinical Research Unit is Nathalie Albret.	
**Open science**			
Trial registration	4	**Name of registry** : ClinicalTrials.gov<br>**Trial registry identifying number :** NCT07693179<br>**Secondary identifying number :** IDRCB 2025-A00767-42 (French national registration)<br>**URL to registry record :** https://clinicaltrials.gov/study/NCT07693179<br>**Date of registration :** July 9, 2026	4
Protocol and statistical analysis plan	5	**Accessibility of protocol and statistical analysis plan** :<br>**Protocol:** The full study protocol is available as Supporting Information accompanying this publication and will also be accessible through the ClinicalTrials.gov registry once the registration record is published. The SPIRIT checklist is provided as Supporting Information.<br>**Statistical Analysis Plan (SAP):** The main statistical methods are described in this protocol. A detailed Statistical Analysis Plan will be finalized before database lock and prior to outcome analyses.	20–21; S1 Protocol, pp. 40–43
Data sharing	6	**Shared data and materials:** De-identified individual participant data underlying the published results, the data dictionary, and study-related materials (including the study protocol, statistical analysis plan, and case report forms) will be made available for legitimate scientific research, subject to the conditions described below.<br><br>**Non-shared materials:** Identifiable participant information, source documents, signed informed consent forms, thermal images containing potentially identifiable facial features, and any other personal data protected under applicable data protection legislation will not be shared.<br><br>**Access process:** Requests for access should be submitted to the study coordinator. Access will be considered on a case-by-case basis by the investigators and is subject to approval by the study sponsor (Saint-Denis Hospital Center), in accordance with institutional policies and applicable regulations.<br><br>**Transfer conditions:** Access to shared data requires the signing of a data-sharing agreement and compliance with the General Data Protection Regulation (GDPR) and applicable French regulations. Where required, the requesting institution must provide evidence of appropriate ethical and regulatory approvals.	3, 26

		**Participant consent:** Written informed consent obtained from the holders of parental responsibility explicitly includes the use of de-identified research data for scientific research. Any data sharing will be conducted in accordance with the consent provided and applicable legal and ethical requirements.<br><br>**Rationale for restricted access**: Individual participant data will not be publicly available because the study involves infants, a vulnerable population, and includes sensitive clinical information and facial thermal images that may carry a residual risk of re-identification despite pseudonymization. Controlled access is therefore necessary to protect participant confidentiality and to comply with ethical and regulatory requirements	
Funding and conflicts of interest	7a	**Direct monetary support** : This study is directly funded by two research foundations : Fondation Brou de Laurière and Fondation de l'Avenir.<br><br>**Indirect support (services and technical assistance)** : The Clinical Research Unit of the Saint-Denis Hospital center provides methodological support, regulatory coordination, and monitoring services. The Biochemistry and Endocrinology Laboratory of the Robert Debré Hospital (AP-HP) provides the complete specialized service for hair cortisol analysis, including the HPLC-MS/MS processing and the transmission of analytical results to the research team.	2–3
	7b	**Conflict of interest statement** :<br>Declaration : The authors and all members of the research team declare that no competing financial or non-financial interests exist regarding the design, conduct, or reporting of this study.<br><br>Financial interest : No investigator has received salary support, grants (beyond the study funding mentioned), honoraria for authorship or public speaking, or paid consultancy from organizations with a potential interest in the study outcomes. No author holds patents or patents pending related to the infrared thermal imaging software or procedures described in this protocol.<br><br>Non-financial interests : There are no personal or professional relationships or academic commitments that could influence the trial's integrity.<br><br>Mitigation procedures : To prevent any potential influence, it is explicitly established that the funding organizations (Fondation Brou de Laurière and Fondation de l'Avenir) have no role in study design, data collection, analysis, decision to publish, or preparation of the manuscript. All primary and secondary outcome assessments will be performed by investigators blinded to the group allocation to further ensure objectivity.	3
Dissemination policy	8	**Scientific dissemination** : Results will be communicated to healthcare professionals through publications in scientific journals (including the publication of this protocol) and oral presentations at national and international conferences. The trial results will also be reported in the ClinicalTrials.gov registry.	26

		**Communication to participants and the public** : At the conclusion of the study, a summary of the collective results will be made available to the public and families on the NEOTHERM website (https://sites.google.com/view/neotherm-fr). Furthermore, individual qualitative feedback on motor and behavioral observations will be provided to the parents when relevant to the child's care. Public-facing materials and explanatory brochures will also be used for broader communication.<br><br>**Process and timeframe :** Recruitment is planned from September 2026 to September 2028, with study completion and initial results expected in November 2028. The scientific supervisor (J. Provasi) will sign the final trial report, confirming agreement with the analyses and conclusions.<br><br>**Authorship guidelines** : Authorship will follow the International Committee of Medical Journal Editors (ICJME) recommendations. To be designated as an author, contributors must have participated sufficiently in the study design, data analysis, or manuscript drafting and revision. The order of authors will reflect the number of participants included and the significance of the scientific contribution. All publications will explicitly mention the funding sources (Fondation Brou de Laurière and Fondation de l'Avenir).<br><br>**Ancillary studies** : Any results from secondary or ancillary studies may only be published after the primary study's results are released and must be approved by the principal investigators and the study methodologist.	
**Introduction**			
Background and rationale	9a	**Importance of the research question** : Prolonged pain in infancy is a poorly defined concept with significant risks for long-term neurodevelopmental outcomes, including alterations in pain processing, cognitive delays, and behavioral issues. Excessive crying is one of the most frequent parental concerns, affecting ~25%, yet its link to underlying prolonged pain remains insufficiently explored.<br><br>**Need for a new trial :** Current clinical assessment of neonatal pain relies primarily on indirect behavioral scales like the Neonatal Pain and Discomfort Scale (EDIN). These scales are subjective and may not fully capture the complexity of prolonged pain, especially in non-verbal populations. No objective gold-standard measure currently exists for prolonged infant pain. NEOTHERM is needed to investigate a novel, observer-independent tool to fill this diagnostic gap.<br><br>**Mechanism of action** : Infrared thermal imaging is a non-contact technique that captures skin temperature distribution, which reflects peripheral blood flow regulated by the autonomic nervous system. Pain and chronic stress induce autonomic dysregulation (sympathetic activation), leading to detectable thermal asymmetries or gradients (>0,5°C) on the face and body.<br><br>**Pre-trial evidence** : Infrared thermal imaging has demonstrated utility in neonatology for monitoring thermoregulation and stress responses. In pain research (adults and animals), thermal patterns have shown high sensitivity for detecting autonomic dysregulation associated with painful states. Facial regions (nose tip, perioral region) appear specifically sensitive to nociceptive stimuli.	5–10

		**Summary of evidence** : This study is based on a comprehensive review of existing literature on neonatal pain (e.g., Anand, 2017) and infrared thermal imaging applications (e.g., AlZubaidi, 2018). While infrared thermal imaging is validated in other contexts, to our knowledge, no previous trial has applied it to infants with excessive crying. This pilot study will generate the first variance and feasibility data required for future confirmatory trials.	
	9b	**Selection of the comparator group :** The comparator group consists of infants with physiological crying durations, defined as crying for less than 2 hours per day. This group was chosen to establish a physiological baseline for infrared thermal signatures, behavioral and motor patterns, and biological markers (hair cortisol concentration) during typical development.<br><br>**Threshold justification** : The <2-hours threshold was selected because it corresponds to the typical development trajectories described in international literature for infants between 3 and 12 weeks of age. To ensure a clear distinction between the clinical phenotype (excessive crying) and the physiological state, infants with intermediate crying patterns (2 to 3 hours per day) are excluded.	10–11
Objectives	10	**Participants :** 80 infants (n = 40 per cohort), with T1 assessment at 2–6 weeks post-term corrected age and T2 assessment at 10–14 weeks post-term corrected age.<br><br>**Intervention (exposure)** : Naturally occurring clinical status of “excessive crying”, defined as crying >3 hours per day for at least 3 days per week according to modified Wessel and Rome IV criteria.<br><br>**Comparator** : Infants with “physiological crying” durations, defined as <2 hours per day.<br><br>**Primary objective :** To evaluate the concordance (convergent validity) between infrared thermal imaging signatures and the Neonatal Pain and Discomfort Scale (EDIN) to investigate infrared thermal imaging as an objective indicator of prolonged pain.<br><br>**Secondary objectives** :<br>- To examine the relationships between infrared thermal imaging parameters, Neonatal Pain and Discomfort Scale (EDIN) scores, and hair cortisol concentration levels (biomarker of chronic stress).<br>- To describe and characterize the spontaneous motor activity (General Movements Assessment) and psychomotor development (Bayley-IV scales) of infants presenting with excessive crying.<br>- To explore the associations between atypical motor patterns, physiological and behavioral indicators of prolonged pain.<br><br>**Time point of main interest :** The primary outcome is assessed at two key developmental stages : T1 (2–6 weeks post-term corrected age) and Visit 2 (12 to 15 weeks of corrected age)T1 (2–6 weeks post-term corrected age) and T2 (10–14 weeks post-term corrected age).	10–11

		**Description of trial estimand :** As this is a prospective observational pilot study, the primary estimand is the mean degree of association (measured by Spearman correlation coefficients and Bland-Altman agreement) between facial and body thermal gradients and ~~Neonatal Pain and Distress scale~~Neonatal Pain and Discomfort Scale (EDIN) within the study population. This estimand aims to quantify the diagnostic performance of infrared thermal imaging regardless of potential variations in crying status during the 2-month follow-up.	
**Methods: Patient and public involvement, trial design**			
Patient and public involvement	11	**Who is involved** / **Involvement in trial conduct/ Involvement in reporting/**<br>**Trial design** : Patients and members of the public were not formally involved in the design of the study, selection of outcomes, development of the protocol, or preparation of the dissemination strategy. Parents of enrolled infants participate in study procedures and data collection as described elsewhere in the protocol.	N/A
Trial design	12	**Type of trial design/ unit of allocation/allocation ratio** : Multicenter, prospective, observational, longitudinal, and comparative pilot study comprising two cohorts defined by naturally occurring crying patterns. The target sample comprises 40 infants with excessive crying and 40 infants with physiological crying. No intervention is assigned and no randomization is performed.<br><br>**Conceptual framework** : This is an exploratory and descriptive pilot study (not a superiority or non-inferiority trial). It aims to generate preliminary evidence and effect-size estimates for future confirmatory studies.	10–16
**Methods: Participants, interventions, and outcomes**			
Trial setting	13	**Setting of participant recruitment** : Recruitment and data collection will occur in specialized hospital units, specifically :<br>- <u>Inpatient and tertiary care</u> : A neonatal unit and a neonatal psychiatry unit.<br>- <u>Outpatient and emergency care</u> : A medical-psychological center and a pediatric emergency service.<br><br>**Locations** : The study will be carried out in France, specifically in the Île-de-France region. The participating cities are :<br>- Saint-Denis (Saint-Denis Hospital center).<br>- Paris (Centre Médico-Psychologique Périnatalité et Petite Enfance Brantôme).<br><br>**Planned number of sites** : The trial will be conducted across three inclusion centers located within these two hospital centers.	12

		**Assessment environment** : All thermal imaging assessments will be performed in dedicated rooms that meet strict standardized environmental conditions (stable temperature of 23°C ± 1°C and humidity of 50-60%).	
Eligibility criteria	14a	**Inclusion criteria (common to both groups)** :<br>- Infants scheduled for the T1 assessment at 2–6 weeks post-term corrected age.<br>- Born at a gestational age > 32 weeks.<br>- No known underlying medical pathology.<br>- Legal guardians covered by social security.<br>- Written infrared consent provided by legal guardians.<br><br>**Group-specific inclusion criteria (based on the 7-day diary)** :<br>‘Excessive crying’ group : Crying > 3 hours per day for at least 3 days during the week preceding assessment (modified Wessel criteria), and meeting at least two Rome IV criteria (inconsolability, absence of apparent cause, or agitation).<br>‘Physiological crying’ group : Crying duration < 2 hours per day.<br><br>**Exclusion criteria** :<br>- Major cerebral or chromosomal abnormalities.<br>- Statura-ponderal stagnation.<br>- Birth before 32 weeks of gestational age.<br>- Intermediate crying patterns : crying between 2 and 3 hours per day (excluded to prevent classification bias).<br>- Lack of guardian consent or social security coverage.	11–12
	14b	**Site eligibility criteria** Participating sites must provide clinical care to the target infant population, have appropriate facilities for standardized study procedures, and ensure the availability of trained investigators authorized to conduct the study.<br><br>**Investigator eligibility criteria** : Assessments must be performed by qualified personnel as follows :<br>Clinical assessments and Pain and Discomfort Scale (EDIN) : Performed by trained clinical investigators experienced in neonatal observation.<br>Infrared thermal imaging : Data extraction is performed by a trained investigator with extensive expertise in thermography who developed the processing pipeline. A second trained investigator, blinded to group allocation and clinical results, will independently review a subset of thermal images to assess reliability of data extraction procedures.<br>General Movements Assessment : Video scoring is conducted independently by two GM Trust-certified advanced assessors.<br>Bayley-IV scales : Administered by researchers who have received specific training in the administration and scoring of this gold-standard developmental tool.	12

Intervention and comparator	15a	**Definition of groups (clinical phenotypes)** :<br>Experimental group ('intervention') : Infants with naturally occurring excessive crying > 3 hours per day for at least 3 days in the preceding week (modified Wessel criteria) and meeting at least two Rome IV criteria (inconsolability, absence of apparent cause, or agitation).<br>Comparator group ('usual care / physiological') : Infants with physiological crying durations (< 2 hours per day).<br><br>**Administration and components** : Both groups undergo identical multimodal assessment procedures :<br>Behavioral : Neonatal Pain and Discomfort Scale (EDIN) scored by trained investigators (3 min).<br>Thermal : Infrared thermal imaging using a FLIR® T630sc camera. Procedures include a 15-minutes acclimatization (held by parent), followed by 2 minutes in supine and 2 minutes in prone positions on a standardized mattress.<br>Biological : Collection of hair samples (≤ 50 milligrams) from the lower occipital region for hair cortisol concentration analysis via HPLC-MS/MS.<br>Developmental : General Movements Assessment (3 to 5 minutes video) and Bayley-IV scales of Infant and Toddler development subtests (15 minutes).<br><br>**Timing and duration** :<br>Visit 1 (T1) : 3 to 6 weeks corrected age (duration : 1 hour).<br>Home follow-up : Parents record three 3 to 5 minutes videos of their infant's General Movements at days 15, 30, and 45 post-T1.<br>Visit 2 (T2) : 10 to 14 weeks post-term corrected age (duration : 1 hour).<br><br>**Physical and informational materials** :<br>7-day crying diary : Provided to parents at enrollment and Visit 1 to record crying, sleeping, and feeding periods.<br>Instruction leaflets : Standardized guides for home video recording based on the GM Trust protocol.<br>Study website : The NEOTHERM website (https://sites.google.com/view/neotherm-fr) provides additional project details for families.<br><br>**Usual care tracking** : As NEOTHERM is observational, no therapeutic intervention is delivered. Both groups continue to receive their standard pediatric or neonatal care as prescribed by their regular healthcare providers. The study does not modify or track specific external treatments unless recorded in the sociodemographic and medical questionnaire.<br><br>**Tailoring** : No individual tailoring of the assessment protocol is planned, as procedures are strictly standardized to ensure comparability between groups.	13–20
	15b	**Criteria for modification** : As this is an observational study investigating naturally occurring clinical phenotypes, no modifications to group allocation are permitted. Group assignment established at Visit 1 (T1) based on the first 7-day diary is strictly maintained throughout the study follow-up, regardless of any	Protocol, p.27-29

		subsequent improvement or worsening of crying symptoms. This approach is intended to preserve the longitudinal integrity of the cohort and prevent outcome-driven reclassification.<br><br>**Criteria for discontinuation** :<br>The primary criterion for discontinuation is the withdrawal of consent by the legal guardians.<br><u>Parental withdrawal</u> : Parents are explicitly informed that they may withdraw their child from the study at any time, for any reason, and without having to provide a justification.<br><u>Medical safety</u> : Although the study is non-interventional and carries minimal risk (RIPH2), participation would be discontinued if a major medical condition (e.g., new diagnosis of a cerebral or chromosomal abnormality) is identified during the follow-up period, as the infant would no longer meet the core eligibility criteria.<br><u>Procedural discontinuation</u> : For individual assessment sessions (e.g., infrared thermal imaging or General Movements recording), the procedure may be temporarily interrupted if the infant presents persistent crying or agitation that cannot be soothed by the parent, in order to prioritize the participant's comfort.	
	15c	**Participant adherence (parents)** :<br><u>Information and training</u> : Parents receive an explanatory brochure, access to the study website (https://sites.google.com/view/neotherm-fr), and a standardized information leaflet to ensure they understand the requirements for the crying diary and home videos.<br><u>SMS reminders</u> : To promote the timely submission of home-recorded motor videos, automated SMS reminders are sent to parents before each measurement interval (Days 15, 30, and 45 post-Visit 1).<br><u>Standardized instructions</u> : Standardized instructional materials provide clear rules for home recordings (e.g., supine position, no pacifier, 3-minute duration) to minimize protocol deviations.<br><br>**Investigator fidelity** :<br><u>Training and certification</u> : All assessments are performed by specialized personnel. General Movements Assessments are scored by certified advanced assessors, and Bayley-IV scales are administered by researchers who have undergone specific training.<br><u>Environmental standardization</u> : To ensure the reliability of thermal imaging, assessments are strictly conducted in dedicated rooms with controlled temperature (23°C ± 1°C) and humidity (50–60%), monitored by specialized sensors.<br><u>Blinding procedures</u> : Distinct investigators are responsible for group allocation and outcome analysis to prevent observer bias.<br><br>**<u>Monitoring of fidelity and adherence :</u>**<br><u>Sponsor monitoring</u> : A risk-proportionate monitoring plan implemented by the Clinical Research Unit (Saint-Denis Hospital center) includes site setup visits and audits of source data to ensure the protocol is executed as planned.<br><u>Quality control</u> : Thermal data extraction is subject to independent review of a subset of images, and General Movements videos are rated by two independent observers with consensus-based resolution of discrepancies.	Protocol, p.28, 44-45, 110-111

		**Classification of protocol deviations** :<br>Participants are classified as being 'treated as planned' if they complete the mandatory assessment visits (Visit 1 and 2) within the pre-specified time windows (2–6 weeks and 10–14 weeks post-term corrected age).<br>As this is an observational study, group allocation is strictly maintained based on the initial 7-day crying diary, regardless of symptom fluctuations, to ensure the longitudinal integrity of the cohort analysis.	
	15d	**Recording of concomitant care:**<br>All relevant concomitant treatments and medical history are recorded during the enrolment visit using a standardized medical and sociodemographic questionnaire. This questionnaire specifically captures information on the infant's current medications, feeding modality, and any medical or supportive interventions received since birth. Any significant changes in the infant's health status, medications, feeding practices, or other concomitant treatments occurring during the two-month follow-up are documented at the follow-up visit. These variables will be considered in the interpretation of the study findings and may be included as covariates or explored in secondary analyses where considered clinically relevant.<br>Permitted concomitant care : As NEOTHERM is a prospective observational study, all forms of standard pediatric and neonatal care are permitted. Infants continue to receive any medical treatments, nutritional support, or developmental interventions prescribed by their regular healthcare providers (pediatricians, general practitioners, or hospital specialists). No 'rescue interventions' are specifically defined by the protocol as the study does not involve the administration of a trial-related therapy or the induction of pain.<br><br>Prohibited care : There are no prohibited medications or treatments during the trial period. The study is designed to observe infants in their natural clinical state and does not interfere with their routine medical management.<br><br>Recording of concomitant care :<br>All relevant concomitant treatments and medical history are recorded during the enrollment visit using a standardized medical and sociodemographic questionnaire. This questionnaire specifically captures information on the infant's current medications, feeding modalities, and any medical interventions received since birth.<br>Any significant changes in the infant's health status or new treatments during the 2-month follow-up are noted by investigators during the Visit 2 assessments.	Protocol, p28, p. 100-101
Outcomes	16	1. **Primary outcome - Convergent validity of infrared thermal imaging and the Neonatal Pain and Discomfort Scale (EDIN) :**<br>Rationale : To investigate if infrared thermal imaging can serve as an objective indicator of prolonged pain by comparing it with the current behavioral reference standard (Neonatal Pain and Discomfort Scale (EDIN)).	16–20

		Specific variable : Bilateral thermal asymmetries (>0,5°C) and facial/body temperature gradients measured by infrared thermal imaging, and total scores (0-15) on the Neonatal Pain and Discomfort Scale (EDIN).<br>Analysis metric : Degree of association calculated via Spearman correlation coefficients and agreement assessed via Bland-Altman plots.<br>Summary measure : Correlation strength (rho) with 95% confidence intervals and mean bias.<br>Time points : Visit 1 (3 to 6 weeks corrected age)T1 (2–6 weeks post-term corrected age) and T2 (10–14 weeks post-term corrected age).Time points : T1 (2–6 weeks post-term corrected age) and T2 (10–14 weeks post-term corrected age).<br><br>**2. Secondary outcome - Association with stress biomarkers**<br>Rationale : To examine the physiological burden of chronic stress associated with crying phenotypes.<br>Specific variable : Capillary cortisol concentration expressed in pg/mg of hair.<br>Analysis metric : Bivariate Spearman correlations between infrared thermal imaging parameters, Neonatal Pain and Discomfort Scale (EDIN) scores, and cortisol levels.<br>Summary measure : Median and interquartile range per group.<br>Time points : Hair sampling at T1 (2–6 weeks post-term corrected age) and T2 (10–14 weeks post-term corrected age), representing cumulative cortisol exposure.<br><br>**3. Secondary outcome - Motor and neurodevelopmental profiles**<br>Rationale : To characterize the developmental impact of excessive crying and prolonged pain.<br>Specific variable : General Movement Optimality Scores (GMOS-R for writhing; MOS-R for fidgety) and Bayley-IV subtest composite scores (Cognitive, Language, Motor).<br>Analysis metric : Between-group differences assessed using Mann–Whitney tests and longitudinal trajectories using mixed-effects models.<br>Summary measure : Median and interquartile range for motor scores, mean and standard deviation for standardized Bayley-IV scores.<br>Time points : General Movements Assessment at T1 (2–6 weeks post-term corrected age), days 15, 30 and 45 after T1, and T2 (10–14 weeks post-term corrected age). Bayley-4 at T1 and T2.	
Harms	17	**Systematically assessed harms (active surveillance)** :<br>Definition : Procedural distress or discomfort during multimodal assessments.<br>Measurement : Direct clinical observation of infant behavioral states (persistent crying, agitation, or signs of autonomic distress) by the investigators during assessment sessions.<br>Procedures : Assessments are conducted by trained clinical investigators. If an infant persistent crying that cannot be soothed by the parent or shows signs of significant discomfort, the procedure (e.g., infrared thermal imaging recording or General Movements Assessment) is temporarily or permanently interrupted to prioritize participant safety and comfort.<br>Time points : Continuous monitoring during Visit 1 and Visit 2.	19–20; S1 Protocol, p. 50

		**Non-systematically assessed harms (passive surveillance)** : Parents or legal guardians are instructed to contact the study investigators if any adverse event, unexpected clinical deterioration, hospitalization, or other safety concern occurs between scheduled study visits. All spontaneously reported adverse events considered relevant to the study will be documented in the e-CRF, assessed by the investigator, and reported to the sponsor in accordance with applicable French regulations. Participants requiring medical attention will be referred to their usual healthcare provider or the appropriate hospital service. Any serious adverse event will be managed and reported according to the sponsor's standard operating procedures and regulatory requirements.	
Participant timeline	18	SPIRIT diagram of the timeline for enrolment, assessments, and procedures in the NEOTHERM study.	13–16

	STUDY PERIOD							
	Enrolment		Cohort classification	Follow-up				Close-out
				At the family home				
TIMEPOINT	(-t) to 0	0	T1 (2-6 weeks of CA)	Day 15	Day 30	Day 45	T2 (10-14 weeks of CA)	t(_x)
ENROLMENT:								
Eligibility screen	X							
Information and consent of legal guardian	X	X						
Socio-demographic and medical data			X					
Distribution of the crying diary (7 days)		X	X (for the T2 visit)					
Cohort classification			X				X (maintained)	
COHORT CLASSIFICATION: Excessive-crying cohort ‡			◆	—	—	—	◆	X
Typical-crying cohort ‡			◆	—	—	—	◆	X
ASSESSMENTS:								
ColiQ®			X				X	
EDIN scale (prolonged pain-related behaviors)			X				X	
Infrared Thermal Imaging			X				X	
Hair cortisol sampling			X				X	
Videos of general movements			X	X	X	X	X	
Bayley-4			X				X	
DATA ANALYSIS:								
Data management and processing / security / monitoring			X	X	X	X	X	X
Statistical analysis								X
Restitution to families and publishing								X

This table shows the target time points and acceptable assessment windows. Cohort classification is based on the first 7-day crying diary completed before T1. The baseline classification will be retained for the primary analyses; no intervention is assigned.

X: event or procedure performed at the specified time.

◆—◆ continuous exposure (here: clinical status of the observed group).

‡: Cohort status reflects naturally occurring crying patterns; no therapeutic intervention is assigned.

Sample size	19	**Primary outcome for calculation :** The determination of the sample size is theoretically based on the concordance (convergent validity) between infrared thermal imaging signatures and the Neonatal Pain and Discomfort Scale (EDIN) scores.<br><br>**Assumptions and values** : Since no previous study has investigated the relationships between infrared thermal imaging, prolonged pain, and motor development in infants presenting with excessive crying, a formal sample size calculation was not feasible due to the lack of pre-existing data (such as expected proportions or means).<br><br>**Rationale for the selected size** : The sample size was determined following international methodological recommendations for pilot and exploratory studies. These guidelines suggest that a group size of 35 to 45 subjects per group is appropriate to evaluate the feasibility of the protocol, the relevance of the outcome measures, and to estimate the variance parameters required for future larger-scale confirmatory trials.<br><br>**Target difference and standard deviation** : Not applicable. As this is an exploratory pilot study, the sample size was not based on an expected effect size or standard deviation but on methodological recommendations for pilot studies.<br><br>**Significance and power** : Statistical analyses will use a significance level (type one error) set at five percent ($p < 0.05$) with a two-tailed test. A formal statistical power (type two error) was not pre-specified for this pilot phase.<br><br>**Target sample size** : The study aims to include a total of 80 infants, divided into 40 infants in the excessive crying group and 40 infants in the physiological crying group.<br><br>**Adjustments :** No formal inflation of the sample size for anticipated attrition was performed. Recruitment will continue until 40 evaluable infants have been included in each study group whenever feasible. Reasons for withdrawal and missing data will be documented.<br><br>**Software used :** No statistical software was used to calculate the sample size, as no formal power calculation was performed. The target sample size was defined based on published methodological recommendations for pilot and exploratory studies, and on feasibility considerations related to recruitment capacity across the participating centers.	11, 24
Recruitment	20	No statistical software was used to calculate the sample size, as no formal power calculation was performed. The target sample size was defined based on published methodological recommendations for pilot and exploratory studies, and on feasibility considerations related to recruitment capacity across the participating centers.<br>Information materials : Potential participants will be reached through the distribution of explanatory brochures by healthcare teams at participating centers. These brochures contain a link to the dedicated	12

		study website (https://sites.google.com/view/neotherm-fr), which provides additional project details and transparency for families.<br>Direct communication : The research team will conduct discussions with interested parents to present the study's clinical interests, address questions, and clarify the non-invasive nature of the procedures.<br>Reducing participant burden : The study design minimizes the need for frequent hospital visits by using simple assessment techniques and allowing parents to record and submit follow-up motor videos from their own home using standardized instructions.<br><br>**Recruitment locations, personnel and timing** :<br>Where : Recruitment and data collection will take place across 3 inclusion centers in the Île-de-France region, including a neonatal unit, a neonatal psychiatry unit, a medical-psychological center, and a pediatric emergency service.<br>By whom : Eligible families are initially identified and referred to the study by the clinical healthcare teams (doctors and nurses) at the participating hospital units. Formal enrolment, clinical screening, and informed consent collection are performed by the trained study investigators (coordinating or associate investigators).<br>When : Infants will be recruited and enrolled when they are between 2 and 5 weeks of corrected age (post-term). This timing is strategically chosen to establish group allocation during the peak period of infant crying (typically around 6 weeks) and to allow for a longitudinal follow-up until fifteen weeks of corrected age.	
**Methods: Assignment of interventions**			
Randomization:			
Sequence generation	21a	Not applicable. NEOTHERM is a prospective observational study. No randomization is performed and no random allocation sequence is generated. Participants are classified into the “excessive crying” or “physiological crying” group at Visit 1 according to pre-specified clinical criteria based on the 7-day crying diary and clinical assessment.	N/A
	21b	**Type of randomization :** Not applicable. As NEOTHERM is a multicenter, prospective observational pilot study, participants are not randomized. Cohort classification is determined at T1 from the 7-day crying diary using prespecified crying-duration and protocol-defined criteria.<br><br>**Factors for compatibility (pair-matching)** : To minimize confounding effects and ensure comparability between the ‘excessive crying’ group and the ‘physiological crying’ group, a pair-matching strategy is implemented.<br><br>**Matching categories** : Control infants will be matched to exposed infants based on the following pre-specified factors :<br>- Corrected age : Matched within a window of plus or minus 1 week at the time of evaluation.<br>- Sex : A one-to-one ratio (male/female) will be sought where possible.<br>- Gestational age at birth : Matched within a window of plus or minus 1 week of amenorrhea.	12

		- <u>Feeding modality</u> : Categorized as exclusive breastfeeding, exclusive formula feeding, or mixed feeding.<br>- <u>Maternal parity</u> : Categorized as primiparous or multiparous.	
Allocation concealment mechanism	22	**Nature of the mechanism / Concealment through temporal separation / Prevention of selection bias :** Not applicable. No random allocation sequence is generated in NEOTHERM; therefore, no allocation concealment mechanism is required. Group classification is based on pre-specified clinical criteria assessed at Visit 1.	N/A
Implementation	23	**Access to allocation sequence / Who will enroll participants/ Who will assign participants /Access and separation of roles / Secure storage of allocation records** : Not applicable. As NEOTHERM is an observational study, no random allocation sequence is generated or implemented. Participants are enrolled by authorized clinical investigators and are subsequently classified into study groups according to the pre-specified criteria defined in the protocol.	N/A
Blinding	24a	**Trial participants** : Not blinded. Since group assignment is determined by the parents' own recordings in a 7-day behavior diary and their report of clinical symptoms, the parents and legal guardians are inherently aware of their infant's clinical status (exposure group).<br><br>**Care providers (recruiting investigators)** : Not blinded. Participant recruitment, eligibility assessment, collection of crying diaries, and group allocation are performed by the recruiting investigators (Camille Perrolet, and Isabelle Carchon), who are therefore aware of participants' exposure status. To preserve blinding, these investigators do not participate in the analysis of thermal imaging, General Movements, or statistical outcomes. Not blinded. The trained clinical investigators responsible for the initial clinical assessment, collection of crying diaries, and determination of group allocation are aware of the participants' assignments. To minimize bias, these investigators are strictly prohibited from participating in the subsequent analysis of thermal or motor outcomes.<br><br>**Outcome assessors** : Blinded. Strict blinding is maintained for all individuals responsible for primary and secondary outcome measures :<br>- <u>Infrared thermal imaging assessors</u> : Thermal image preprocessing, extraction of quantitative thermal variables, and quality control are performed by Juliette Berthier and Sarah Bahmd-Alves, who are blinded to group allocation, EDIN scores, hair cortisol concentrations, and all clinical data.<br><u>General Movements Assessment assessors</u> : Video recordings are independently scored by two certified advanced General Movements assessors (Joëlle Provasi and Maëva Da Cunha) who are blinded to group allocation, EDIN scores, thermal imaging findings, and hair cortisol concentrations. Disagreements are resolved by consensus before the final score is entered into the study database.<br><u>Bayley-4 assessments</u> are administered by trained study investigators. Video recordings are independently rescored by a trained investigator (JB, JP or SBA) who did not administer the corresponding session and is blinded to cohort allocation.	N/A

		**Data analysts** : The statistical dataset will be pseudonymized prior to analysis to ensure that the primary data analysis is performed by the research team without direct identification of individual participants.	
	24b	**Mechanism to establish blinding :** Blinding is achieved through the use of coded recordings (thermal images and digital motor videos) and pseudonymized datasets. Assessors receive digital files labeled only with a unique participant study identifier. All clinical metadata that could reveal the group assignment (such as crying diary results or behavioral scores) is stripped from the files prior to analysis**.**<br><br>**Similarity of assessment conditions** : Although this is not an interventional trial using placebos, both clinical groups are subject to identical and standardized assessment procedures. All measurements (infrared thermal imaging, General Movements recordings, and Bayley-IV scales) are conducted in the same dedicated rooms under identical environmental conditions (stable temperature of 23°C ± 1°C and humidity of 50%–60%). This standardization ensures that the visual context of the recordings provides no environmental clues to the assessors regarding the infant's clinical phenotype.<br><br>**Procedures to maintain blinding** :<br>- <u>Strict separation of duties</u> : The coordinating and associate investigators responsible for recruitment and group allocation are strictly prohibited from participating in the subsequent analysis of thermal or motor outcomes.<br>- <u>Consensus scoring</u> : General Movements are scored independently by two certified advanced assessors. In the event of a discrepancy, a consensus is reached, which further protects against individual observer bias that might arise from accidental unblinding.<br>**Access controls:** Access to the study database is restricted through role-based permissions. Only authorized investigators listed on the delegation log have access to pseudonymized participant data required for their study responsibilities. Investigators performing infrared thermal imaging analyses and General Movements Assessments have access only to coded datasets and recordings identified by study codes, without information on group allocation or clinical outcomes. Access rights are managed by the sponsor in accordance with institutional procedures and the General Data Protection Regulation (GDPR).	25
	24c	**Circumstances for unblinding :** As NEOTHERM is an observational study investigating naturally occurring clinical phenotypes (excessive vs. physiological crying). The infant’s crying pattern is known to the legal guardians, while formal cohort classification is established by the clinical investigators at T1 after review of the 7-day crying diary. Consequently, traditional emergency unblinding for medical safety is not applicable here, as the ‘exposure’ is a pre-existing clinical condition rather than a trial-related intervention.<br><br>**Unblinding of blinded assessors** : Unblinding of the outcome assessors is only permissible after the completion of all data extraction and scoring for the primary and secondary outcomes. The final reveal of group assignments for the purpose of statistical analysis will occur only after the database lock, once all thermal and motor data have been cleaned and validated.	25

Methods: Data collection, management, and analysis			
Data collection methods	25a	**Personnel involved in assessment and collection** :<br>Clinical data and questionnaires : Collected and assessed by trained clinical investigators during hospital visits.<br>Behavioral pain (Neonatal Pain and Discomfort Scale (EDIN)) : Scored by trained clinical investigators based on direct observation.<br>Infrared thermal imaging : Data acquisition is performed by trained investigators, while extraction and assessment are performed by a specialized expert and independently reviewed by a second investigator.<br>Motor outcomes (General Movements) : Videos are recorded by investigators (in-hospital) or legal guardians (at home) and assessed by two certified advanced assessors.<br>Developmental outcomes (Bayley-IV scales) : Administered by trained researchers and scored by a blinded external observer.<br><br>**Mode of data collection** :<br>- Data are primarily entered into a pseudonymized electronic Case Report Form (e-CRF).<br>- Biological results from High-Performance Liquid Chromatography coupled with tandem Mass Spectrometry are transmitted from the Robert-Debré Hospital laboratory.<br>- Psychomotor Bayley-IV scores are calculated via the secure Pearson Clinical platform.<br>- Digital media (thermal recordings and motor videos) are stored as encrypted files.<br><br>**Data collection instruments (reliability and validity)** :<br>Neonatal Pain and Discomfort Scale (EDIN) : A validated behavioral tool with high internal validity (0.92) and reliability (0.69) for prolonged pain.<br>ColiQ® questionnaire : Validated instrument for assessing crying severity and its impact on families.<br>Infrared thermal imaging : Measurement validity supported by correlation with laser Doppler imaging ; uses a high-sensitivity FLIR® T630sc camera (sensitivity: 0.04°C–0.08°C).<br>General Movements Assessment : A standardized, non-invasive method with excellent inter-rater reliability and strong predictive validity for neurodevelopmental outcomes.<br>Bayley Scales of Infant and Toddler Development (Bayley-IV): Recognized as the gold standard for psychomotor assessment.<br><br>**Processes to promote data quality** :<br>Training and certification : Assessors for motor and developmental outcomes hold specialized certifications (e.g., GM Trust advanced level).<br>Duplicate/independent measurements : Thermal image extraction and motor video scoring are performed independently by two assessors, with discrepancies resolved by consensus.<br>Environmental standardization : Strict controls for thermal imaging, including dedicated rooms at 23°C ± 1°C and 50%–60% humidity, and standardized tripod positioning (1.60 m).<br>Procedural standardization : Use of standardized instructions for parents for home-recorded videos to minimize protocol deviations.	16–20, 25

		**Access to data collection forms** : All relevant forms (Neonatal Pain and Distress Scale, General Movements Scores, Bayley-IV, and the 7-day crying diary) are available in the supporting information.	
	25b	**Retention strategies to promote complete follow-up** :<br>Automated reminders : To ensure the timely submission of home-recorded motor videos, parents receive automated SMS reminders a few days before each required interval (days 15, 30, and 45 post-Visit 1).<br>Information and transparency : Families are provided with an information package including an explanatory brochure and access to the study website (https://sites.google.com/view/neotherm-fr). This ongoing access to project information is intended to maintain parental engagement and trust.<br>Reducing travel burden : By allowing parents to film their infant's General Movements at home using standardized instructions, the study minimizes the number of necessary hospital visits, thereby reducing the practical burden on families during the first months of life.<br><br>**Data collection for discontinued participants**<br>Participants who discontinue the study or whose legal guardians withdraw consent will not undergo any further study-specific assessments after the date of withdrawal. Data collected before withdrawal will be retained and used unless the legal guardians explicitly request their deletion, in accordance with the consent provided, the General Data Protection Regulation (GDPR), and applicable French regulations. If deletion is requested and legally applicable, no further use of the participant's data will be made.<br><br>**Recording reasons for non-adherence and non-retention**<br>Non-retention (withdrawal): In accordance with French regulatory requirements (RIPH2), legal guardians may withdraw their child from the study at any time without providing a reason and without any consequences for the child's medical care. Whenever possible, the reason for withdrawal will be documented in the e-CRF if voluntarily provided by the legal guardians; however, no participant or parent will be required to justify their decision.	13–15
Data management	26	**Data entry and coding** :<br>- Primary clinical and assessment data are entered into a pseudonymized electronic Case Report Form (e-CRF).<br>- Measures to reduce errors : To ensure data integrity, primary and secondary outcomes involving subjective interpretation (infrared thermal imaging and General Movements Assessment) are subject to independent double scoring by two experts. Any discrepancies are resolved via a formal consensus process before final entry into the study database.<br><br>**Data security** :<br>Confidentiality : All participant data are extracted using a unique study code; no directly identifiable information (names or addresses) is included in the analysis files.<br>Access control : Access to the database is strictly limited to authorized personnel through individual personal credentials.	26

<table>
<tr><td></td><td></td><td>Infrastructure : Electronic data are hosted on HDS-certified servers (Health Data Hosting) in compliance with the General Data Protection Regulation (GDPR) and French National Commission for Information Technology and Civil Liberties (CNIL) regulations.
Media security : Digital recordings, including family videos and thermal images, are stored on secure hard drives.

Data storage and timeframe :
- In accordance with French regulatory requirements for RIPH2 studies, all source documents and electronic data will be securely archived for a period of fifteen years.
- Physical records (e.g., signed consent forms) are archived at the Saint-Denis Hospital center.</td><td></td></tr>
<tr><td>Statistical methods</td><td>27a</td><td>Main analysis methods for statistical comparison :
Primary outcome : The association between infrared thermal imaging parameters (including bilateral asymmetries and temperature gradients) and Neonatal Pain and Discomfort Scale (EDIN) scores will be assessed using Spearman correlation coefficients. Concordance and agreement between the two methods will be further illustrated using Bland-Altman plots.
Secondary outcomes : Differences between the ‘excessive crying’ group and the ‘physiological crying’ group regarding motor and developmental scores (General Movements Assessment and Bayley-IV scales) will be analyzed using the Mann–Whitney test for independent samples.
Longitudinal data : Changes in thermal markers and behavioral pain scores over the 2-month follow-up will be explored using linear mixed-effects models, incorporating group, time, and the group-by-time interaction as fixed effects, with the individual participant treated as a random effect.

Statistical software, effect measures and significance level: Thermal image preprocessing and extraction of quantitative thermal variables will be performed using a dedicated Python pipeline developed for the study. Statistical analyses will subsequently be conducted using R (R Foundation for Statistical Computing, Vienna, Austria). Given the pilot and exploratory nature of this study, emphasis will be placed on effect sizes (including Spearman's ρ and Cohen's d) with their 95% confidence intervals. Statistical significance will be assessed using two-sided tests with a significance level of $\alpha = 0.05$ ($p < 0.05$).</td><td>24–25</td></tr>
<tr><td></td><td>27b</td><td>Primary and secondary analysis populations :
- The primary analysis for convergent validity will follow a complete-case analysis approach. This population will include all enrolled infants who have complete observed data for both the infrared thermal imaging parameters and the Neonatal Pain and Discomfort Scale (EDIN) scores at the relevant assessment visits.
- For secondary longitudinal analyses (including motor and developmental trajectories), infants will be included if they have completed the baseline assessment and the follow-up measurements (e.g., Visit 2 and home videos).
- Analyses will be performed using observed outcome data only. No formal data imputation (such as multiple imputation) is currently planned for the primary analysis, although sensitivity analyses may be conducted if the volume of missing data is significant.</td><td>25</td></tr>
</table>

		**Exclusions from analysis** :<br>- Participants will be excluded from specific analyses only in the case of missing data for the required variables.<br>- To prevent classification bias, infants who presented with intermediate crying patterns (between 2 and 3 hours per day) during the screening phase were excluded at the enrollment stage and will not be part of any analysis population.<br><br>**Group for analysis** :<br>Participants will be analyzed strictly according to their initial clinical group allocation ('excessive crying' versus 'physiological crying') determined at Visit 1 based on the first 7-day behavior diary.<br>This group assignment will be strictly maintained for all primary and longitudinal analyses, regardless of whether the infant's crying symptoms improve, worsen, or resolve during the 2-month follow-up period. This approach is intended to preserve the longitudinal integrity of the cohort and analyze outcomes based on the initial clinical phenotype.	
	27c	H**ow missing data will be handled in the analysis** : Every effort will be made to minimize missing data through standardized data collection procedures and participant follow-up. Analyses will primarily be conducted on available data. The extent and pattern of missing data will be described for each outcome. Given the pilot nature of the study and the relatively small sample size, no formal multiple imputation is planned. Participants with missing data for a specific outcome will be excluded from the corresponding analysis only, while retaining all available data for other analyses.	25
	27d	**Methods for any additional analyses (e.g., subgroup and sensitivity analyses):** Pre-specified subgroup analyses will be limited to comparisons between infants with excessive crying and those with physiological crying. Exploratory analyses may also be performed according to the severity of excessive crying based on the ColiQ classification, where appropriate. Sensitivity analyses may be conducted to assess the robustness of the primary findings by excluding participants with incomplete outcome data or protocol deviations. Any additional analyses not specified a priori will be considered exploratory and clearly identified as such in the final report.	25
**Methods: Monitoring**			
Data monitoring committee	28a	A formal, independent Data monitoring committee is not planned for the NEOTHERM study.<br><br>**Rationale for the absence of a data monitoring committee** :<br>Study design : NEOTHERM is a prospective observational pilot study rather than an interventional randomized clinical trial involving a pharmaceutical or high-risk medical device.<br>Risk level : The study is categorized under French regulations as Type Two research involving human subjects (RIPH2), which is defined as interventional research involving only minimal risks and constraints (RIPH2/RIRCM).<br>No interim analyses : There are no planned interim analyses or formal stopping rules based on efficacy or safety that would require the oversight of an independent monitoring body.	26

		In place of a formal committee, trial oversight is ensured by :<br>Sponsor monitoring : The Clinical Research Unit of the Saint-Denis Hospital center (the study sponsor) has implemented a risk-proportionate monitoring plan. This includes on-site and remote monitoring visits to verify informed consent quality, and eligibility criteria adherence.<br>Ethics committee review : The study protocol and data management procedures have been reviewed and approved by the Île-de-France Committee for the Protection of Individuals (CPP).	
	28b	**Interim analyses** :<br>Planned analyses : No formal interim analyses for efficacy or safety are planned for the NEOTHERM study. The statistical analysis plan specifies that primary and secondary outcome analyses will be conducted only at the close-out phase after the database has been frozen.<br>Rationale : Given that this is a prospective observational pilot study with minimal risks, and no therapeutic intervention is being tested, the continuous monitoring of results for the purpose of early stopping is not required.<br>Access to results : Not applicable, as no interim results will be generated. The study team and investigators will remain focused on data collection and quality monitoring throughout the recruitment period.<br><br>**Stopping guidelines (trial and participant level)** :<br>Participant withdrawal : At the individual level, the primary stopping rule is the legal guardian's right to withdraw their child from the study at any time, for any reason, and without providing justification.<br>Trial-level termination : The protocol does not pre-specify formal statistical criteria for the early termination of the entire study, nor are there plans for sample size re-estimation or other adaptations during the trial.	26
Trial monitoring	29	**Monitoring approach** : The NEOTHERM study utilizes a risk-proportionate monitoring plan implemented by the study sponsor (Saint-Denis Hospital center). This approach combines on-site and remote monitoring activities to ensure participant safety and the integrity of the collected data.<br><br>**Scope of monitoring activities** : Monitoring activities involve direct access to all study sites, source documents, and individual participant data. Key activities include :<br>- Conducting a setup visit at each of the three inclusion centers.<br>- Verifying the quality and completeness of written informed consent.<br>- Confirming participant adherence to eligibility criteria.<br><br>**Personnel involved in monitoring** : Monitoring is performed by the Clinical Research Unit of the Saint-Denis Hospital center. Specifically, the Clinical Research Associate or Project Manager from the sponsor's unit (distinct from the clinical investigators) is responsible for these oversight tasks.	26; S1 Protocol, pp. 44–45
**Ethics**			

Research ethics approval	30	**Regulatory authorization** : Formal regulatory authorization has been obtained from the relevant French Human Protection Committee (Comité de Protection des Personnes II Île-de-France). The research is registered under the national unique identification number IDRCB 2025-A00767-42.<br><br>**Legal classification** : In compliance with the French regulatory framework (the Loi Jardé), the study is officially classified as type 2 research involving human subjects (RIPH2), which is defined as interventional research presenting minimal risks and burdens to the participants.<br><br>**Informed consent requirement** : Written informed consent from the infant's legal guardians must be obtained by the investigators prior to any study-related procedures or enrollment.	26
Protocol amendments	31	**Plans for communicating important protocol modifications to relevant parties :** Any substantial amendments to the protocol will be reviewed and approved by the study sponsor and the relevant Ethics Committee (CPP) before implementation, in accordance with applicable French regulations. Approved amendments will be communicated to all participating investigators and study sites, incorporated into the study documentation, and updated in the trial registration (ClinicalTrials.gov) and future publications, where applicable.	26; S1 Protocol, p. 50
Consent or assent	32a	Personnel obtaining consent : Formal informed consent is obtained by an authorized investigator listed on the delegation of duties form, in accordance with the approved protocol.<br>Camille Perrolet is an osteopath and a doctoral student in neurosciences, and Joëlle Provasi is a scientific lead and researcher. Both individuals are designated as trained clinical investigators for this study.<br><br>**Consent process for potential participants** :<br>1. Initial referral : Parents of eligible infants are identified and referred to the research team by the healthcare personnel at the participating neonatal units and specialized clinics.<br>2. Information package : Families are provided with a comprehensive information package, which includes an explanatory brochure, a link to the study website (https://sites.google.com/view/neotherm-fr), and the formal informed consent form accompanied by an explanatory information leaflet.<br>3. Discussion : A subsequent discussion is held between the research team and the parents to explain the objectives of the NEOTHERM project and to address any questions.<br>4. Enrollment visit : Written informed consent is collected in person by the investigator at the start of the enrollment visit (Visit 0), prior to the administration of any questionnaires or assessments.<br><br>**Paediatric assent** : Formal assent from the participants is not applicable. The study population consists of infants aged between 2 and 5 weeks corrected age who lack the developmental and decisional capacity to provide assent. Information regarding the study is provided verbally and in writing to the legal guardians at each visit to ensure their ongoing understanding of the research.<br><br>**Proxy consent for adults** : Not applicable. The NEOTHERM study is restricted to the infant population and does not include adult participants who lack decisional capacity.	12, 26; S1 Protocol, pp. 23–26

	32b	**Consent for ancillary use of data** : The participant information sheet and consent form includes a specific optional section (check-box) where legal guardians can explicitly grant or withhold their permission for the reuse of their child's pseudonymized data in future ancillary research projects. This mechanism ensures that participation in the main NEOTHERM study is not contingent upon agreement to secondary data use, in compliance with the General Data Protection Regulation (GDPR).<br><br>**Access and oversight for data reuse** : Even when parental consent is obtained, the reuse of data by external investigators remains strictly controlled. It requires a formal application to the coordinating investigator, approval by the study sponsor, the signature of a dedicated data-sharing agreement, and the acquisition of relevant ethical approvals for the new research question.<br><br>**Ancillary use of biological specimens** : In contrast to clinical data, the current protocol specifies that capillary hair samples collected for cortisol analysis are not intended for long-term storage or use in unspecified future ancillary studies. All biological samples will be destroyed at the conclusion of the NEOTHERM study in accordance with the procedures of the Robert-Debré Hospital Biological Resources Platform.<br><br>**Collection and storage for ancillary purposes :** Research data, including digital media and clinical assessments, are securely archived for a period of 15 years at the Saint-Denis Hospital center, facilitating their availability for authorized ancillary analyses for those participants whose guardians have provided the required optional consent.	26
Confidentiality	33	**Confidentiality during collection and maintenance** :<br>Pseudonymization : All participant data are collected and maintained using a unique study identifier (code). No directly identifying information, such as names or home addresses, is recorded in the final analytical datasets or the study database.<br>Infrastructure and hosting : Electronic data are hosted on Health Data Hosting (HDS)-certified servers, ensuring compliance with the General Data Protection Regulation (GDPR) and French National Commission for Information Technology and Civil Liberties (CNIL) regulations.<br>Digital media security : Infrared thermal recordings and motor assessment videos are labeled only with the participant's unique study identifier to prevent accidental identification.<br>Long-term maintenance : All pseudonymized data and source documents will be securely archived for a period of 15 years at the Saint-Denis Hospital center.<br><br>**Confidentiality during data transmission** :<br>Transmission to the sponsor : Data transmitted to the study Sponsor (Saint-Denis Hospital center) for monitoring or auditing purposes are strictly pseudonymized. Names and addresses are never transmitted or displayed in clear text.	26; S1 Protocol, pp. 44–49

		<u>External assessment platforms</u> : Developmental scores from the Bayley-IV scales are calculated via the secure Pearson Clinical platform. Data transmitted to this platform are pseudonymized using only the study identifier, without any personally identifiable information.<br><u>Biological specimen transport</u> : Capillary hair samples are labeled with a unique code and the collection date and are transported to the analytical laboratory at Robert-Debré Hospital with full traceability.<br><u>External data requests</u> : Requests for data sharing from external parties are subject to the signing of a formal data-sharing agreement, which mandates the continued protection of participant confidentiality and compliance with the General Data Protection Regulation (GDPR).	
Ancillary and post-trial care	34	**Compensation for trial-related harms**: The study sponsor (Saint-Denis Hospital Center) has subscribed to a comprehensive insurance policy with Lloyd's Insurance Company SA (8 rue Lamennais, 75008 Paris, France), policy No. B1339CTLICNWL24-54, covering the entire duration of the research. This insurance policy guarantees the Sponsor's civil liability as well as that of all investigators involved in the study. In accordance with French regulatory requirements for category 2 research involving human participants (RIPH2), the Sponsor ensures compensation for any harmful consequences directly resulting from participation in the research for participants and their legal beneficiaries, irrespective of fault, unless the damage is proven to result from the participant's own actions. The insurance contract was finalized prior to the initiation of the study.<br><br>**Ancillary care during the trial** :<br>Although NEOTHERM is a prospective observational study rather than an interventional trial, provisions are made for the communication of individual qualitative feedback to the legal guardians. This feedback regarding the child's motor and behavioral development is provided when the observations are judged to be clinically relevant for the infant's health or neurodevelopmental follow-up.<br><br>**Post-trial care and benefits** :<br>At the conclusion of the research, a summary of the collective study results will be made available to all participating families via the study website (https://sites.google.com/view/neotherm-fr). The results will also be disseminated to the scientific and healthcare communities through publications and oral presentations.<br><br>**Host community benefits** : No specific benefits or care programs are currently planned for the host communities beyond the dissemination of scientific knowledge intended to improve the future clinical management of infant pain.	S1 Protocol, pp. 49–50

*We strongly recommend reading this checklist in conjunction with the SPIRIT 2025 Explanation and Elaboration and the SPIRIT 2025 Expanded Checklist for important clarifications on all the items. We also recommend reading relevant SPIRIT extensions. See www.consort-spirit.org

Citation: Chan A-W, Boutron I, Hopewell S, Moher D, Schulz KF, et al. SPIRIT 2025 statement: updated guideline for protocols of randomised trials. BMJ 2025;389:e081477. https://dx.doi.org/10.1136/bmj-2024-081477

© 2025 Chan A-W et al. This is an Open Access article distributed under the terms of the Creative Commons Attribution License (https://creativecommons.org/licenses/by/4.0/), which permits unrestricted use, distribution, and reproduction in any medium, provided the original work is properly cited.
